# Synthesis and *In Silico* and *In Vitro* Antitumor Potential of Novel Organochalcogens Derived from Azidothymidine and 2,4,6-Trichloro-1,3,5-triazine

**DOI:** 10.1021/acsomega.6c07643

**Published:** 2026-10-10

**Authors:** Nayra Salazar Rocha, Sidne Rodrigues da Silva, Brenda Bomm Carvalho, Marcelo Wendt, Luciano Dornelles, Luciana Filippin Cossetin, Bianca Costa Maia-do-Amaral, Daniele Rubert Nogueira-Librelotto, Raquel Mello da Rosa, Alessandro de Souza Prestes, João Cândido Pilar Mayer, Nilda Vargas Barbosa, João Batista Teixeira da Rocha, Oscar Endrigo Dorneles Rodrigues

**Affiliations:** † LabSelen-NanoBio − Department of Chemistry, Federal University of Santa Maria - Universidade Federal de Santa Maria, Av. Roraima, 1000, CEP: 97105-900 Santa Maria, RS, Brazil; ‡ Laboratory of In Vitro Pharmaceutical Testing and Assays, Department of Industrial Pharmacy, Federal University of Santa Maria, Av. Roraima, 1000, CEP: 97105-900 Santa Maria, RS, Brazil; § Department of Biochemistry and Molecular Biology, Federal University of Santa Maria, Santa Maria, Av. Roraima, 1000, CEP: 97105-900 Santa Maria, RS, Brazil; ∥ Laboratory of Molecular Catalysis, Institute of Chemistry, Federal University of Rio Grande do Sul, Av. Bento Gonçalves, 9500, CEP: 91509-900 Porto Alegre, RS, Brazil

## Abstract

The preparation of a new class of organochalcogen compounds derived from arylchalcogeno aminothymidine (ACAT) and 2,4,6-trichloro-1,3,5-triazine is described. The compounds were obtained in good yields via aromatic nucleophilic substitution reactions between ACAT derivatives and the triazine ring. The developed protocol selectively afforded monosubstituted derivatives, avoiding the formation of di- and trisubstituted isomers. Antitumor activity was evaluated against HT-29 (human colorectal adenocarcinoma) and NCI/ADR-RES (multidrug-resistant human ovarian carcinoma) cell lines using the MTT assay. Sulfur- and selenium-containing derivatives showed only moderate cytotoxic effects, reducing cell viability mainly at the highest tested concentration (100 μM). In contrast, tellurium-containing derivatives exhibited pronounced cytotoxic activity, with minimum IC_50_ values of 9.90 μM in HT-29 cells and 12.18 μM in NCI/ADR-RES cells, demonstrating notable efficacy against multidrug-resistant tumor cells. In addition, the antioxidant properties of the compounds were investigated *in vitro*. Tellurium derivatives displayed significant antioxidant activity, as demonstrated by inhibition of lipid peroxidation in the TBARS assay and by thiol-peroxidase (GPx-like) activity. A possible mechanism of action based on tubulin polymerization inhibition was investigated by means of a molecular docking study. The results demonstrate the potential of these novel organochalcogen compounds as antitumor agents, highlighting tellurium incorporation as a promising strategy to overcome multiple drug resistance (MDR) in cancer.

## Graphical abstract

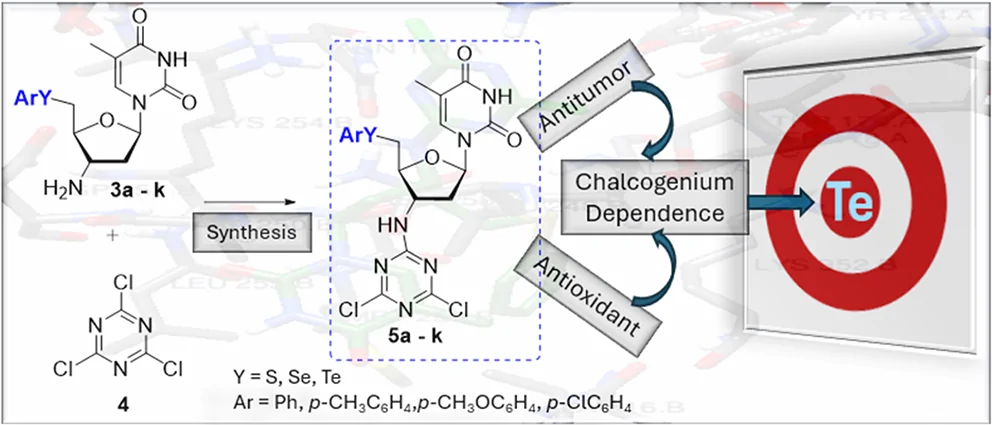

## 1 Introduction

Organochalcogens are compounds that contain chalcogen atoms (sulfur, selenium, and tellurium) in their structure and are widely applied in organic and medicinal chemistry, particularly due to their biological properties.

These compounds generally exhibit antioxidant,^1^ antimicrobial,^2^ and anti-inflammatory^3^ activities. Usually, the introduction of a chalcogen moiety into an organic compound can increase the biological activity of similar molecules that do not contain these atoms.^4^ Some of the notable advantages of the structure–activity relationship of these combined molecules include (i) the reduction of adverse effects due to the combination of medicinal and electronic properties, for instance, antioxidant activity may help avoid the side effects associated with ROS generated by conventional anticancer therapies,^5–7^ and (ii) the reduction of tumor cell resistance to multiple drugs (MDR).^8–10^

Additionally, the therapeutic properties of nucleoside analogs have been expanded, largely due to the variety of specific chemical modifications made to their structures, which can be designed based on key interactions with biological targets.^11,12^ These modifications have led to the development of potent drugs such as 3′-azido-3-deoxythymidine (azidothymidine or AZT), which exhibits antiretroviral properties by inhibiting the activity of reverse transcriptase.^13,14^ Furthermore, nucleoside derivatives have shown inhibitory activity against the RNA polymerase of the SARS-CoV-2 virus.^15–17^ Additionally, they are promising candidates for the development of antitumor therapies.^11,18,19^ In this context, chalcogen nucleosides appear as an interesting class of compounds. These molecules exhibit notable properties, particularly from a biological perspective, as they combine the characteristics of nucleosides with the intriguing properties of chalcogen atoms. For instance, the literature has reported the antitumor activity of chalcogen-containing nucleosides, highlighting the influence of the chalcogen atom on this bioactivity.^20,21^ In some cases, tellurium derivatives tend to exhibit stronger growth-inhibitory effects against a variety of tumor cell lineages while showing limited toxicity in the *in vivo* and *in vitro* assays performed.^11,22,23^ Moreover, the insertion of chalcogens into the chemical structure of other molecules, such as nucleosides, can enhance their bioavailability and therapeutic efficacy in antiviral^16,24^ and antitumor applications.^11,22^

On the other hand, substitution reactions involving the 2,4,6-trichloro-1,3,5-triazine nucleus have been employed in the synthesis of molecules with anticancer,^25^ antiviral,^26^ and antimicrobial^27^ activities. Recently, new triazine-naphthalimide analogues have been described, demonstrating activity in the cytoplasmic membrane of multidrug-resistant *Staphylococcus aureus*, with results comparable to those of chloromycin and amoxicillin. Additionally, toxicity assays were performed against 60 cancer cell lines, in which these derivatives exhibited promising antitumor activity.^28^

Considering the antitumor bioactive potential of AZT analogues and the need for low-toxicity drugs, our research group has dedicated the past few years to the synthesis and the biological evaluations of chalcogen nucleosides. Thus, we describe herein the synthesis of a library of novel compounds derived from 5′-arylchalcogeno-3-aminothymidine combined with the heterocyclic 2,4,6-trichloro-1,3,5-triazine, as well as the antioxidant and antiproliferative activities against sensitive (HT-29, colorectal adenocarcinoma) and resistant/MDR (NCI/ADR-RES, human ovarian carcinoma) tumor cell lines (Figure 1).

**Figure 1:**
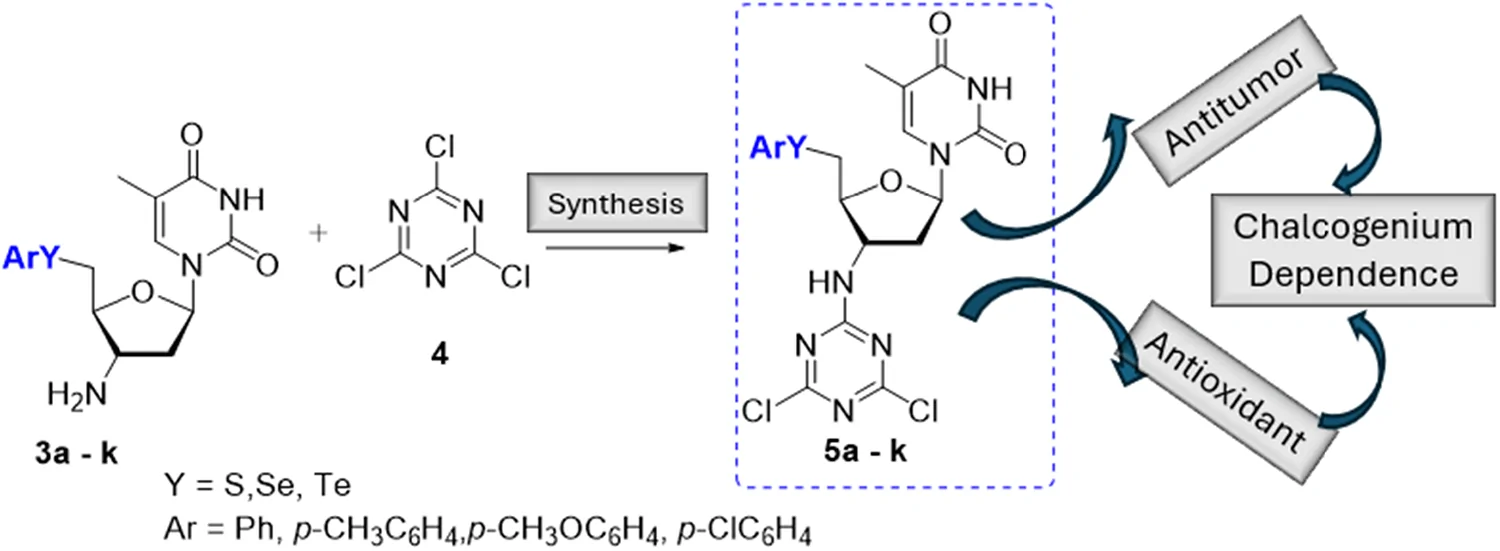
Synthesis and biological evaluation of the novel 5′-arylchalcogenyl-(3-*N*-4,6-dichloro-1,3,5-triazin-2-yl)-thymidine.

## 2 General Methods

### 2.1 Synthesis of 5′-*O*-(Mesyl)­zidovudine (2)^11^

In the two-necked round-bottom flask under an argon atmosphere, 1 mmol zidovudine (1) was added in 7 mL of THF. After the dissolution of zidovudine, the system was cooled to 0 °C, and triethylamine (1.5 mmol) was added. After 10 min, while still at 0 °C, mesyl chloride (1.1 mmol) diluted in 3 mL of THF was added dropwise. Then, the ice bath was removed and the system allowed to react for 2 h at room temperature. After this period, the reaction was extracted with a saturated solution of NH_4_Cl (20 mL), and the organic phase was extracted with dichloromethane (3 × 20 mL). Organic phases were combined and dried over MgSO_4_. The solvent was evaporated under reduced pressure, and the product crystallized in ethyl acetate and dried in a high-vacuum pump. Mesylate (2) yield obtained was of 92%.

### 2.2 Preparation of Arylchalcogeno-aminothymidines (**3a**–**3k**)^11,16^

In a two-necked round-bottom flask under an argon atmosphere, diaryl dichalcogenide (0.5 mmol), THF (4 mL) and ethanol (3 mL) were added. Afterward, NaBH_4_ (5.0 eq., 5 mmol, 0.185 g) was added, and the reaction was stirred until the disappearance of the color. Subsequently, 5′-*O*-(mesyl)­zidovudine (1 mmol) dissolved in THF (3 mL) was added dropwise to the reaction flask. The system was heated at reflux for 24 hours. After the completion of the reaction, the mixture was quenched with a saturated solution of NH_4_Cl and extracted with ethyl acetate. The solvent was evaporated under reduced pressure, and the crude product was purified by chromatographic column employing a gradient of a mixture of dichloromethane and ethanol (until 70:30) as solvent.

### 2.3 Preparation of 5′-Arylchalcogenyl-(3-*N*-4,6-dichloro-1,3,5-triazin-2-yl)-thymidine **5a**–**5k**

In a 50 mL round-bottom flask containing 0.5 mmol of cyanuric chloride in 10 mL of acetone (0 °C), 0.5 mmol of the corresponding 5′-arylchalcogenyl-3-aminothymidine solubilized in 6 mL of the same solvent was slowly added, followed by 0.5 mmol of the base *N*,*N*-di-isopropylethylamine. The reaction mixture was stirred at 0 °C for 1 h. The reaction was monitored by thin-layer chromatography. After the completion of the reaction, the product was extracted with dichloromethane (3 × 20 mL), using approximately 60 mL of a saturated NaCl solution slightly acidified with 0.5 mL of HCl 0.1 M as the aqueous phase. The product was dried over anhydrous MgSO_4_, filtered, and the solvent evaporated, providing the crude products. They were purified in a chromatographic column using silica gel with dichloromethane and ethanol (97:3) as eluent, with yields between 70 and 90%.

#### 2.3.1 **5a**: 5′-Seleno-(phenyl)-(3-*N*-4,6-dichloro-1,3,5-triazin-2-yl)-thymidine

##### 2.3.1.1 Physical Properties

White solid. Melting point: 240–243 °C. Yield: 90%. **HRMS** [TOF MS ES+] *m*/*z* calculated for C_19_H_18_Cl_2_N_6_O_3_Se [M + H]^+^: 529.0061; found [M + H]^+^: 529.0060. ^**1**^ **H NMR (CDCl**_**3**_ **, 600 MHz), δ (ppm)**: 10.06 (s, 1H), 9.65–9.64 (d, *J* = 7.5 Hz, 1H), 7.62–7.58 (m, 2H), 7.46 (s, 1H), 7.33–7.23 (m, 3H), 6.55–6.53 (m, 1H), 4.54–4.52 (m, 1H), 4.31–4.30 (q, *J* = 3.9 Hz, 1H), 3.52–3.48 (m, 1H), 3.48–3.42 (m, 1H), 2.41–2.24 (m, 2H), 1.83 (s, 3H). ^**13**^ **C NMR (CDCl**_**3**_ **, 150 MHz), δ (ppm)**: 171.1, 170.6, 165.1, 162.7, 152.2, 134.1, 131.7, 129.6, 127.3, 113.0, 85.3, 84.6, 54.7, 37.1, 31.8, 12.7.

#### 2.3.2 **5b**: 5′-Telluro-(phenyl)-(3-*N*-4,6-dichloro-1,3,5-triazin-2-yl)-thymidine

##### 2.3.2.1 Physical Properties

White solid. Melting point: 210–214 °C. Yield: 72%. **HRMS** [APCI-MS (−) (+)] *m*/*z* calculated for C_19_H_18_Cl_2_N_6_O_3_Te [M – H]^−^: 576.9801; found [M – H]^−^: 576.9788. ^**1**^ **H NMR (CDCl**_**3**_ **, 600 MHz), δ (ppm)**: 10.07 (s, 1H), 9.55 (d, *J* = 7.6 Hz, 1H), 7.83 (d, *J* = 7.4 Hz, 2H), 7.49 (s, 1H), 7.36–7.26 (m, 3H), 6.60–6.55 (m, 1H), 4.51 (t, *J* = 8.2 Hz, 1H), 4.37– 4.33 (m, 1H), 3.54–3.47 (m, 1H), 3.45–3.39 (m, 1H), 2.47–2.40 (m, 1H), 2.36–2.30 (m, 1H), 1.92 (s, 3H). ^**13**^ **C NMR (CDCl**_**3**_ **, 150 MHz), δ (ppm)**: 171.1, 170.6, 165.1, 162.7, 152.1, 137.5, 134.3, 129.6, 128.1, 113.0, 111.3, 85.8, 84.4, 56.0, 37.1, 13.5, 12.7.

#### 2.3.3 **5c**: Thio-(phenyl)-(3-*N*-4,6-dichloro-1,3,5-triazin-2-yl)-thymidine

##### 2.3.3.1 Physical Properties

Yellow solid. Melting point: 229–231 °C. Yield: 86%. **HRMS** [APCI-MS (−) (+)] *m*/*z* calculated for C_19_H_18_Cl_2_N_6_O_3_S [M – H]^−^: 479.0460; found [M – H]^−^: 479.0491. ^**1**^ **H NMR (CDCl**_**3**_ **, 600 MHz), δ (ppm)**: 10.09 (s, 1H), 9.69 (d, *J* = 7.3 Hz, 1H), 7.53–7.48 (m, 3H), 7.36 (t, *J* = 7.8 Hz, 2H), 7.30 (s, 1H), 6.59 (dd, *J* = 9.9, 4.9 Hz, 1H), 4.62 (t, *J* = 7.9 Hz, 1H), 4.32 (s, 1H), 3.60–3.46 (m, 2H), 2.48–2.42 (m, 1H), 2.38–2.25 (m, 1H), 1.85 (s, 3H). ^**13**^ **C NMR (CDCl**_**3**_ **, 150 MHz), δ (ppm)**: 171.1, 170.7, 165.1, 162.7, 152.2, 135.4, 134.0, 129.3, 128.8, 126.6, 113.0, 85.2, 84.8, 54.2, 37.9, 37.1, 12.6.

#### 2.3.4 **5d**: 5′-Seleno-(4-chlorophenyl)-(3-*N*-4,6-dichloro-1,3,5-triazin-2-yl)-thymidine

##### 2.3.4.1 Physical Properties

White solid. Melting point: 247–249 °C. Yield: 80%. **HRMS** [APCI-MS (−) (+)] *m*/*z* calculated for C_19_H_17_Cl_3_N_6_O_3_Se [M – H]^−^: 560.9516; found [M – H]^−^: 560.9514. ^**1**^ **H NMR (CDCl**_**3**_ **, 600 MHz), δ (ppm)**: 10.09 (s, 1H), 9.69 (d, *J* = 7.2 Hz, 1H), 7.53–7.48 (m, 2H), 7.36 (t, *J* = 7.7 Hz, 2H), 7.30 (s, 1H), 6.59 (dd, *J* = 9.5, 4.8 Hz, 1H), 4.62 (t, *J* = 7.9 Hz, 1H), 4.32 (s, 1H), 3.60–3.47 (m, 2H), 2.48–2.43 (m, 1H), 2.33–2.25 (m, 1H), 1.85 (s, 3H). ^**13**^ **C NMR (CDCl**_**3**_ **, 150 MHz), δ (ppm)**: 169.9, 169.1, 165.6, 164.2, 150.8, 136.8, 133.3, 131.9, 129.4, 110.3, 83.8, 82.2, 54.8, 36.1, 31.3, 30.1, 12.5.

#### 2.3.5 **5e**: 5′-Seleno-(4-methylphenyl)-(3-*N*-4,6-dichloro-1,3,5-triazin-2-yl)-thymidine

##### 2.3.5.1 Physical Properties

White solid. Melting point: 252–253 °C. Yield: 87%. **HRMS** [APCI-MS (−) (+)] *m*/*z* calculated for C_20_H_20_Cl_2_N_6_O_3_Se [M – H] ^–^: 542.0139; found [M – H] ^–^: 542.0116. ^**1**^ **H NMR (CDCl**_**3**_ **, 600 MHz), δ (ppm)**: 10.39 (s, 1H), 9.59 (d, *J* = 7.0 Hz, 1H), 7.57–7.53 (m, 2H), 7.49 (s, 1H), 6.87–6.80 (m, 2H), 6.52 (dd, *J* = 9.6, 4.8 Hz, 1H), 4.63–4.45 (m, 1H), 4.24 (d, *J* = 22.5 Hz, 1H), 3.43–3.28 (m, 2H), 2.45–2.39 (m, 1H), 2.37–2.20 (m, 1H), 1.89 (s, 3H). ^**13**^ **C NMR (CDCl**_**3**_ **, 150 MHz), δ (ppm)**: 171.6, 171.0, 165.1, 163.1, 159.6, 152.1, 134.2, 133.7, 118.8, 114.8, 113.5, 85.4, 84.7, 55.3, 54.7, 37.1, 32.9, 12.7.

#### 2.3.6 **5f**: 5′-Seleno-(4-methoxyphenyl)-(3-*N*-4,6-dichloro-1,3,5-triazin-2-yl)-thymidine

##### 2.3.6.1 Physical Properties

White solid. Melting point: 249–250 °C. Yield: 82%. **HRMS** [APCI-MS (−) (+)] *m*/*z* calculated for C_20_H_20_Cl_2_N_6_O_4_Se [M + H]^+^: 559.0167; found [M + H]^+^: 559.0157. ^**1**^ **H NMR (CDCl**_**3**_ **, 600 MHz), δ (ppm)**: 10.08 (s, 1H), 9.66 (d, *J* = 7.5 Hz, 1H), 7.59 (d, *J* = 8.2 Hz, 2H), 7.52 (s, 1H), 6.88 (d, *J* = 8.1 Hz, 2H), 6.57 (dd, *J* = 10.0, 4.8 Hz, 1H), 4.58 (t, *J* = 8.1 Hz, 1H), 4.29 (s, 1H), 3.83 (s, 3H), 3.46–3.40 (m, 1H), 3.37–3.31 (m, 1H), 2.46–2.40 (m, 1H), 2.32–2.24 (m, 1H), 1.94 (s, 3H). ^**13**^ **C NMR (CDCl**_**3**_ **, 150 MHz), δ (ppm)**: 171.0, 170.6, 165.1, 162.7, 159.6, 152.2, 134.7, 134.0, 119.2, 115.2, 113.0, 85.5, 84.6, 55.3, 54.6, 37.1, 32.9, 12.7.

#### 2.3.7 **5g**: 5′-Telluro-(4-methylphenyl)-(3-*N*-4,6-dichloro-1,3,5-triazin-2-yl)-thymidine

##### 2.3.7.1 Physical Properties

White solid. Melting point: 198–201 °C. Yield: 72%. **HRMS** [APCI-MS (−) (+)] *m*/*z* calculated for C_20_H_20_Cl_2_N_6_O_3_Te [M – H]^−^: 590.9958; found [M – H]^−^: 590.9944. ^**1**^ **H NMR (CDCl**_**3**_ **, 600 MHz), δ (ppm)**: 9.91 (s, 1H), 9.31 (d, *J* = 7.18 Hz, 1H), 7.68 (d, *J* = 7.73 Hz, 2H), 7.46 (d, *J* = 1.41 Hz, 1H), 7.06–7.04 (m, 2H), 6.53 (dd, *J* = 9.37, 5.07 Hz, 1H), 4.48–4.46 (m, 1H), 4.29–4.27 (m, 1H), 3.42 (dd, *J* = 12.77, 5.24 Hz, 1H), 3.33 (dd, *J* = 12.75, 5.06 Hz, 1H), 2.40–2.37 (m, 1H), 2.34 (s, 3H), 2.29–2.27 (m, 1H), 1.90 (m, 3H). ^**13**^ **C NMR (CDCl**_**3**_ **, 150 MHz), δ (ppm)**: 171.1, 170.6, 165.1, 162.8, 152.0, 138.3, 138.0, 134.4, 130.5, 112.9, 107.0, 85.8, 84.4, 55.9, 37.2, 21.2, 13.3, 12.7.

#### 2.3.8 **5h**: 5′-Thio-(4-methoxyphenyl)-(3-*N*-4,6-dichloro-1,3,5-triazin-2-yl)-thymidine

##### 2.3.8.1 Physical Properties

White solid. Melting point: 205–207 °C. Yield: 82%. **HRMS** [APCI-MS (−) (+)] *m*/*z* calculated for C_20_H_20_Cl_2_N_6_O_4_S [M – H]^−^: 509.0566; found [M – H]^−^: 509.0596. ^**1**^ **H NMR (CDCl**_**3**_ **, 600 MHz), δ (ppm)**: 10.09 (s, 1H), 9.70 (d, *J* = 6.7 Hz, 1H), 7.54 (s, 1H),7.48 (d, *J* = 8.0 Hz, 2H), 6.90 (d, *J* = 8.8 Hz, 2H), 6.60–6.55 (m, 1H), 4.62 (t, *J* = 8.1 Hz, 1H), 4.15 (q, *J* = 7.2 Hz, 1H), 3.83 (s, 3H), 3.49–3.44 (m, 1H), 3.39–3.33 (m, 1H). 2.48–2.43 (m, 1H), 2.30–2.24 (m, 1H), 1.92 (s, 3H). ^**13**^ **C NMR (CDCl**_**3**_ **, 150 MHz), δ (ppm)**: 171.0, 170.7, 165.1, 162.8, 159.2, 152.2, 134.0, 132.5, 124.4, 115.0, 113.0, 85.3, 84.9, 55.4, 54.1, 40.0, 37.0, 12.7.

#### 2.3.9 **5i**: 5′-Thio-(4-chlorophenyl)-(3-*N*-4,6-dichloro-1,3,5-triazin-2-yl)-thymidine

##### 2.3.9.1 Physical Properties

White solid. Melting point: 240–243 °C. Yield: 74%. **HRMS** [APCI-MS (−) (+)] *m*/*z* calculated for C_20_H_20_Cl_3_N_6_O_3_S [M + H]^+^: 515.0227; found [M + H]^+^: 515.0221. ^**1**^ **H NMR (CDCl**_**3**_ **, 600 MHz), δ (ppm)**: 11.34 (s, 1H), 9.48 (d, *J* = 7.6 Hz, 1H), 7.52 (s, 1H), 7.43–7.35 (m, 2H), 7.34–7.30 (m, 2H), 6.21–6.13 (m, 1H), 4.61–4.49 (m, 1H), 4.04 (q, *J* = 5.8 Hz, 1H), 3.55–3.41 (m, 1H), 3.41–3.34 (m, 1H), 2.49–2.39 (m, 1H), 2.31–2.24 (m, 1H), 1.76 (s, 3H). ^**13**^ **C NMR (CDCl**_**3**_ **, 150 MHz), δ (ppm)**: 169.9, 169.1, 165.6, 164.1, 150.8, 137.7, 135.5, 130.9, 130.1, 129.3, 110.3, 84.0, 81.8, 53.9, 36.0, 35.6, 12.1.

#### 2.3.10 **5j**: 5′-Thio-(4-methyl)-(3-*N*-4,6-dichloro-1,3,5-triazin-2-yl)-thymidine

##### 2.3.10.1 Physical Properties

White solid. Melting point: 241–246 °C. Yield: 87%. **HRMS** [APCI-MS (−) (+)] *m*/*z* calculated for C_20_H_20_Cl_2_N_6_O_3_S [M + H]^+^: 495.0773; found [M + H]^+^: 495.0767. ^**1**^ **H NMR (CDCl**_**3**_ **, 600 MHz), δ (ppm)**: 9.98 (s, 1H), 9.55 (d, *J* = 7.4 Hz, 1H), 7.48 (d, *J* = 1.5 Hz, 1H), 7.40–7.36 (m, 1H), 7.14 (d, *J* = 8.0 Hz, 2H), 6.54 (dd, *J* = 9.7, 4.9 Hz, 1H), 4.61–4.56 (m, 1H), 4.28–4.23 (m, 1H), 3.52–3.46 (m, 1H), 3.45–3.39 (m, 1H), 2.45–2.38 (m, 1H), 2.32 (s, 3H), 2.29–2.20 (m, 1H), 1.84 (s, 3H). ^**13**^ **C NMR (CDCl**_**3**_ **, 150 MHz), δ (ppm)**: 171.1, 170.7, 165.1, 162.7, 152.1, 136.9, 134.1, 131.6, 130.1, 129.4, 113.0, 85.2, 84.8, 54.1, 38.4, 37.1, 21.0, 12.6.

#### 2.3.11 **5k**: 5′-Telluro-(4-chlorophenyl)-(3-*N*-4,6-dichloro-1,3,5-triazin-2-yl)-thymidine

##### 2.3.11.1 Physical Properties

White solid. Melting point: 205–210 °C. Yield: 70%. **HRMS** [APCI-MS (−) (+)] *m*/*z* calculated for C_19_H_17_Cl_3_N_6_O_3_Te [M – H]^−^: 610.9412; found [M – H]^−^: 610.9390. ^**1**^ **H NMR (CDCl**_**3**_ **, 600 MHz), δ (ppm)**: 11.33 (s, 1H), 9.45 (d, *J* = 7.7 Hz, 1H), 7.66 (d, *J* = 8.2 Hz, 2H), 7.57 (d, *J* = 1.5 Hz, 1H). 7.21 (d, *J* = 8.4 Hz, 2H), 6.16 (t, *J* = 6.8 Hz, 1H), 51–4.47 (m, 1H), 4.09–4.06 (q, *J* = 5.8 Hz, 1H), 2.51–2.47 (m, 1H), 2.36–2.31 (m, 1H), 1.80–1.79 (m, 2H), 1.75 (s, 3H). ^**13**^ **C NMR (CDCl**_**3**_ **, 150 MHz), δ (ppm)**: 169.9, 169.1, 165.6, 164.1, 150.4, 138.8, 136.9, 132.9, 129.5, 111.6, 110.2, 83.5, 82.9, 55.7, 36.2, 12.9, 12.6.

### 2.4 Antioxidant Assays

#### 2.4.1 Lipid Peroxidation Assay

Lipid peroxidation was estimated using the thiobarbituric acid reactive species (TBARS) method, as described by Ohkawa et al.,^29^ with minor modifications. Briefly, 7.5 mg/mL phosphatidylcholine was incubated for 60 min at 37 °C with 200 μM compounds in a medium containing 50 mM of Tris buffer (pH 7.4) with or without 100 μM FeSO_4_ (used as a pro-oxidant agent). Acetic acid/HCL buffer (pH 3.4) and 0.6% TBA (pH 6.00) were added, and the color reaction was developed by incubating the samples for 60 min at 100 °C. After, curves were performed (1–25 μM) with compounds that prevented the lipid peroxidation at 200 μM. TBARS levels were measured at 532 nm and were expressed as a percentage of the control. A negative control was performed using DMSO (vehicle).

#### 2.4.2 Thiol-peroxidase-like Activity Assay

The catalytic activity of the compounds as an *in vitro* glutathione-peroxidase (GPx) model enzyme was evaluated according to the Iwaoka and Tomoda^30^ method, using 5 mM benzenethiol (PhSH) as substrate. The reduction of 5 mM H_2_O_2_ was monitored at 305 nm for 180 s. The compounds were initially tested at 200 μM, and a curve was performed at concentrations of 50, 20, 10, 5, and 2.5 μM with the compound(s) that exhibited thiol-peroxidase-like activity at a concentration of 200 μM. The activities of the compounds were compared with the activity of 200 μM (PhSe)_2_ (positive control). Negative control was performed using DMSO (vehicle).

#### 2.4.3 Statistical Analysis

Statistical analyses were performed using one-way or two-way ANOVA, followed by Tukey multiple tests when appropriate. Results were expressed as mean ± SEM of 3–4 independent experiments. Differences were considered statistically significant when *p* ≤ 0.05. The graphics were made using GraphPad Prism version 8.00 for Windows (GraphPad Software, La Jolla, California, USA).

### 2.5 Molecular Docking Procedures

Structure models of studied compounds were built in the Avogadro program and were energy-minimized by the UFF force field,^31^ included in the same program. All models were then optimized at the PM6 level^32^ within the MOPAC semiempirical quantum chemistry package. Optimized structures were prepared for docking procedures with the aid of the AutoDock Tools interface,^33^ in which structure roots and torsions were properly assigned. The resulting models were saved as PDBQT files. The tubulin protein structure was obtained directly from the RCSB PDB database with the code 5JCB. The macromolecule structure was opened in the AutoDock Tools interface, and a single αβ-dimer was selected for calculations. Original ligand structure and water molecules were excluded, while bound GTP molecules and associated magnesium ions were conserved, a structurally essential feature of the employed model. To the resulting structure, polar hydrogens were added according to the default program’s parameters, including all histidine residues. Protein structure was assigned with Kollman charges and was saved as a PDBQT file. The docking grid was centered at *X* = −17.463 Å, *Y* = 67.653 Å, and *Z* = −43.743 Å, with dimensions of *X*, *Y*, *Z* = 26.25 Å. Exhaustiveness value was adjusted to 50 in the configuration file, and the remaining docking parameters were used as default. Molecular docking calculations were run in the AutoDock Vina program.^34^ For validation, the original ligand YJTSF1 (code NV4) was redocked into the binding site with the same parameters employed for the other examples. After the analysis of the results, the most favorable pose of each example was selected for discussion. Results were visualized, and interaction diagrams were built in UCSF Chimera^35^ and in Discovery Studio interfaces.

### 2.6 *In Vitro* Antiproliferative Activity and Cytocompatibility Assay

#### 2.6.1 Materials

High-glucose (4.5 g/L) Dulbecco’s modified Eagle’s medium (DMEM), supplemented with l-glutamine (584 mg/L), 2,5-diphenyl-3-(4,5-dimethyl-2-thiazolyl) tetrazolium bromide (MTT), trypsin-EDTA solution, antibiotic solution of penicillin/streptomycin, and fetal bovine serum (FBS) were obtained from Sigma–Aldrich (São Paulo, SP, Brazil).

The HT-29 (human colorectal adenocarcinoma) cell line and the MDR human ovarian carcinoma cell line, NCI/ADR-RES, were used in this study to assess the antitumor activity. The L929 (mouse fibroblast) cell line was used to assess the cytocompatibility of the most active tested compound. The HT-29 and L929 cell lines were obtained from the Rio de Janeiro Cell Bank (BCRJ, Rio de Janeiro, Brazil), and the NCI/ADR-RES cell line was kindly donated by the University of Girona (Girona, Spain).

#### 2.6.2 Method

All cell lines were maintained in complete DMEM, supplemented with 10% (v/v) FBS. For NCI/ADR-RES cells, the medium was also supplemented with 1.0 μg/mL of DOX. Cells were cultured in 75 cm^2^ flasks at 37 °C in a humidified incubator with 5% CO_2_. Cultures were maintained until they reached approximately 80% confluence, then the cells were harvested, seeded into 96-well plates, and incubated for 24 h under the same conditions.

Both tumor cell lines were used to assess the potential *in vitro* antiproliferative effects of the organochalcogens at the following cell densities: HT-29 (5.5 × 10^4^ cells/mL) and NCI/ADR-RES (1 × 10^5^ cells/mL). After incubation, the medium was replaced with 100 μL of treatment solutions prepared in DMEM supplemented with 5% (v/v) FBS. The compounds were tested at concentrations of 5, 15, 30, 60, and 100 μM. Doxorubicin was used as a reference drug to compare the activity of the synthesized compounds against a conventional antitumor agent. Control cells received only DMEM with 5% (v/v) FBS. Cells were exposed to the treatments for 72 h, followed by a 3 h incubation at 37 °C in a 5% CO_2_ atmosphere with 0.5 mg/mL MTT solution prepared in DMEM without FBS. After incubation, the medium was removed and replaced with 100 μL of DMSO to solubilize the formazan crystals. Absorbance was then measured at 550 nm using a microplate reader (Multiskan FC, Thermo Fisher Scientific, Shanghai, China).

The cytocompatibility of the compound **5g** was assessed in the L929 cell line following the same experimental procedure. It was seeded at a cell density of 1 × 10^5^ cells/mL. The compound was tested at concentrations of 5, 15, 30, 60, and 100 μM, followed by 24 and 72 h incubation at 37 °C in a 5% CO_2_ atmosphere.

Cell viability was calculated as the percentage of tetrazolium salt reduced by viable cells in each sample, and the viability of the negative control (cells exposed to treatment-free medium) was taken as 100% cell viability. The experiments were performed at least three times, using three replicate samples for each compound concentration tested. The cytotoxicity of each compound was also expressed in terms of its IC_50_ (concentration causing 50% death of the cell population), calculated from concentration–response curves. Finally, the degree of selectivity of the compound **5g** against tumor cell lines is expressed by the selectivity index (SI), which was calculated using the following equation

$$selectivity\;index\;\left(SI\right)=\left[\frac{{IC}_{50}\;in\;nontumor\;cell\;line}{{IC}_{50}\;in\;tumor\;cell\;line}\right]$$

#### 2.6.3 Statistical Analysis

Statistical analyses were performed using two-way ANOVA followed by Bonferroni’s multiple comparisons test. Results were expressed as mean ± standard error of the mean (SEM) of three independent experiments. Differences were considered statistically significant when *p* < 0.05. Statistical analyses, graphical representations, and IC_50_ calculations were performed using GraphPad Prism version 8.0 for Windows (GraphPad Software, La Jolla, California, USA).

## 3 Results and Discussion

### 3.1 Synthesis of AZT Derivatives

Compounds 5′-arylchalcogenyl-(3-*N*-4,6-dichloro-1,3,5-triazin-2-yl)-thymidine (**5a**–**5k**) were synthesized in two steps. Initially, the synthesis of the precursors 5′-arylchalcogeno-3-aminothymidine (**3a**–**3k**) derived from AZT was performed according to the methodology previously developed by our research group^11,16^ (Scheme 1). As described, a small library of sulfides, selenides, and tellurides derivatives **3** containing electron-withdrawing and -donating groups attached at the para position of the aromatic ring was prepared.

**Scheme 1:**
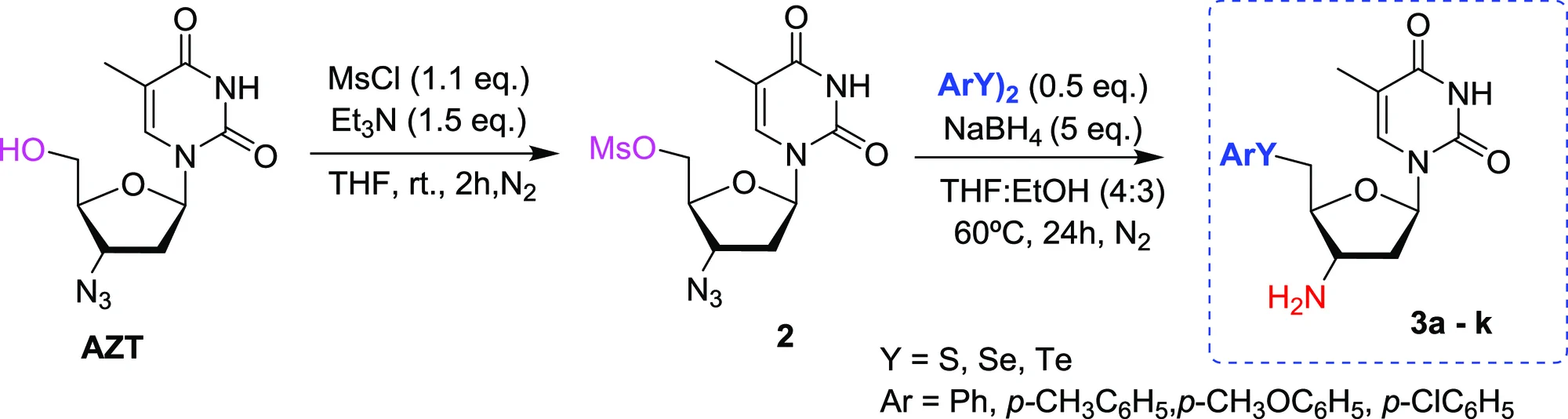
General Synthesis of 5′-Arylchalcogeno-3-aminothymidine (**3a**–**3k**)

Subsequently, with the respective amines **3** in hand, studies were initiated for the preparation of the derivatives (**5a**–**5k**), using compound **3a** as a representative nucleoside and the commercially available 2,4,6-trichloro-1,3,5-triazine **4** (Table 1).

**Table 1:**
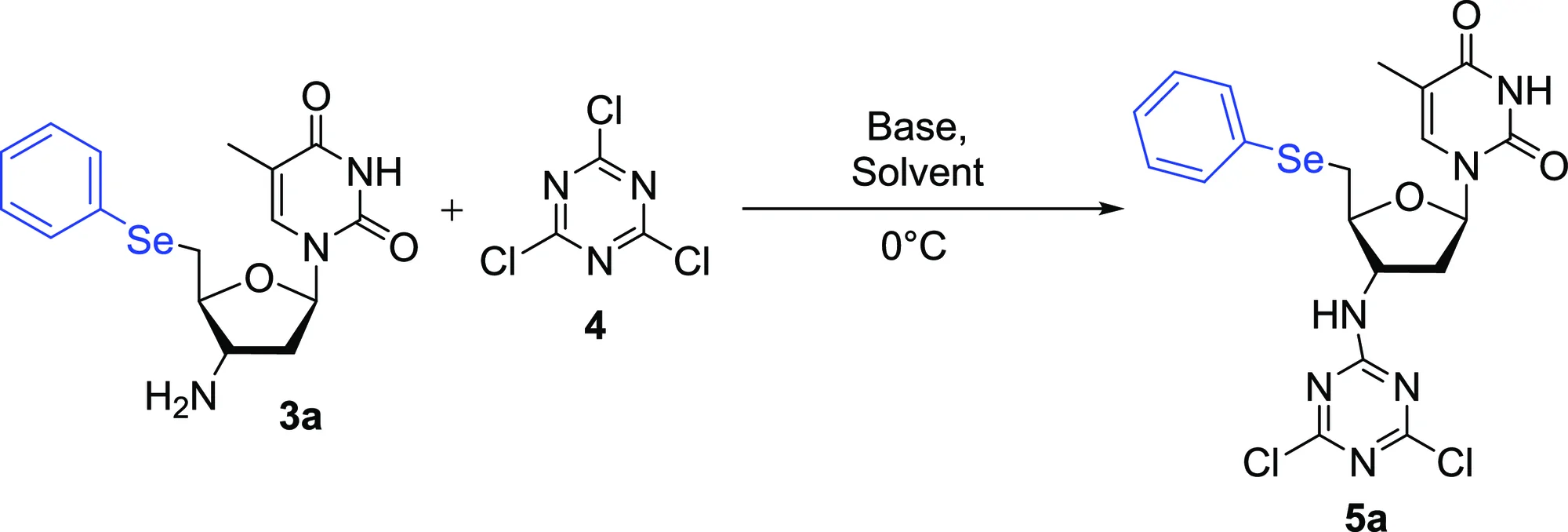
Optimization of Reaction Conditions

entry	time (h)	solvent	base (1 equiv)	yield^a^ (%)
1	1	acetone	Na_2_CO_3_	56
2	1	acetone	DIPEA	90
3	1	acetone	C_5_H_5_N	47
4	1	acetone	Et_3_N	75
5	1	CH_2_Cl_2_	DIPEA	60
6	1	CH_3_CN	DIPEA	85
7	1	THF	DIPEA	87
8	2	acetone	DIPEA	92
9	0.5	acetone	DIPEA	75

a Yields were determined after purification by a chromatographic column.

Due to the chemical characteristics of heterocycle **4**, which contains three electrophilic centers, regioselectivity control is a key point in obtaining monosubstituted products. In general, the degree of substitution in reactions involving this heterocycle **4** is controlled by variables such as temperature and substrate stoichiometry.^36^ Additionally, it has been observed that the first chlorine substitution of the heterocycle must occur at temperatures ≤0 °C, regardless of the nucleophile, due to the high reactivity of the ring. Meanwhile, the second substitution may occur either at room temperature or at ≤0 °C, depending on the substrate. After the electronic deficiency of the ring is reduced, additional energy in the form of heat becomes necessary for the third chlorine substitution to occur.

To optimize the reaction conditions, initial studies were carried out using compound **3a** (0.5 mmol) as nucleoside and 1,3,5-triazine-2,4,6-trichloro **4** (0.5 mmol). After 1 h of reaction at 0 °C, using acetone as the solvent and Na_2_CO_3_ as the base (1 equiv), compound **5a** was obtained in 56% yield (Table 1, **entry 1**). The efficiency of different bases was also evaluated. Pyridine and Et_3_N afforded products in yields of 45 and 75%, respectively (Table 1, **entries 3** and **4**). DIPEA (Table 1, **entry 2**) proved to be the most efficient base for the reaction, affording the product in 90% yield. Furthermore, the influence of the solvent was evaluated. Acetone, CH_2_Cl_2_, THF, and acetonitrile were tested; however, no significant variation in yield was observed compared to acetone (Table 1, **entries 2** and **5**–**7**, respectively). The reaction time was also studied. The highest yield was obtained within 1 h of reaction (Table 1, **entry 2**). Extending the reaction time to 2 h did not significantly affect the yield, whereas reducing it to 30 min led to a decrease in yield (Table 1, **entries 8** and **9**, respectively).

After defining the optimal reaction conditions, a variety of compounds were synthesized using different arylchalcogen-amines (**3a**–**3k**) via an SNAr reaction with heterocycle **4**. The products were obtained in yields ranging from 70 to 90% (Scheme 2).

**Scheme 2:**
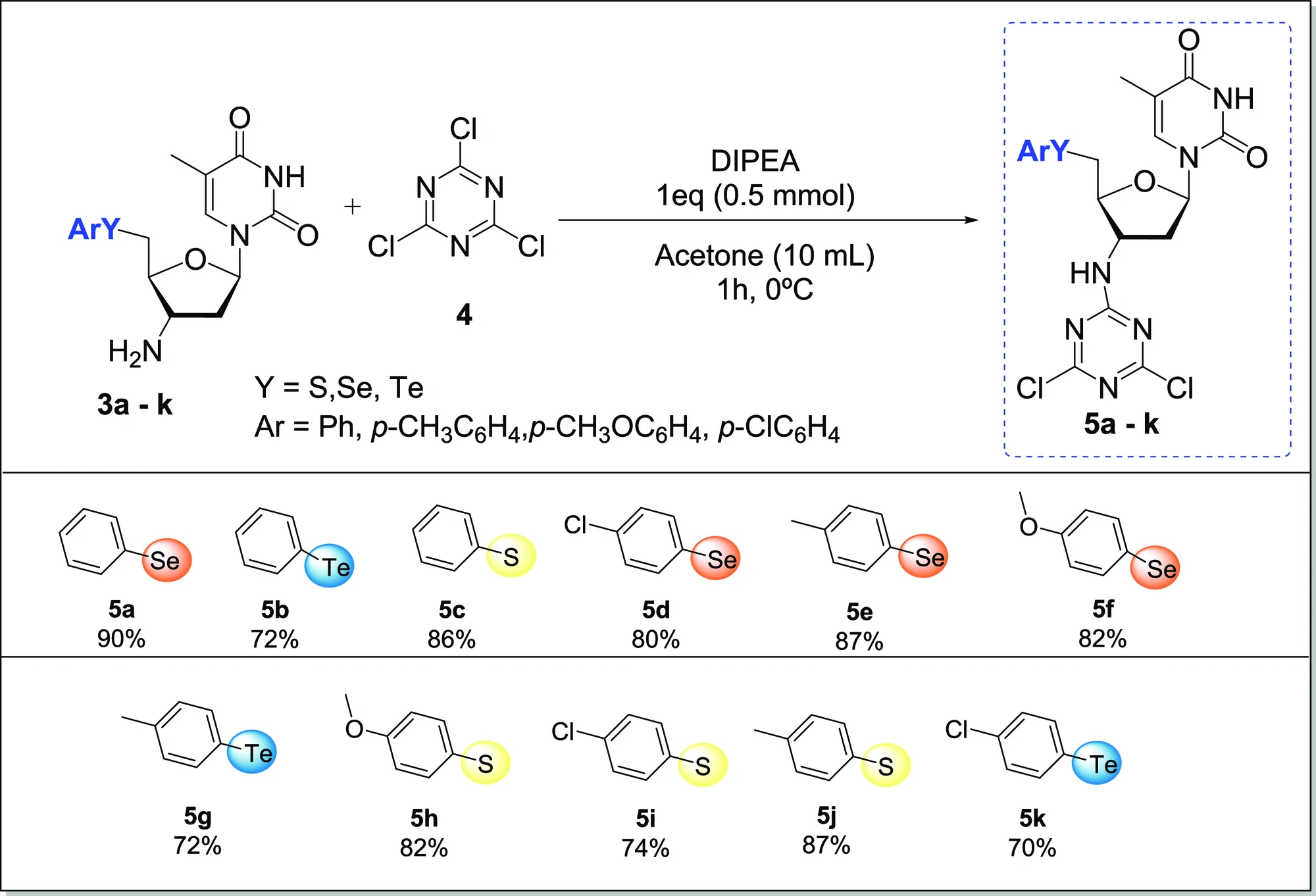
Synthesis and Library of 5′-Arylchalcogenyl-(3-*N*-4,6-dichloro-1,3,5-triazin-2-yl)-thymidine

In general, this methodology demonstrated good tolerance for substitutions with different zidovudine derivatives, **3**. Neutral, donating, and withdrawing groups attached to the aryl ring of amines **3** afforded the respective compounds with the same efficiencies. Additionally, sulfur, selenium, and tellurium derivatives were obtained in yields ranging from 70 to 90%. Slightly higher yields were observed for compounds containing selenium and sulfur atoms when compared with their tellurium analogues.

The respective compounds were characterized using nuclear magnetic resonance (NMR) spectroscopy (^1^H and ^13^C) and high-resolution mass spectrometry (HRMS) analysis (see SI).

### 3.2 Molecular Docking Study

Aminodeoxythymidine-triazine conjugates obtained in this study share a series of structural similarities with a great number of already known molecules with antitumor activity. Notably, tubulin polymerization inhibitors podophyllotoxin,^37^ colchicine,^38^ and indibulin^39^ bear two or more closely spaced cyclic units and bind to a specific site at the β subunit. The binding of known inhibitors at the colchicine-binding site leads to conformational changes of the tubulin structure, destabilizing the association of tubulin dimers and, consequently, the polymeric structure of microtubules.^40,41^ Inhibition of α,β-tubulin dimer polymerization imparts the formation of microtubules, thus potentially interrupting cell division and tumor development.^40,41^ Considering the presence of three cyclic substituents connected by at least partially flexible C–N and C–S bonds in the tested compounds, tubulin polymerization inhibition was proposed as a mechanism to be investigated using a molecular docking approach. For the study, an α,β-tubulin dimer bound to a podophyllotoxin derivative at the colchicine-binding site, with the PDB code 5JCB,^42,43^ was chosen as the protein model, with the ligand-binding site selected for the docking procedure in the AutoDock Vina program.^34^ Initially, the crystallized ligand structure was redocked within its original colchicine-binding site for the validation of the employed parameters, consisting of a rigid protein and a flexible ligand model. Redocking gave a satisfactory binding mode with an RMSD value of 0.7037 Å (Figure 2). Molecular docking suggested the binding to β-tubulin as a reasonable mechanism of action for the antitumor activity of compounds **5a**–**5k**, with binding affinity values ranging from −8.8 (**5a**) to −9.6 (**5i**) kcal·mol^–1^ according to the AutoDock Vina energy ranking (Table 2). Models of tellurium-bearing compounds **5b**, **5g**, and **5k** were docked separately due to the absence of tellurium atom parameters in the molecular docking program (Table 3). For these three compounds, selenium parameters were applied as a surrogate for tellurium atom parameters, based on their chemical similarity, while conserving tellurium’s atomic size and bond angles.

**Figure 2:**
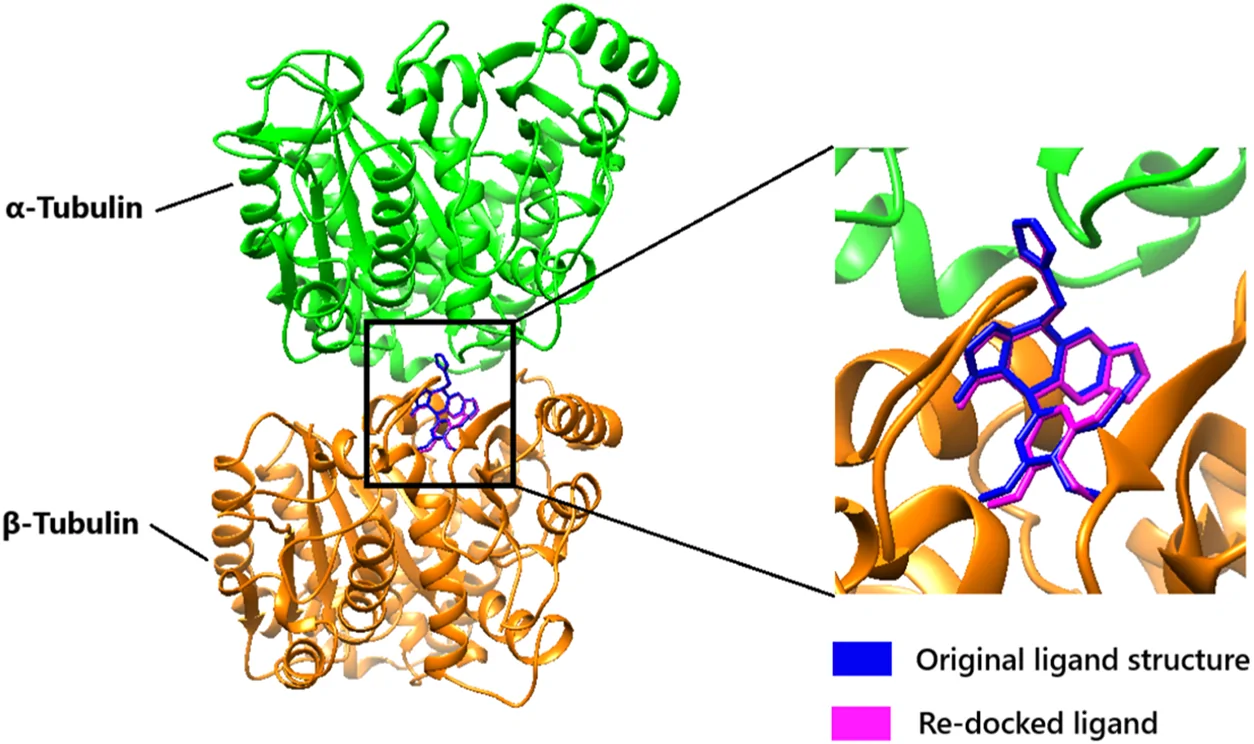
Graphical presentation of the αβ-tubulin dimer (PDB code 5JCB) with highlighted colchicine-binding site and redocked ligand structure for the validation of the employed molecular docking parameters.

**Table 2:** Binding Affinity Values of S- and Se-Bearing Compounds **5a**–**5j** According to the AutoDock Vina Program Ranking

compound	Y	R	binding affinity (kcal·mol^–1^)^a^
**5a**	Se	H	–8.8
**5c**	S	H	–8.9
**5d**	Se	Cl	–9.2
**5e**	Se	Me	–9.4
**5f**	Se	OMe	–9.3
**5h**	S	OMe	–9.4
**5i**	S	Cl	–9.6
**5j**	S	Me	–9.5
**redocking** ^b^			–11.1 (RMSD = 0.7037 Å)

a Rigid protein and flexible ligand models.

b The original ligand was extracted from the PDB file 5JCB.

**Table 3:** Binding Affinity Values of Te-Bearing Compounds **5­(b,g,k)** According to the AutoDock Vina Program Ranking Using Se Docking Parameters

compound	Y	R	binding affinity (kcal·mol^–1^)^a^
**5b**	Te	H	–10.6^b^
**5g**	Te	Me	–11.2^b^
**5k**	Te	Cl	–10.8^b^

a Rigid protein and flexible ligand model.

b Estimated binding affinity based on a tellurium-bearing structure model with selenium docking parameters as an approximation; not directly comparable to sulfur- and selenium-bearing examples.

According to the results, all examples fit within the binding site of β-tubulin predominantly through hydrophobic interactions. Examples **5a** and **5c**, bearing unsubstituted phenylchalcogenyl groups, were docked in approximately equivalent poses, with phenyl groups placed inside the binding pocket, engaging in amide-π-stacking with Val-315, and in highly hydrophobic π-alkyl interactions with nonpolar amino acid residues Val-181, Met-259, and Ala-316, and with the alkyl chain of Lys-352 (Figure 3). In these two examples, the thymine ring had only limited compatibility with the hydrophobic cavity, weakly interacting with Ala-250, Leu-248, and Leu-255. The triazine ring was located outside the β-chain, pointing to and interacting through hydrogen bonding with the nearby α-chain, namely, with polar residue Asn-101 amide hydrogen. The chloro substituents and π system of the same ring are also expected to interact in a hydrophobic mode with the Leu-248 alkyl chain of the β unit. Docking of examples **5d**–**5k**, bearing 4-substituted arylchalcogen groups, gave a different pose, which was roughly equivalent in all compounds of this subset, as well as in the case of tellurium-bearing compound **5b**. The hydrophobic arylchalcogen moieties in these docked examples lie in the cavity close to nonpolar amino acid residues Ala-250, Leu-255, Ala-316, and Ile-378 at the binding pocket, interacting through their π-electron cloud with nearby alkyl side chains and the sulfhydryl group of Cys-241 (Figure 4). The thymidine ring was placed close to Lys-254 (chain β) cationic alkylammonium and to Asn-101 (chain α) and Asn-249 (chain β) amide-bearing side chains, possibly engaging in hydrogen bonding between N–H groups and the ligand’s carbonyl oxygen. The central tetrahydrofuran ring and π-electron system of the thymine ring could also interact with the lipophilic Leu-248 (chain β) alkyl side chain. The other structural scaffold of the presented compounds, the aminodichlorotriazine moiety, is expected to interact in a more favorable way with its respective binding pocket in compounds **5b** and **5d**–**5k** compared to compounds **5a** and **5g**. The triazine-bound N–H group is expected to form a hydrogen bond with Thr-179 carbonyl (chain α), while the heterocyclic ring could interact with the nonpolar alkyl groups of Val-181 (chain α) and of Lys-352 (chain β) and with Met-259 thioether (chain β). In all compounds, the chalcogen atoms showed no strongly localized interactions with any close amino acid residue, with which they are expected to interact in a diffuse manner through van der Waals forces. The difference between the two main modes of interaction can be attributed to the larger size of 4-substituted arylchalcogenyl- and tellurium-bearing examples in comparison to unsubstituted sulfur and selenium analogues.

**Figure 3:**
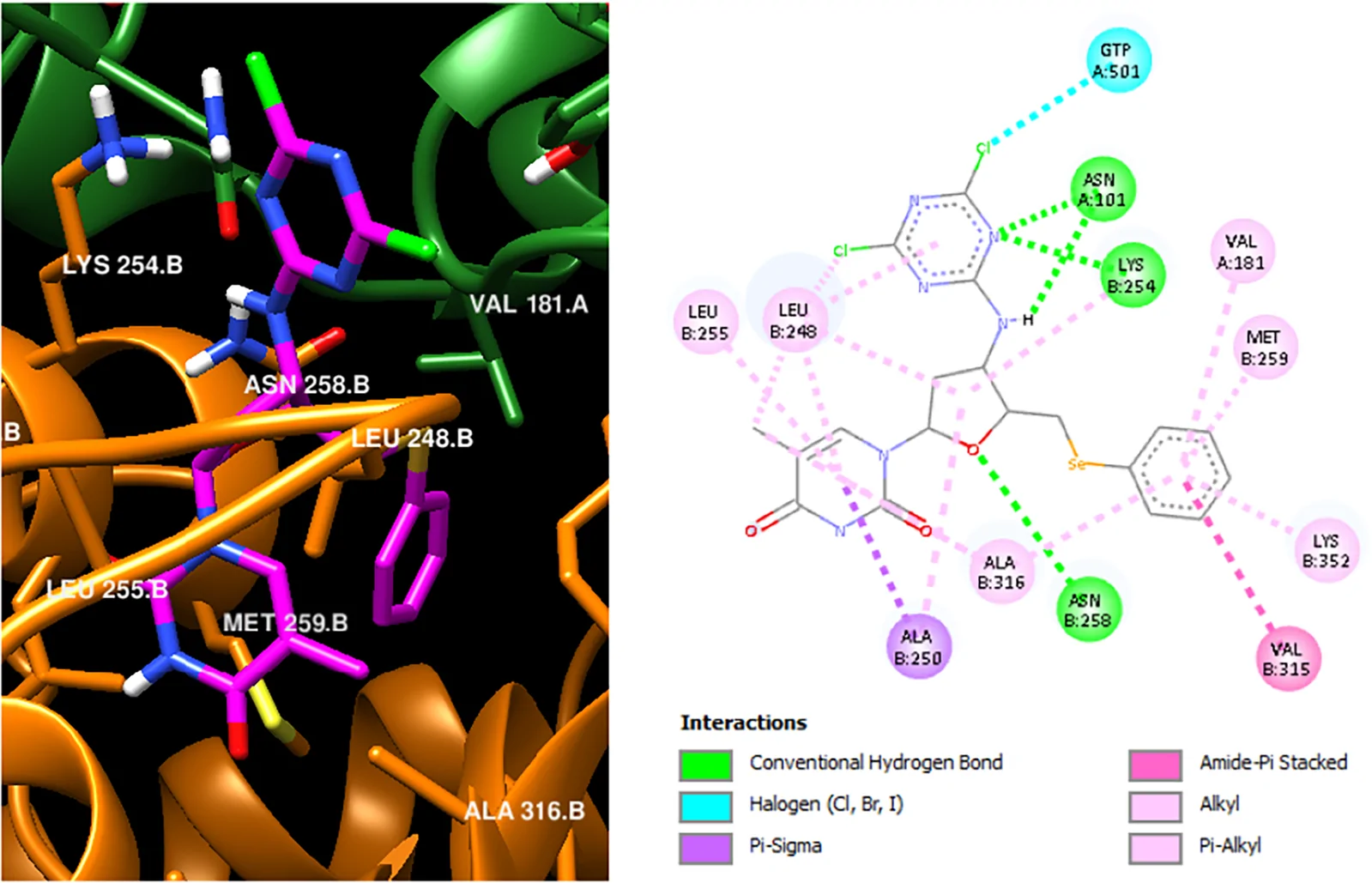
Three- and two-dimensional interaction diagrams of compound **5a** within the colchicine-binding site of the αβ-tubulin dimer.

**Figure 4:**
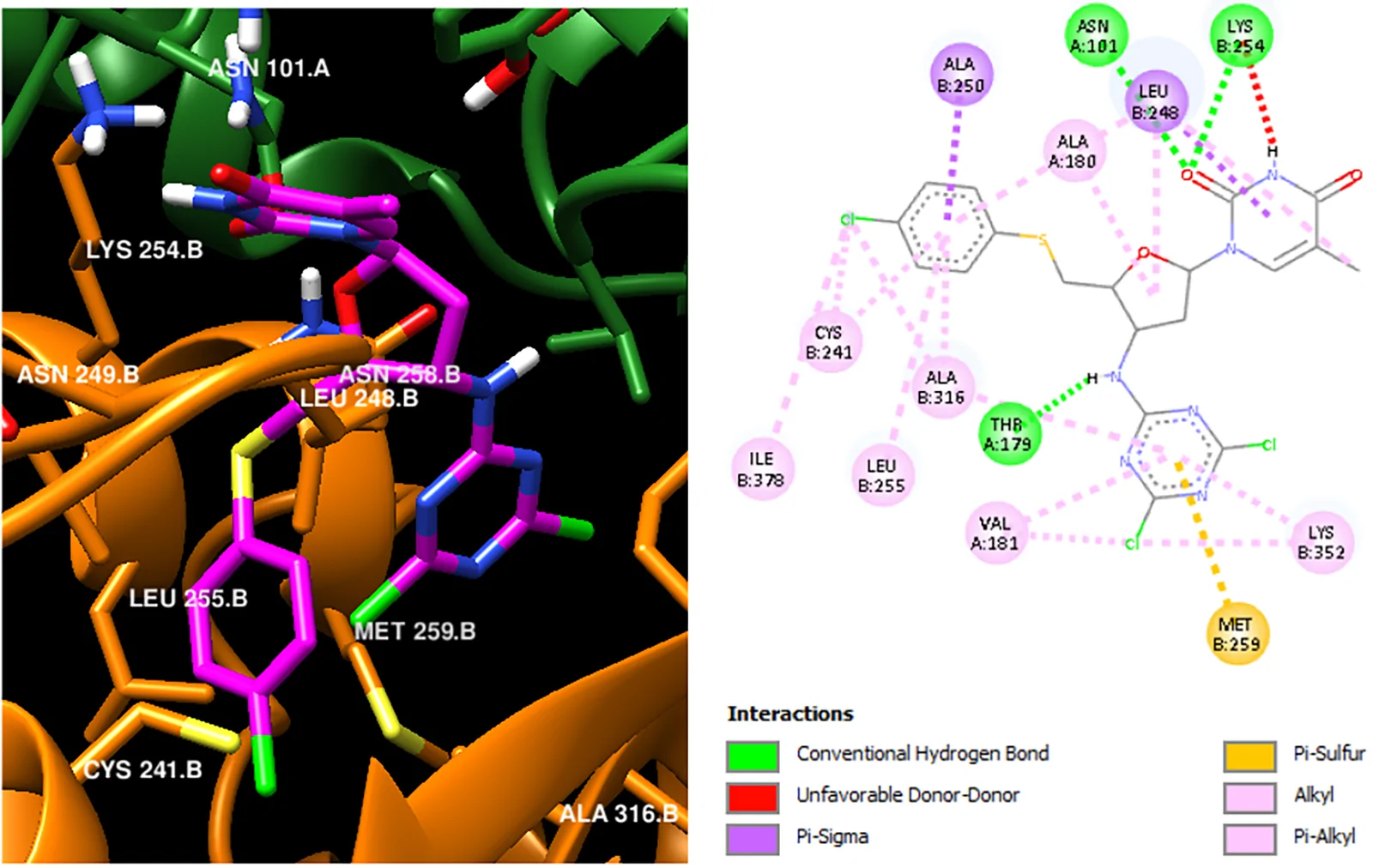
Three-dimensional diagrams of compound **5i** within the colchicine-binding site of the αβ-tubulin dimer.

It is worth noting that tellurium-bearing compounds were found to have an interaction pattern similar to those of other examples containing sulfur and selenium. However, the larger size of the tellurium atom in the chalcogen series may result in a more efficient space-filling at the hydrophobic binding site of the tubulin dimer. Moreover, redox-related reactivity in a biological medium, which is characteristic of organotellurium compounds, may also play an important role in the antitumor activity of the molecules.^44^

### 3.3 Antiproliferative Evaluation

#### 3.3.1 Antiproliferative Activity

The viability of HT-29 cells after 72 h of exposure to the organochalcogens was assessed using the MTT assay (Figure 5A). The Sulfur- and selenium-containing derivatives (**5a**, **5c**, **5d**, and **5f**) exhibited moderate cytotoxic effects against the sensitive tumor cells, with marked reduction in cell viability (>50% cytotoxicity) observed only at the highest tested concentration (100 μM). Conversely, tellurium-containing derivatives (**5b**, **5g**, and **5k**) significantly reduced cell viability in a concentration-dependent manner, indicating enhanced antitumor potential. The derivative **5k** displayed 93.7% cytotoxicity at the highest tested concentration (100 μM), while derivative **5g** induced cytotoxic effects ranging from 28.7 to 82.7%. Noteworthy is that derivative **5g** sustained antiproliferative activity across the tested concentration, with cell viability of 71.3% even at the lowest concentration (5 μM). The half-maximal inhibitory concentration (IC_50_) values for the tellurium-containing compounds were 41.4 μM (**5b**), 9.90 μM (**5g**), and 29.13 μM (**5k**), which were significantly lower than those of the other organocalchogens (61.69 to >100 μM) (Table 4).

**Figure 5:**
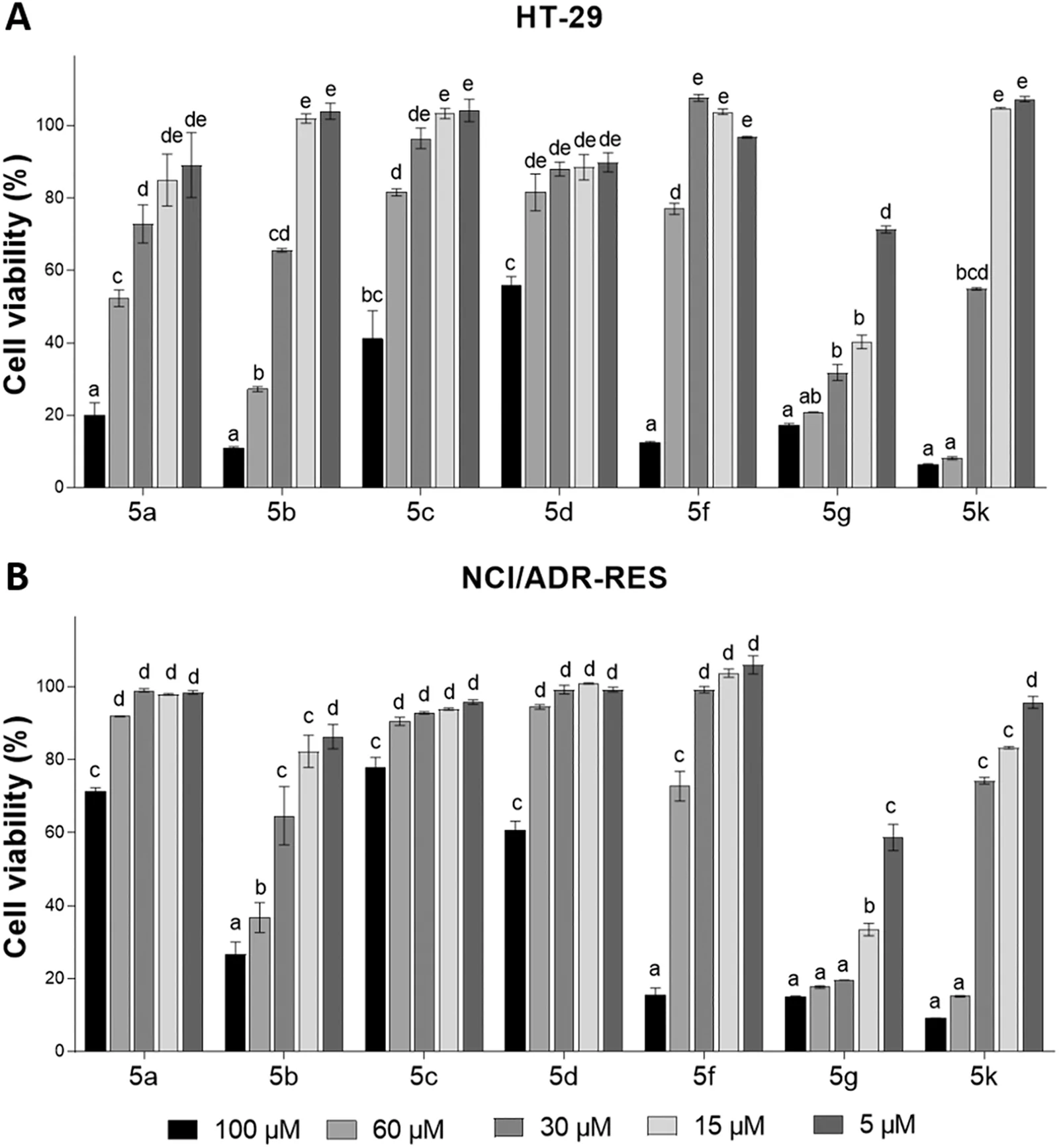
*In vitro* antiproliferative effects of the organochalcogen compounds measured by the MTT assay in (A) HT-29 and (B) NCI/ADR-RES cell lines. The compounds were tested at 100, 60, 30, 15, and 5 μM (*n* = 3). Results are expressed as mean ± SEM of cell viability, calculated as the percentage of tetrazolium salt reduced by viable cells in each sample. Statistical analyses were performed using two-way ANOVA followed by Bonferroni’s posthoc test. Different lowercase letters indicate statistically distinct groups (*p* < 0.05).

**Table 4:** Comparison of Cytotoxicity Measured as IC_50_ Values (μM) ± Standard Error (SE) for the Organochalcogen Compounds in HT-29 and NCI/ADR-RES Cell Lines

	IC _50_ ± SE (μM)	
organochalcogen	HT-29	NCI/ADR-RES
**5a**	84.44 ± 3.97	>100
**5b**	41.40 ± 6.92	41.66 ± 4.41
**5c**	73.63 ± 9.06	>100
**5d**	>100	>100
**5f**	61.69 ± 0.97	76.57 ± 1.84
**5g**	9.90 ± 3.78	12.18 ± 0.48
**5k**	29.13 ± 0.03	38.27 ± 0.13
**doxorubicin**	0.23 ± 0.10	64.66 ± 4.13

The effects of the organochalcogens **5a**, **5c**, and **5d** in resistant/MDR NCI/ADR-RES cells were less pronounced than in sensitive tumor cells, as cell viability remained relatively high across all tested concentrations, with no evident dose-dependent reduction in the number of viable cells (Figure 5B). These derivatives only moderately reduced the cell viability (∼25% cytotoxicity) at the highest tested concentration (100 μM). In contrast, derivative **5f** induced a slight to moderate decline in cell viability, which was able to reduce the viability at the two highest concentrations (72.7% and 15.6%) at 50 and 100 μM, respectively, achieving an IC_50_ of 76.57 μM. Notably, the tellurium-containing derivatives (**5b**, **5g**, and **5k**) exhibited the highest efficacy in the resistant/MDR cell line, demonstrating a pronounced concentration-dependent antiproliferative effect, similar to that observed in the sensitive tumor cells. Derivative **5b** induced cell cytotoxicity ranging from 13.7 to 73.4% (IC_50_ = 41.66 μM), while derivatives **5g** and **5k** were more active, with IC_50_ values of 12.18 and 38.27 μM, respectively. Notably, the tellurium derivatives exhibited lower IC_50_ values in this MDR cell line than the reference antitumor drug, doxorubicin. Conversely, the other less active organochalcogens (with S or Se) displayed IC_50_ values higher than 100 μM in this cell line (Table 4), indicating lower activity against MDR tumor cells. Overall, these results demonstrated the superior antiproliferative effects of tellurium-containing derivatives, highlighting their potential effectiveness for antitumor therapy, particularly in targeting MDR tumor cells.

#### 3.3.2 Cytotoxicity in Nontumor Cell Line

The cytotoxicity of the most active tellurium-containing derivative **5g** was evaluated in a nontumor cell line, L929. The compound exhibited high viability across all tested concentrations after 24 h of incubation, with values exceeding 90%, indicating good biocompatibility (Figure 6A). After 72 h of incubation, more pronounced cytotoxic effects were observed at the highest tested concentrations (30, 60, and 100 μM), indicating a time-dependent increase in toxicity (Figure 6B).

**Figure 6:**
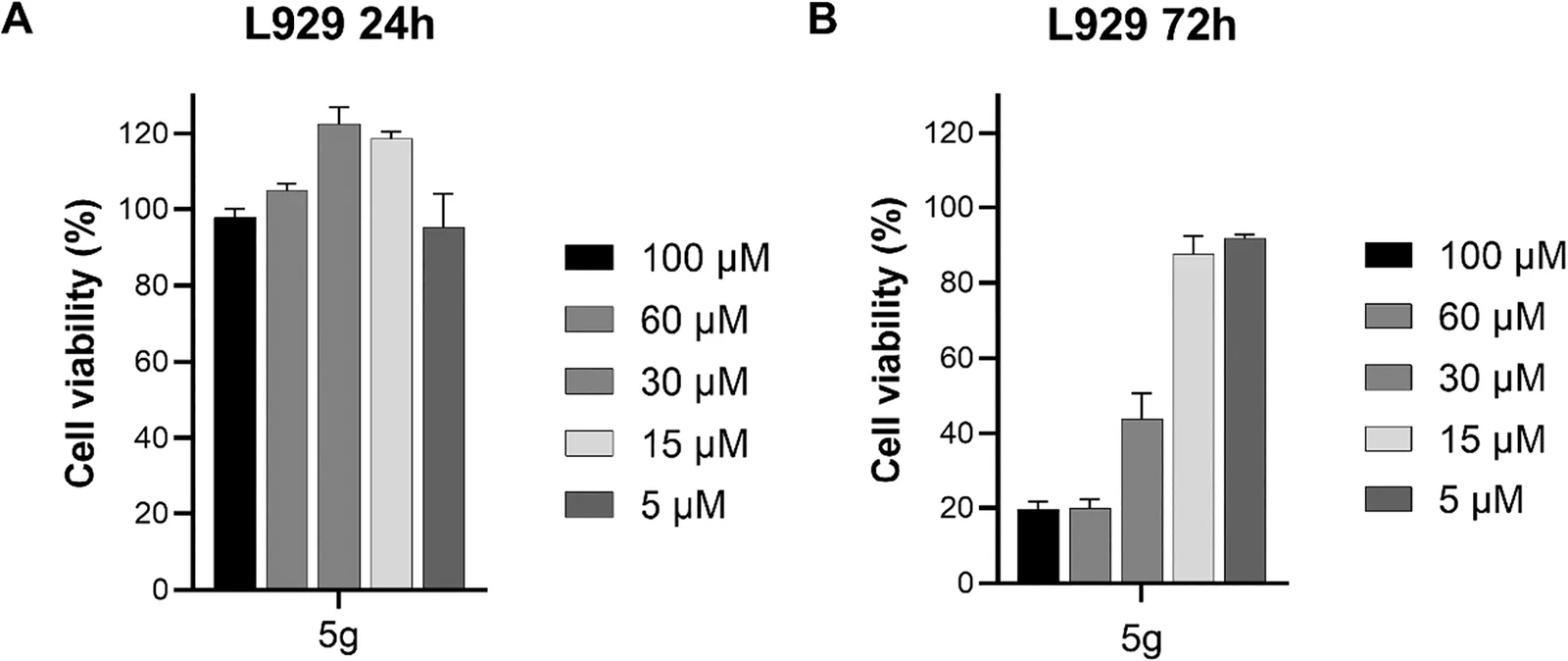
*In vitro* cytocompatibility of the organochalcogen **5g** measured by the MTT assay in nontumor L929 cell line after (A) 24 and (B) 72 h incubation. The compounds were tested at 100, 60, 30, 15, and 5 μM (*n* = 3). Results are expressed as mean ± SEM of cell viability, calculated as the percentage of tetrazolium salt reduced by viable cells in each sample.

The enhanced cytotoxicity was also reflected in the corresponding IC_50_ values (Table 5). The selectivity index (SI), defined as the ratio between the IC_50_ of the compound in nontumor cells and that in tumor cells, is greater than 2.0 in both tumor cell lines, indicating a selective cytotoxicity of the tellurium-containing derivative. The obtained SI values evidence the differential activity of compound **5g**, where values lower than 2.0 indicate a general toxicity of the tested compound.^45^ Therefore, the SI values highlight a favorable selectivity profile for compound **5g** against tumor cell lines.

**Table 5:** Cytotoxicity (IC_50_ ± SE, μM) of Organochalcogen **5g** in Nontumor L929 Cell Line after 24 and 72 h of Incubation, and Its Selectivity Index (SI) Relative to HT-29 and NCI/ADR-RES Tumor Cell Lines

IC_50_ ± SE (μM)	
organochalcogen **5g**	L929
24 h	>100
72 h	26.72 ± 1.52

selectivity index (SI)	organochalcogen **5g**
HT-29/72 h	2.70
NCI/ADR-RES/72 h	2.19

### 3.4 Antioxidant Evaluation

#### 3.4.1 Lipid Peroxidation by TBARS Assay

The ability of the compounds to inhibit lipid peroxidation in phosphatidylcholine samples was evaluated using the TBARS assay (Figures 7 and 8). Phosphatidylcholine is a major phospholipid component of the plasma membrane.^46^ Iron sulfate (FeSO_4_) was used as a pro-oxidant agent due to its ability to promote the Fenton reaction and induce lipid peroxidation.^46,47^ At 200 μM, the sulfur- and selenium-containing compounds (**5a**, **5c**, **5d**, and **5f)** did not significantly affect TBARS formation compared with the positive control. In contrast, the tellurium-containing compound **5b** significantly reduced iron-induced lipid peroxidation. A concentration–response analysis showed that compound **5b** retained protective activity at 10 and 20 μM.

**Figure 7:**
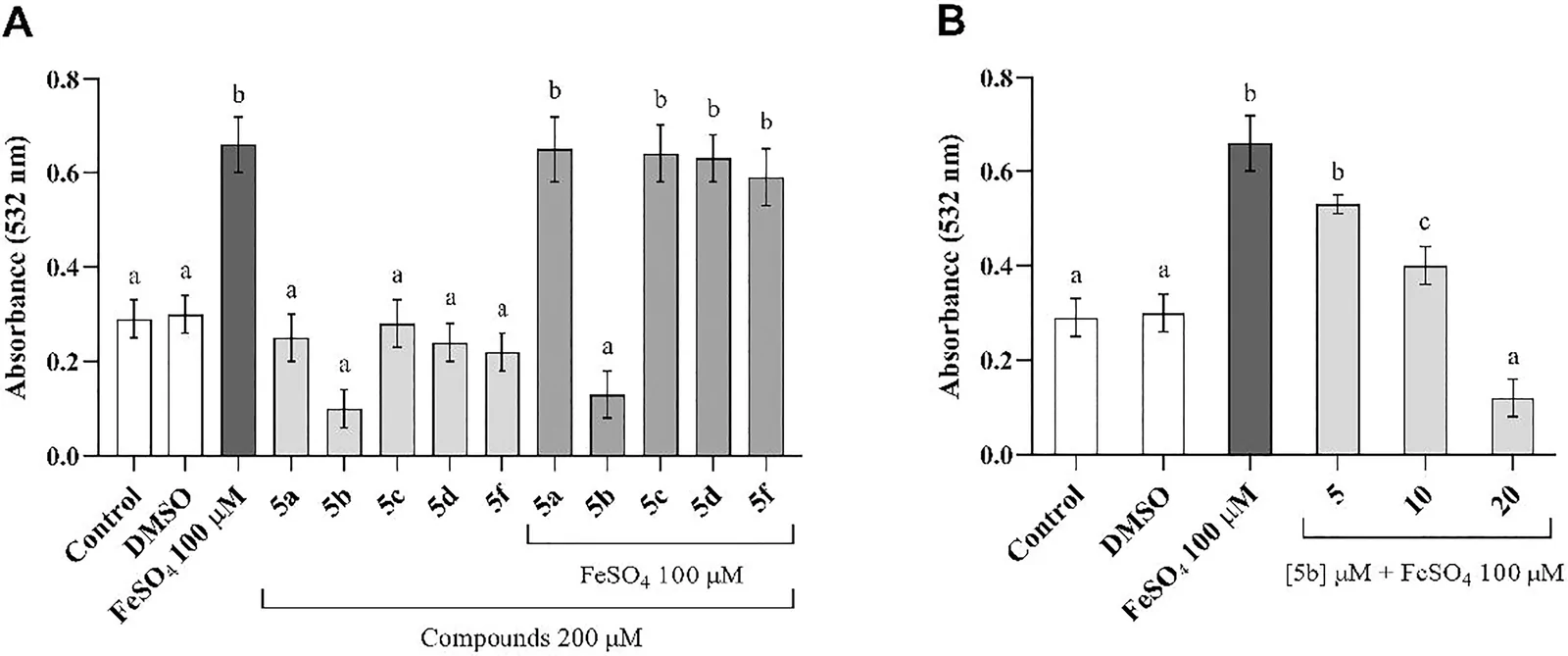
Lipid peroxidation in phosphatidylcholine exposed to compounds **5a**–**5f**. Lipid peroxidation in phosphatidylcholine exposed to compounds **5a**–**5f**. TBARS levels were measured in phosphatidylcholine samples (7.5 mg/mL) incubated with or without compounds (200 μM) in the presence of 100 μM FeSO_4_. (A) Effects of compounds at 200 μM expressed as absorbance at 532 nm. (B) Concentration–response curve (5, 10, and 20 μM) for compound **5b**. Data are expressed as mean ± SEM from three independent experiments. *p ≤ 0.05 vs positive control (H_2_O + FeSO_4_). Different lowercase letters indicate statistically distinct groups (one-way ANOVA followed by Tukey’s test).

**Figure 8:**
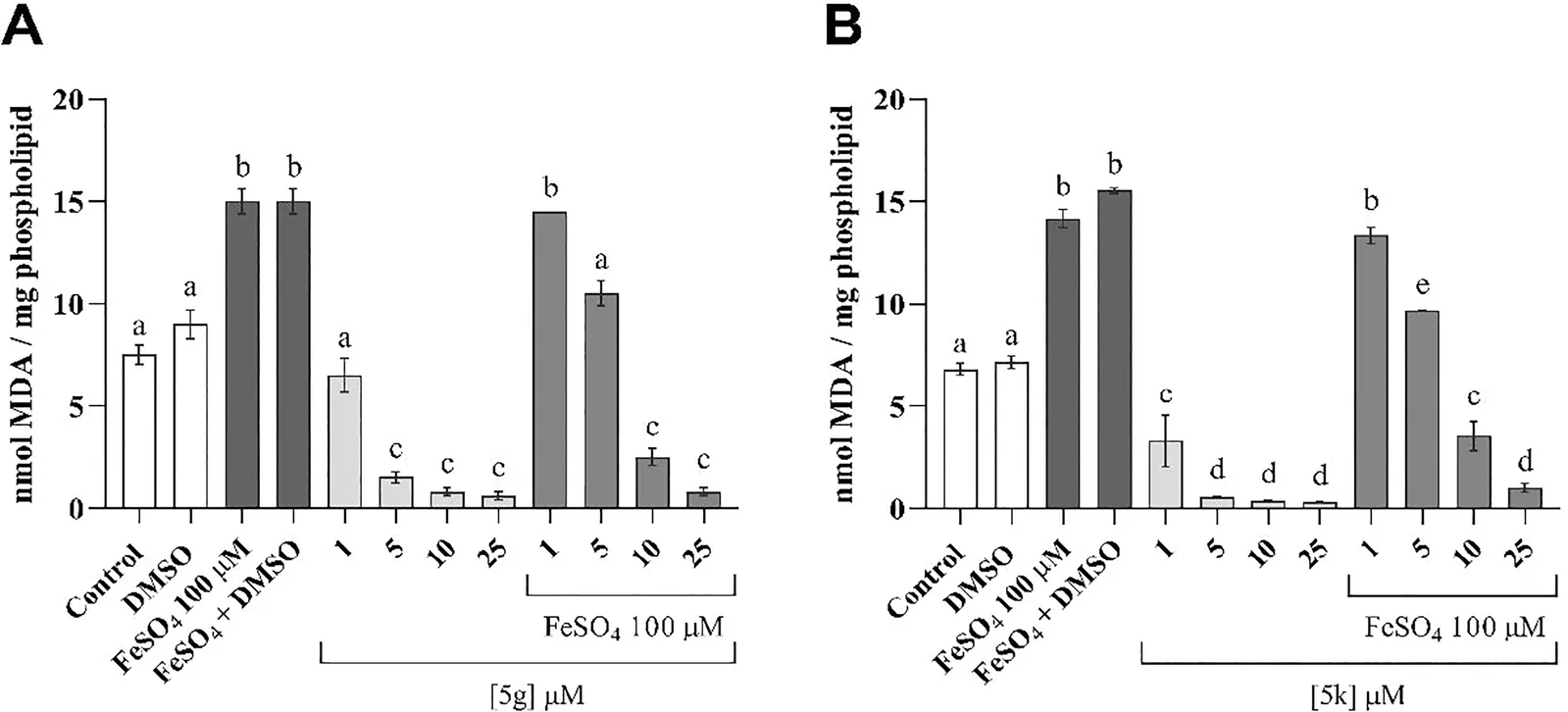
Lipid peroxidation of phosphatidylcholine exposed to compounds **5g** and **5k**. Lipid peroxidation in phosphatidylcholine exposed to compounds **5g** and **5k**. TBARS levels were measured in phosphatidylcholine samples (4.28 mg/mL) incubated with or without compound **5g** (A) or **5k** (B) at 1, 5, 10, and 25 μM in the presence of 100 μM FeSO_4_. Results are expressed as mean ± SEM (nmol MDA/mg phospholipid) from three independent experiments (*n* = 3). Different letters indicate statistically different groups (one-way ANOVA followed by Tukey’s posthoc test).

Although organotellurium compounds may raise toxicity concerns, they have been reported to exhibit *in vitro* antioxidant activity.^48,49^ Based on the protective effect observed for compound **5b**, two additional tellurium-derived compounds (**5g** and **5k**) were selected for further antioxidant evaluation. As shown in Scheme 2, compound **5g** contains a methyl substituent in the para position relative to the tellurium atom, whereas compound **5k** contains a chlorine atom in the same position. Both derivatives partially prevented iron-induced lipid peroxidation at 5 μM (Figure 7). Complete prevention was observed at 10 and 25 μM for both compounds. In addition, compound **5g** reduced basal TBARS levels at all tested concentrations (5–25 μM) (Figure 8A). Compound **5k** exhibited an even stronger effect, decreasing basal lipid peroxidation across all concentrations (Figure 8B).

#### 3.4.2 Thiol-peroxidase-like Activity

The classical method for measuring thiol-peroxidase activity involves the oxidation of thiophenol (PhSH) by hydrogen peroxide (H_2_O_2_), with spectrophotometric monitoring of diphenyl disulfide (PhSSPh) formation.^49,50^ The compounds **5a**, **5c**, **5d**, and **5f** did not display measurable thiol-peroxidase-like activity at 200 μM (Figure 9A). In contrast, compound **5b** exhibited significant GPx-like activity compared with the controls (H_2_O and DMSO), even surpassing the positive control (PhSe)_2_ (Figure 9A). A concentration–response analysis demonstrated that compound **5b** retained significant activity at 10, 20, and 50 μM (Figure 8B). Additional experiments indicated that most antitumor active compound **5g** also exhibited GPx-like activity at concentrations ≥10 μM (Figure 10).

**Figure 9:**
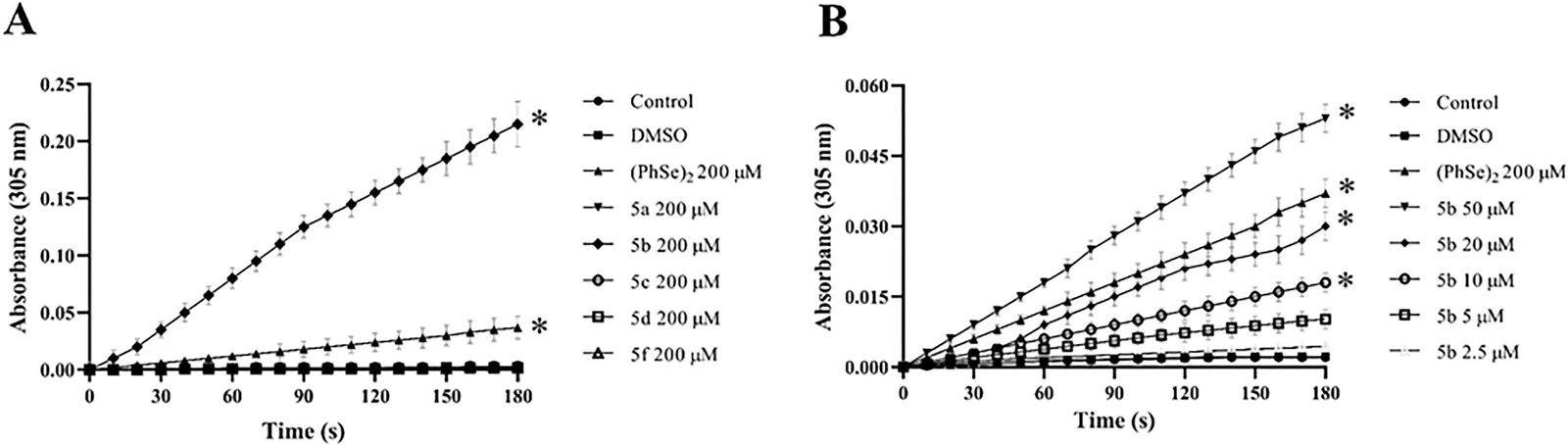
Thiol-peroxidase-like activities of compounds **5a**–**5f**. (A) Compounds were initially tested at 200 μM. Controls consisted of distilled water or DMSO (*n* = 4). (B) Concentration–response curve for compound **5b** (50, 20, 10, 5, and 2.5 μM; *n* = 3). *p ≤ 0.05 vs control by two-way ANOVA followed by Tukey’s multiple comparison test. Error bars were omitted for clarity and were <5% of the respective means.

**Figure 10:**
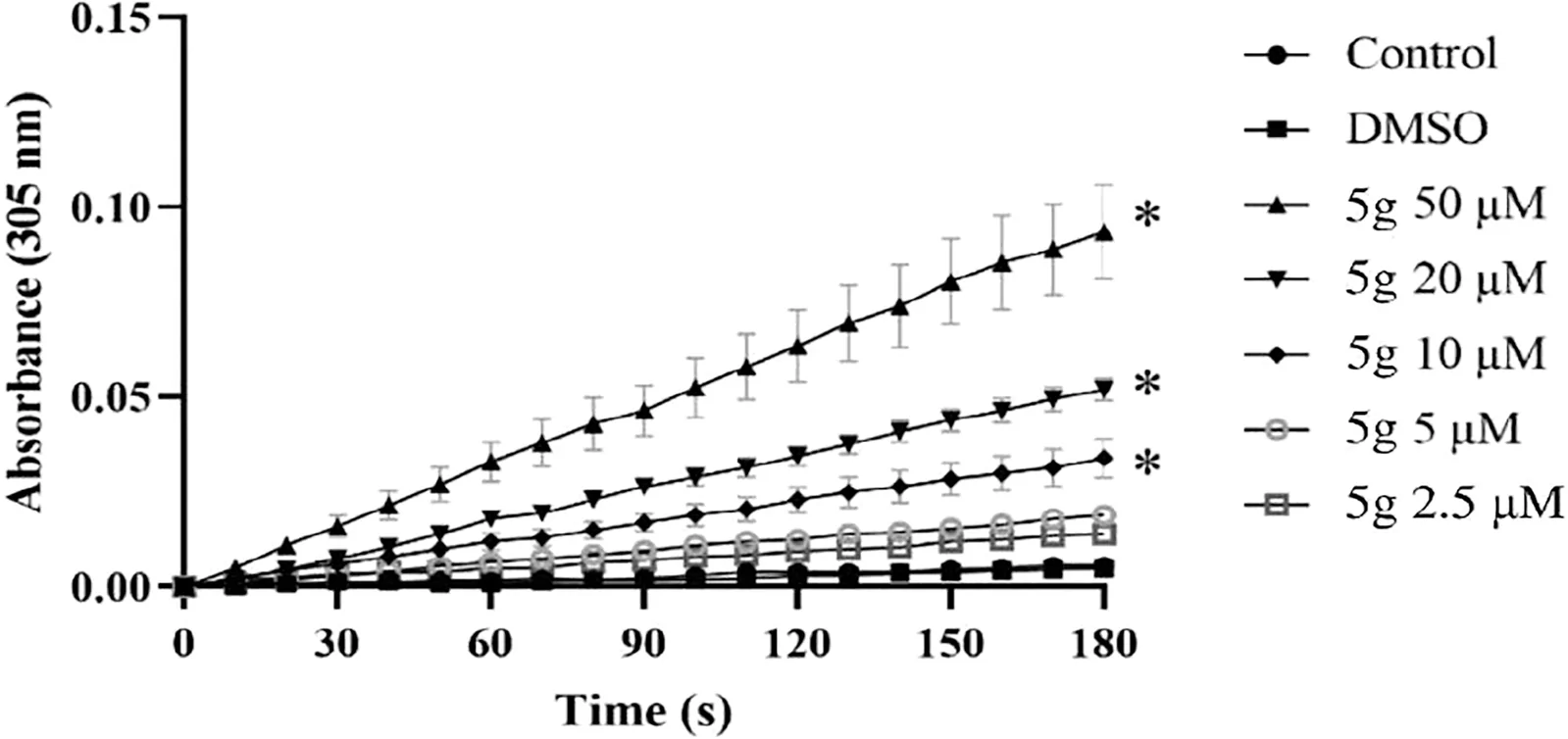
Thiol-peroxidase-like activity of compound **5g**. The compound was tested at 50, 20, 10, 5, and 2.5 μM (*n* = 3). Results are expressed as mean ± SEM of absorbance at 305 nm. *p ≤ 0.05 vs control by two-way ANOVA followed by Tukey’s multiple comparison test.

## 4 Conclusion

This study reports the synthesis and comprehensive biological evaluation of novel 5′-arylchalcogenyl-(3-*N*-4,6-dichloro-1,3,5-triazin-2-yl)-thymidine derivatives. The synthetic protocol enabled the preparation of a series of sulfide, selenide, and telluride analogues in yields ranging from 70 to 90%. Biological assays revealed a clear chalcogen-dependent effect on both antitumor and antioxidant activities. Although sulfur- and selenium-containing compounds exhibited moderate inhibition of tumor cell growth, they showed no significant activity in antioxidant assays. In contrast, tellurium derivatives demonstrated pronounced antitumor efficacy, including activity against multidrug-resistant cancer cell lines, as well as potent antioxidant properties. Molecular docking procedures revealed that the studied compounds can potentially bind to the colchicine-binding site on the tubulin dimer, mainly at β-tubulin. This suggests that the inhibition of tubulin polymerization may be a possible mode of action and identifies future biological studies as essential to experimentally validating the theoretical results. These findings highlight the critical role of the chalcogen atom in determining the multitarget biological activities of these thymidine derivatives.

## Supplementary Material



## Notes

^1^H and ^13^C NMR of the compound **5a** (Figure S1); ^1^H and ^13^C NMR of the compound **5b** (Figure S2); ^1^H and ^13^C NMR of the compound **5c** (Figure S3); ^1^H and ^13^C NMR of the compound **5d** (Figure S4); ^1^H and ^13^C NMR of the compound **5e** (Figure S5); ^1^H and ^13^C NMR of the compound **5f** (Figure S6); ^1^H and ^13^C NMR of the compound **5g** (Figure S7); ^1^H and ^13^C NMR of the compound **5h** (Figure S8); ^1^H and ^13^C NMR of the compound **5i** (Figure S9); ^1^H and ^13^C NMR of the compound **5j** (Figure S10); ^1^H and ^13^C NMR of the compound **5k** (Figure S11); HRMS of **5a** (Figure S12); HRMS of **5b** (Figure S13); HRMS of **5c** (Figure S14); HRMS of **5d** (Figure S15); HRMS of **5e** (Figure S16); HRMS of **5f** (Figure S17); HRMS of **5g** (Figure S18); HRMS of **5h** (Figure S19); HRMS of **5i** (Figure S20); HRMS of **5j** (Figure S21); two-dimensional interaction diagram of compound **5a** bound to the αβ-tubulin dimer (Figure S23); two-dimensional interaction diagram of compound **5b** bound to the αβ-tubulin dimer (Figure S24); two-dimensional interaction diagram of compound **5c** bound to the αβ-tubulin dimer (Figure S25); two-dimensional interaction diagram of compound **5d** bound to the αβ-tubulin dimer (Figure S26); two-dimensional interaction diagram of compound **5e** bound to the αβ-tubulin dimer (Figure S27); two-dimensional interaction diagram of compound **5f** bound to the αβ-tubulin dimer (Figure S28); two-dimensional interaction diagram of compound **5g** bound to the αβ-tubulin dimer (Figure S29); two-dimensional interaction diagram of compound **5h** bound to the αβ-tubulin dimer (Figure S30); two-dimensional interaction diagram of compound **5i** bound to the αβ-tubulin dimer (Figure S31); two-dimensional interaction diagram of compound **5j** bound to the αβ-tubulin dimer (Figure S32); two-dimensional interaction diagram of compound **5k** bound to the αβ-tubulin dimer (Figure S33) (PDF)
