# Supplementary material for: Synthesis and In Silico and In Vitro Antitumor Potential of Novel Organochalcogens Derived from Azidothymidine and 2,4,6-Trichloro-1,3,5-triazine
Source: ACS Omega. 2026 Oct 10;11(42):65276–89. doi: 10.1021/acsomega.6c07643 (PMC13717658; doi:10.1021/acsomega.6c07643)

Synthesis and *In Silico* and *In vitro* antitumor potential of novel organochalcogens derived from azidothymidine and 2,4,6-Trichloro-1,3,5-triazine

Nayra Salazar Rocha[a] , Sidne Rodrigues da Silva [a] , Brenda Bomm Carvalho[a], Marcelo Wendt,[a] Luciano Dornelles[a] , Luciana Filippin Cossetin[b], Bianca Costa Maia-do-Amaral[b], Daniele Rubert Nogueira-Librelotto[b], Raquel Mello da Rosa[a], Alessandro de Souza Prestes[c], João Cândido Pilar Mayer[d] Nilda Vargas Barbosa[c], João Batista Teixeira da Rocha[c], Oscar Endrigo Dorneles Rodrigues [a] *

[a] LabSelen-NanoBio – Department of Chemistry, Federal University of Santa Maria - Universidade Federal de Santa Maria, Av. Roraima, 1000, CEP: 97105-900, Santa Maria, RS, Brazil.

[b] Laboratory of *In Vitro* Pharmaceutical Testing and Assays, Department of Industrial Pharmacy, Federal University of Santa Maria, Av. Roraima, 1000, CEP:97105-900, Santa Maria, RS, Brazil.

[c] Department of Biochemistry and Molecular Biology, Federal University of Santa Maria, Santa Maria, Av. Roraima, 1000, CEP: 97105-900, Santa Maria, RS, Brazil.

[d] Laboratory of Molecular Catalysis, Institute of Chemistry, Federal University of Rio Grande do Sul, Av. Bento Gonçalves, 9500, CEP: 91509-900, Porto Alegre, RS, Brazil.

Supporting Information

**1. General Considerations**	**S1**
**2. NMR ^{13}C and H^1 charts**	**S2**
**3. HRMS charts**	**S12**
**4. Interaction diagrams of docked compounds**	**S15**

1. General Considerations

All Chemicals were of analytical grade and obtained from standard commercial suppliers and some reactions were run under an atmosphere of dry argon. Proton nuclear magnetic resonance spectra (^1H NMR) were obtained at 600 MHz in a Bruker Avance III HD NMR spectrometer. Spectra were recorded in $CDCl_3$ or DMSO-d_6 solutions. Chemical shifts are reported in ppm, referenced to the solvent peak of tetramethylsilane (TMS) as the external reference. Data are reported as follows: chemical shift (δ) expressed in ppm, multiplicity (br = broad, s = singlet, d = doublet, dd = doublet of doublets, ddd = doublet of doublet of doublets, dt = doublet of triplets, t = triplet, m = multiplet, q = quartet), and coupling constant (J) in Hertz and integrated intensity. Carbon-13 nuclear magnetic resonance (^{13}C NMR) spectra were obtained either at 100 MHz in na AVANCE III HD NMR spectrometer. Chemical shifts (δ) are reported in ppm, referenced to the solvents peak of $CDCl_3$ or DMSO-d_6. For visualization, TLC plates were either placed under ultraviolet light, or stained with iodine steam.

2. NMR ^{13}C and H^1 charts

Figure S1. ^{1}H and ^{13}C NMR of the compound **5a** in $CDCl_3$ at 600 MHz and 150 MHz respective

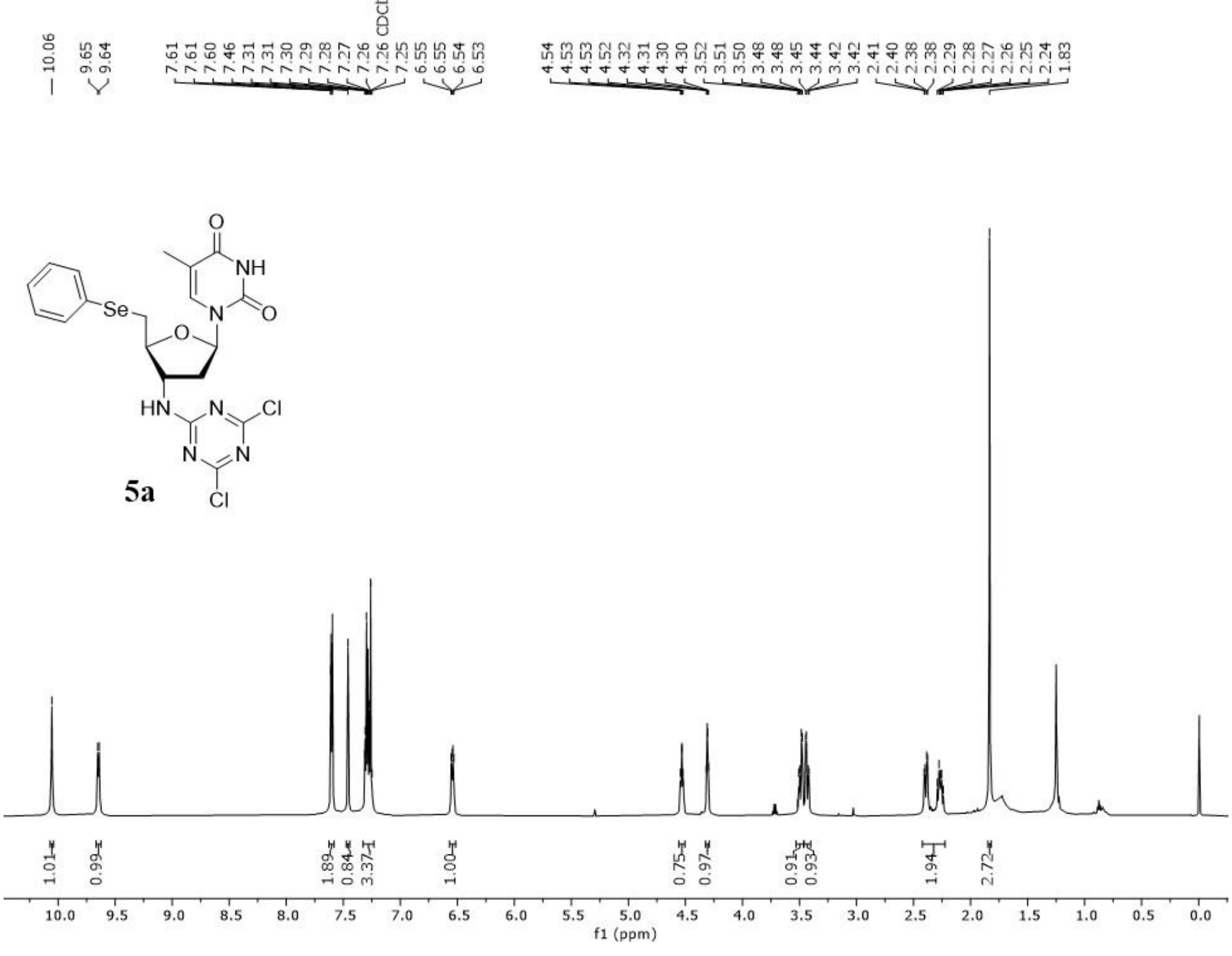

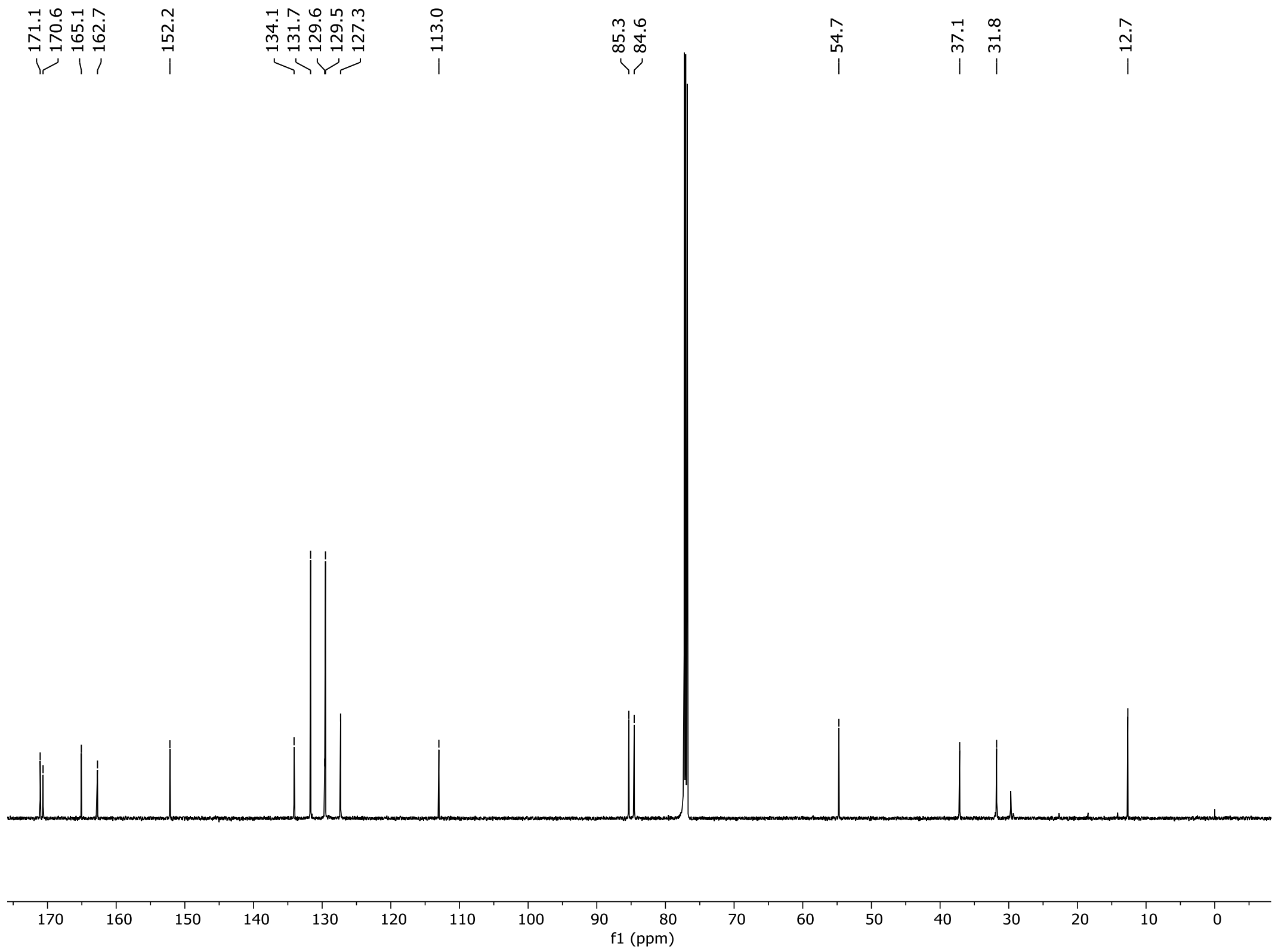

Figure S2. ^{1}H and ^{13}C NMR of the compound **5b** in $CDCl_3$ at 600 MHz and 150 MHz respectively.

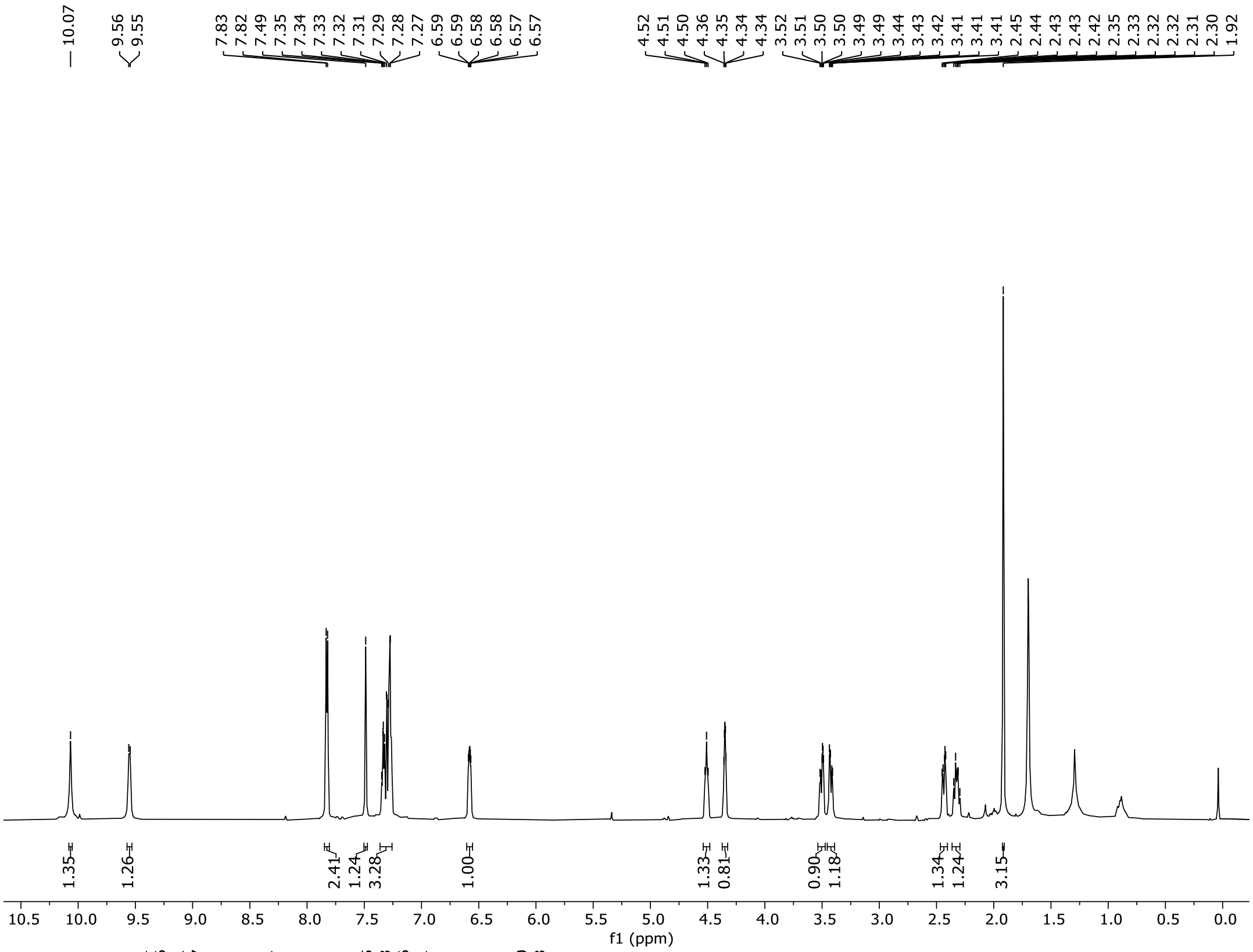

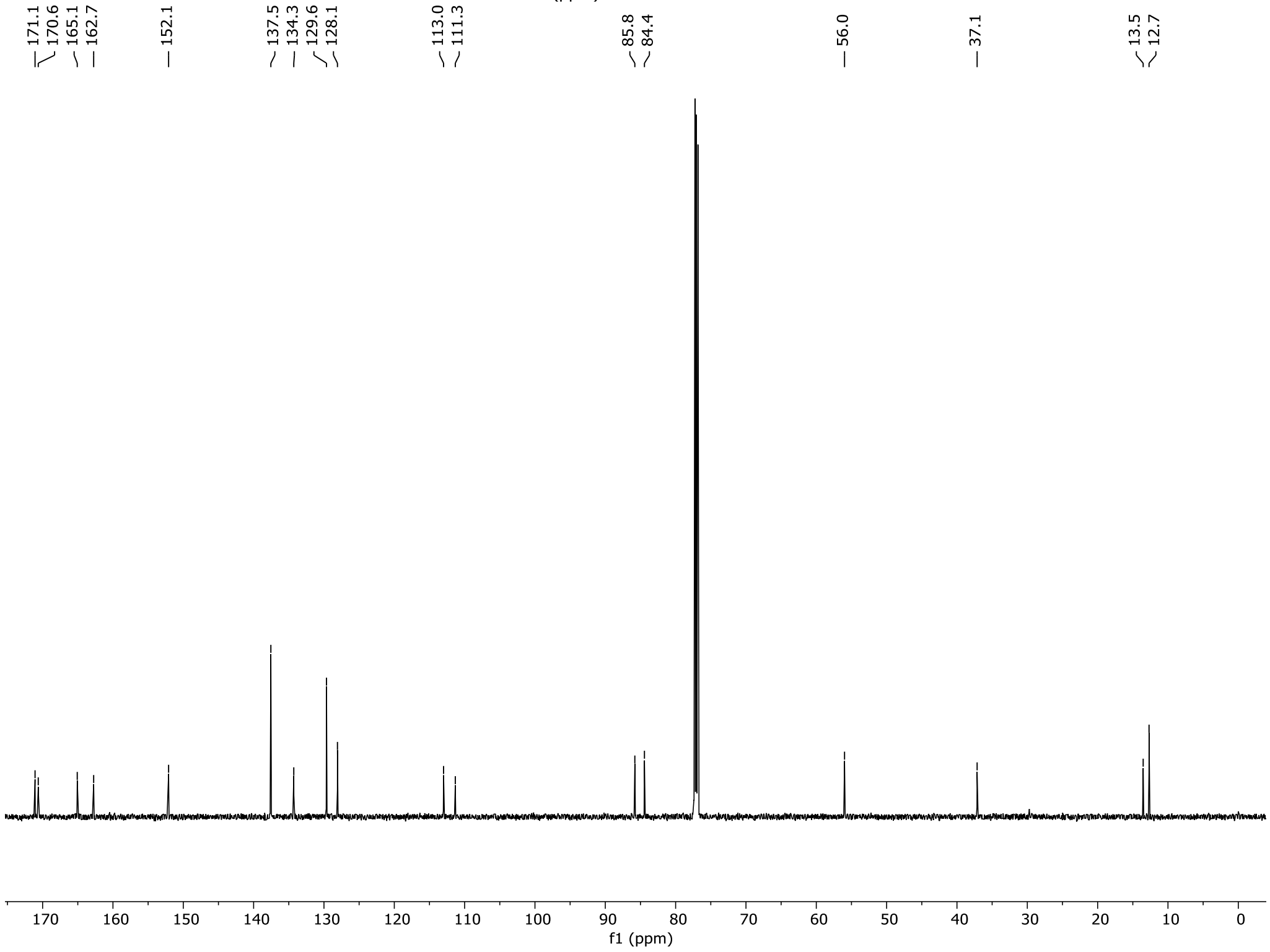

Figure S3. ^{1}H and ^{13}C NMR of the compound **5c** in $CDCl_3$ at 600 MHz and 150 MHz respectively

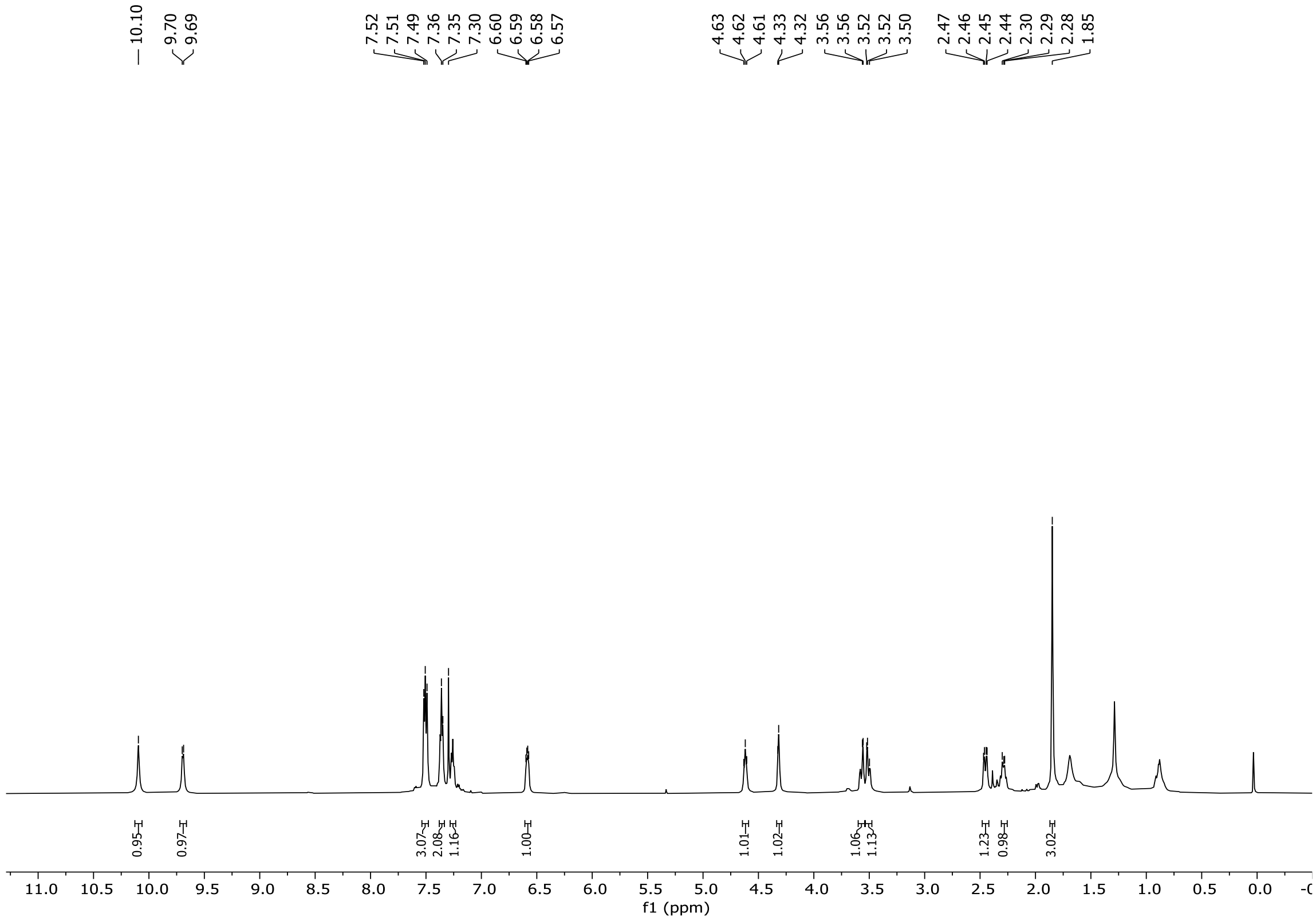

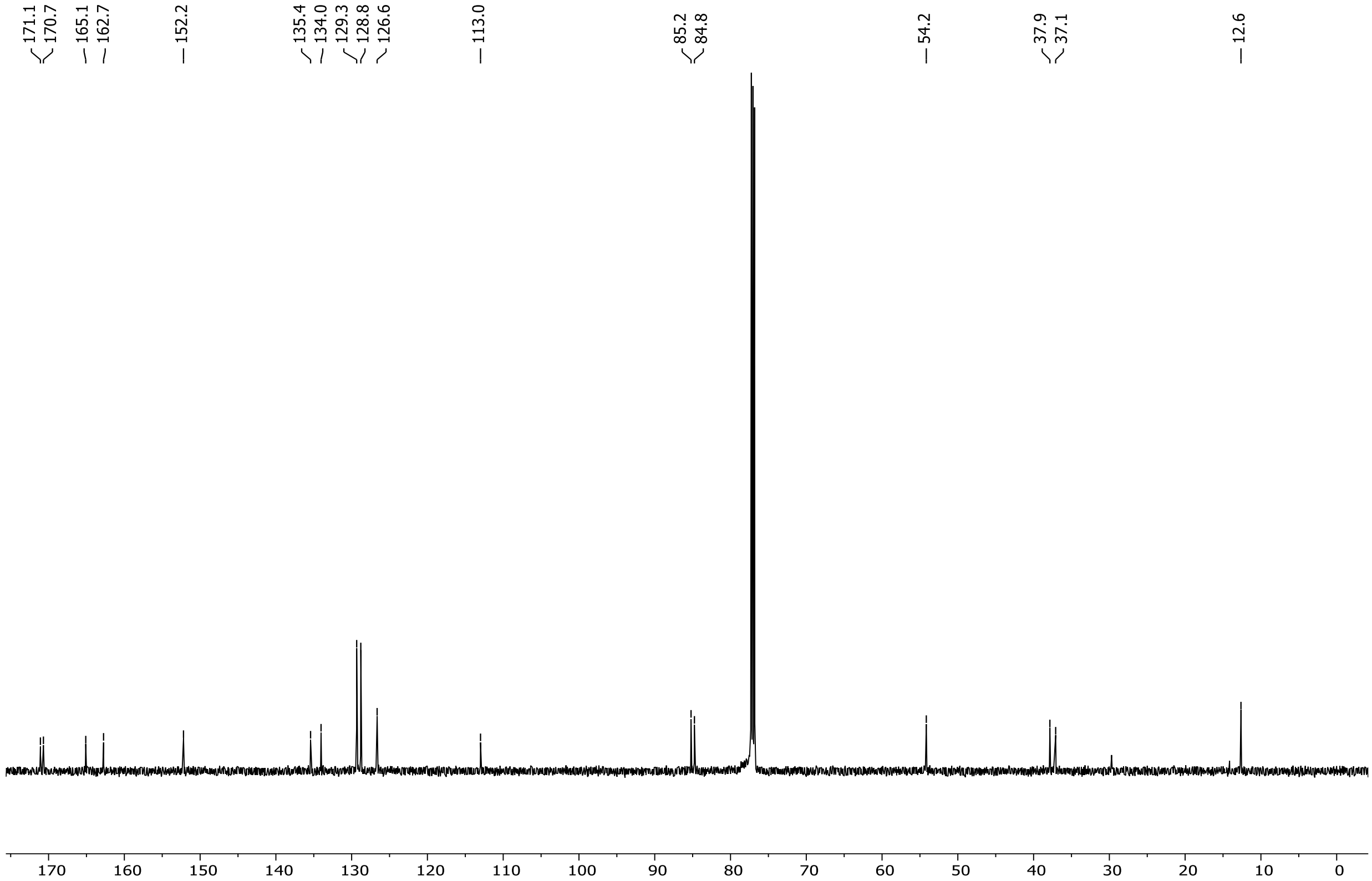

Figure S4. ^{1}H and ^{13}C NMR of the compound **5d** in $CDCl_3$ at 600 MHz and 150 MHz respectively

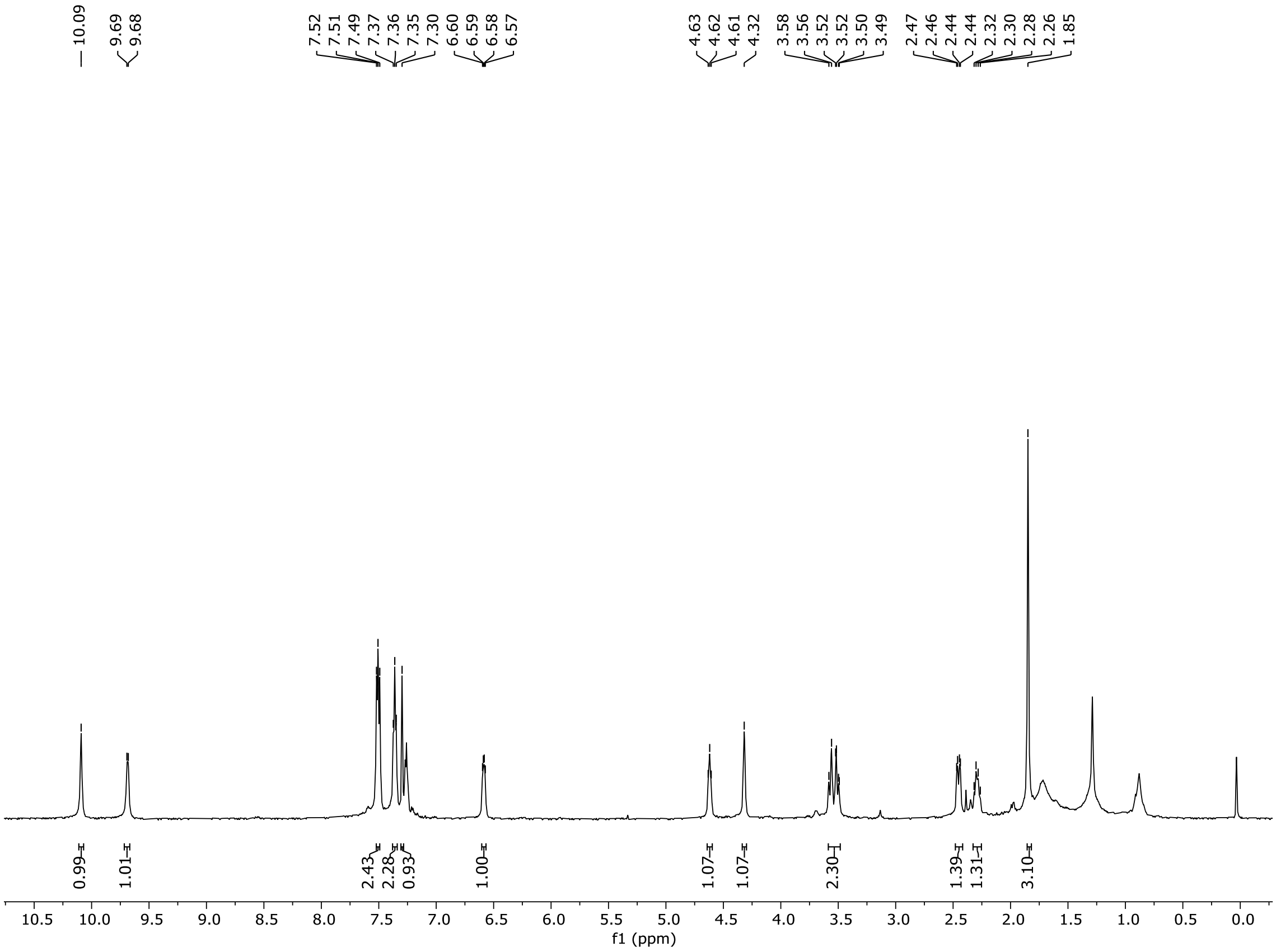

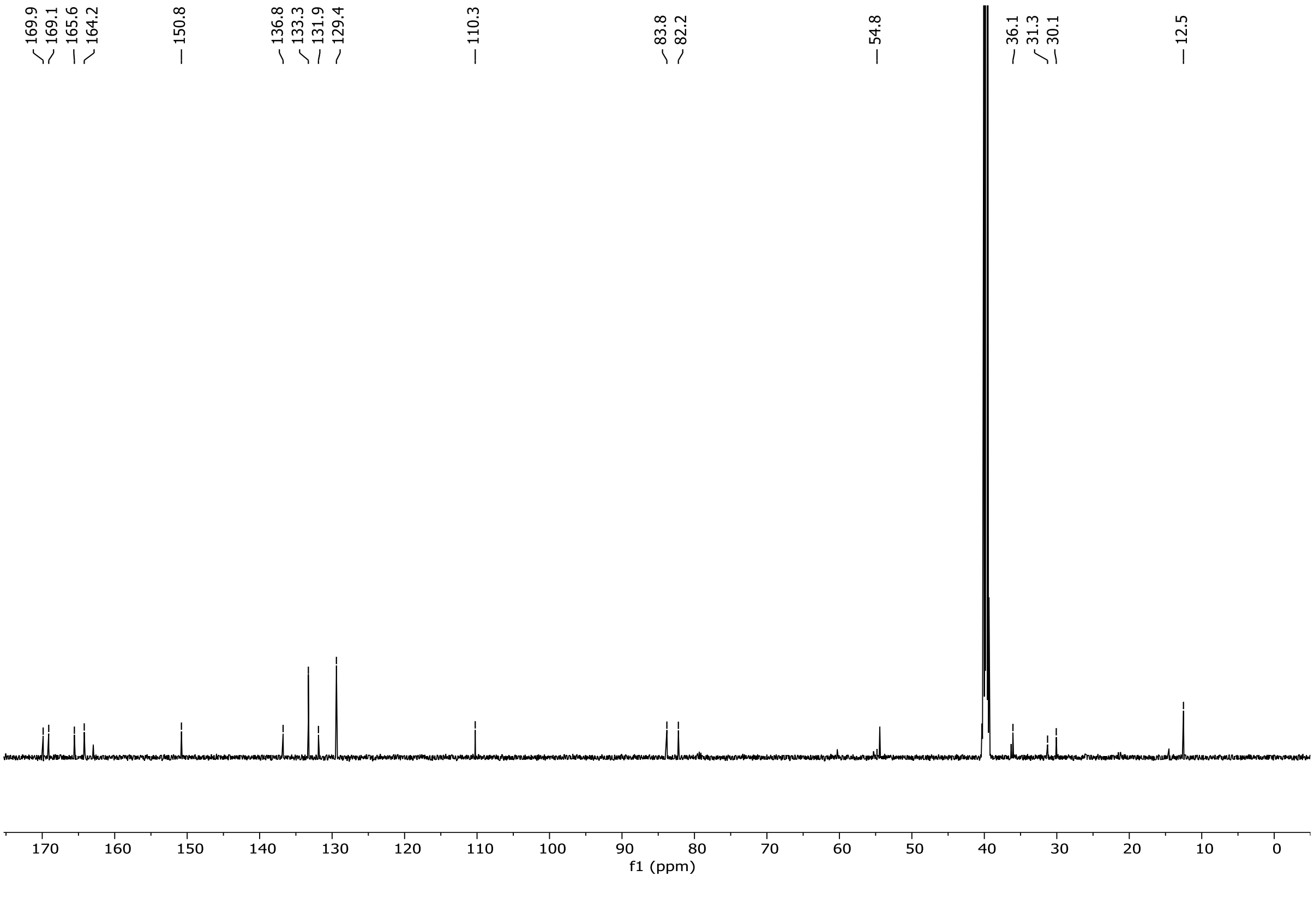

Figure S5. ^{1}H and ^{13}C NMR of the compound **5e** in $CDCl_3$ at 600 MHz and 150 MHz respectively.

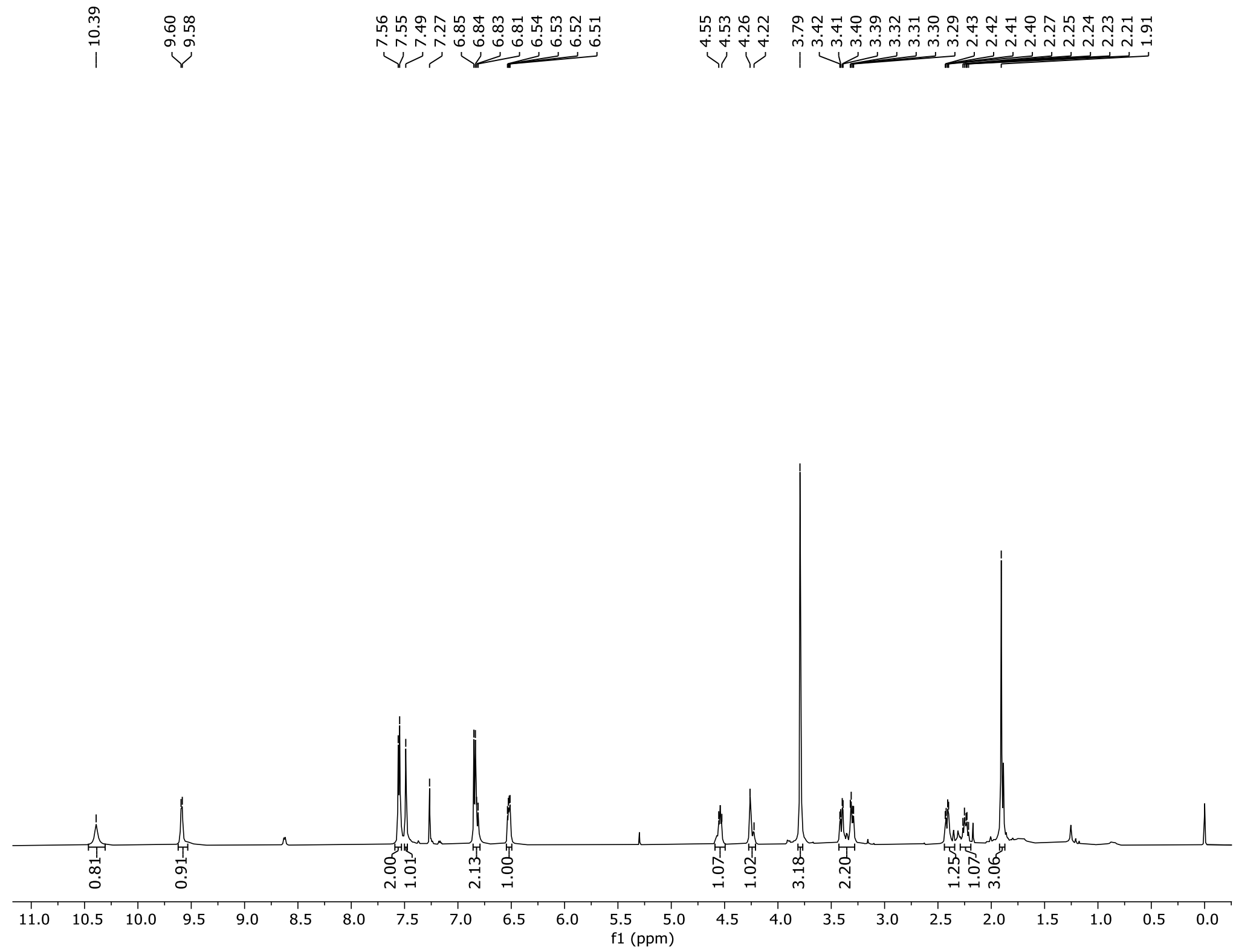

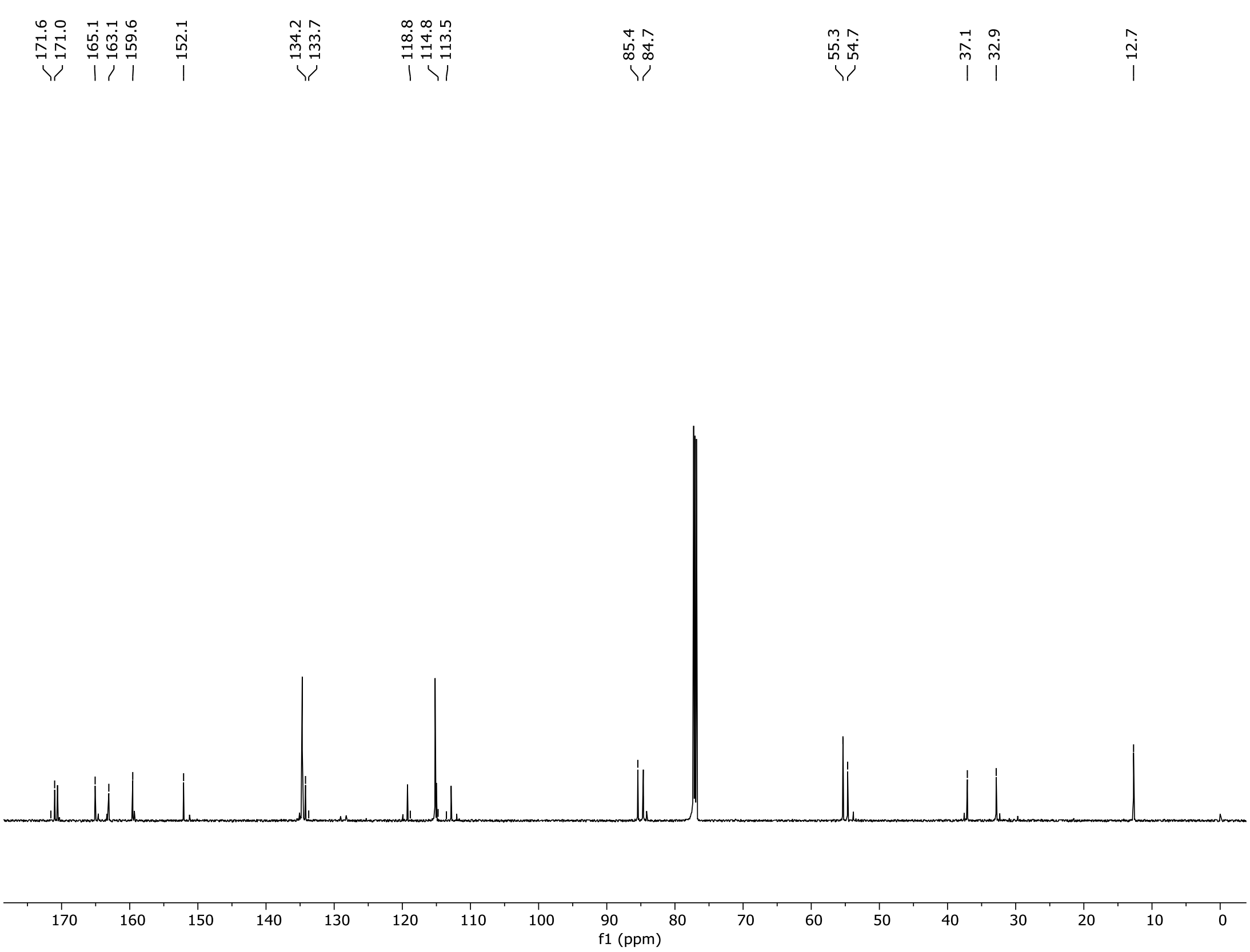

Figure S6. ^1H and ^{13}C NMR of the compound **5f** in $CDCl_3$ at 600 MHz and 150 MHz respectively.

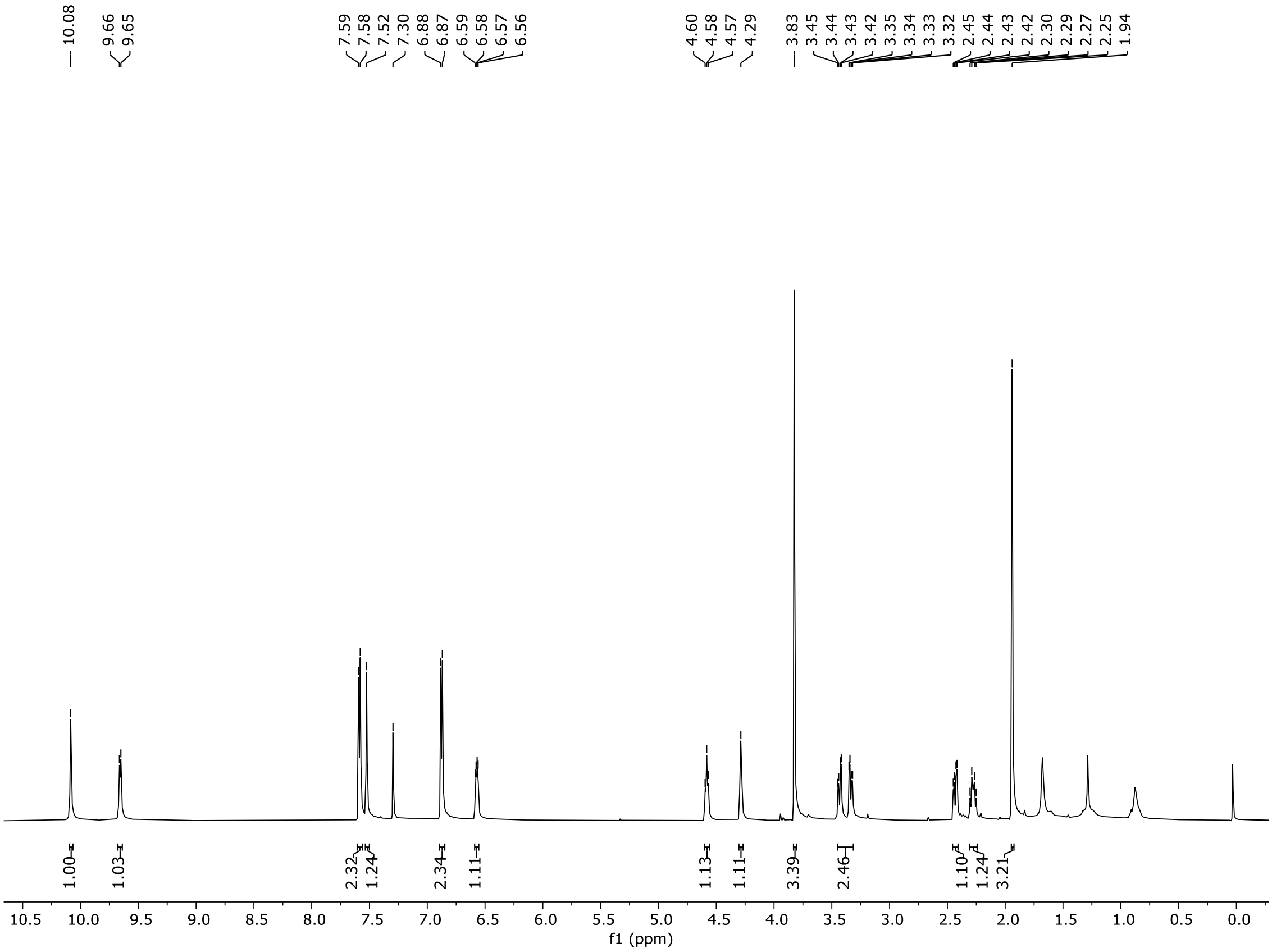

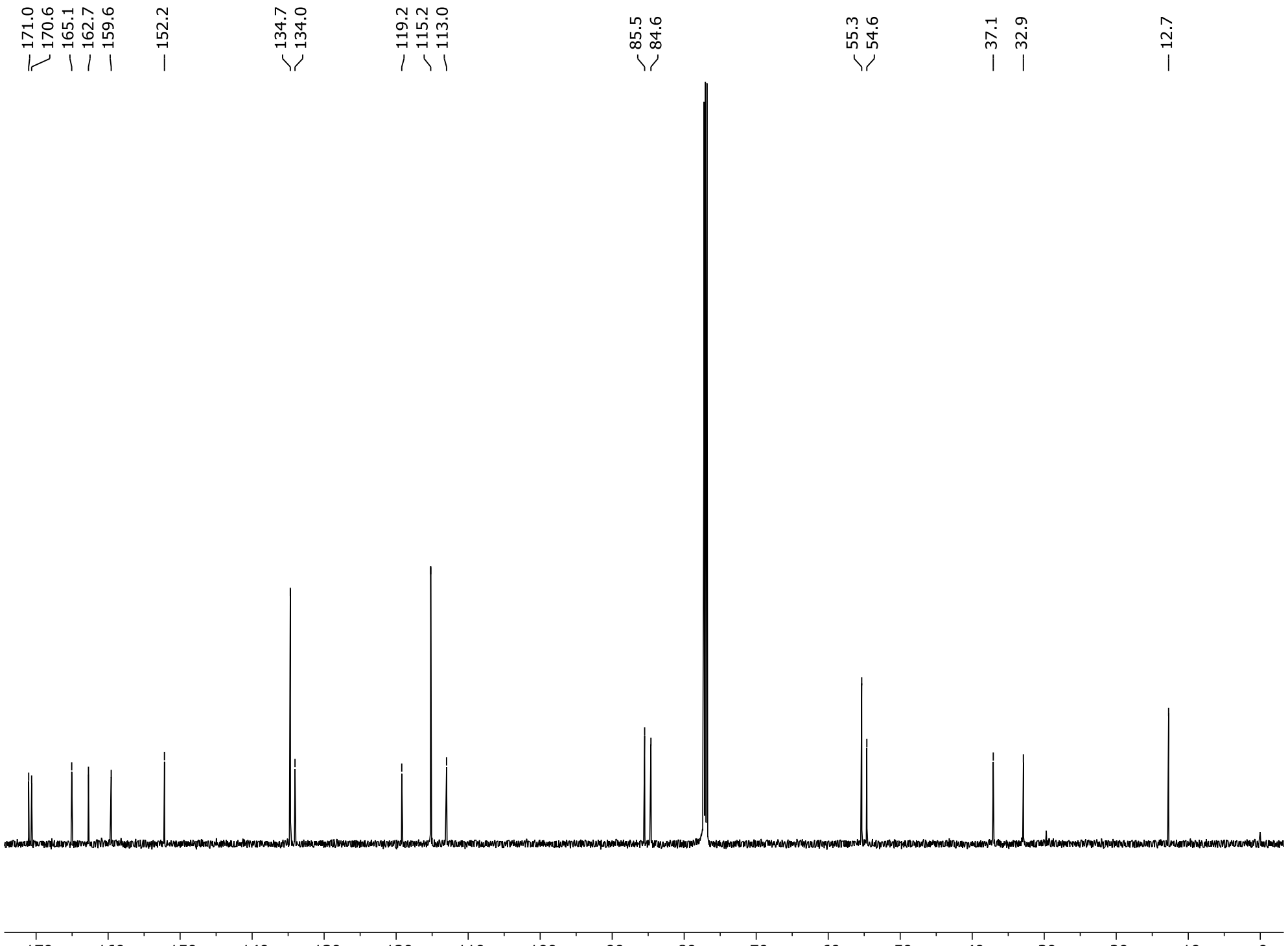

Figure S7. ^{1}H and ^{13}C NMR of the compound **5g** in $CDCl_3$ at 600 MHz and 150 MHz respectively.

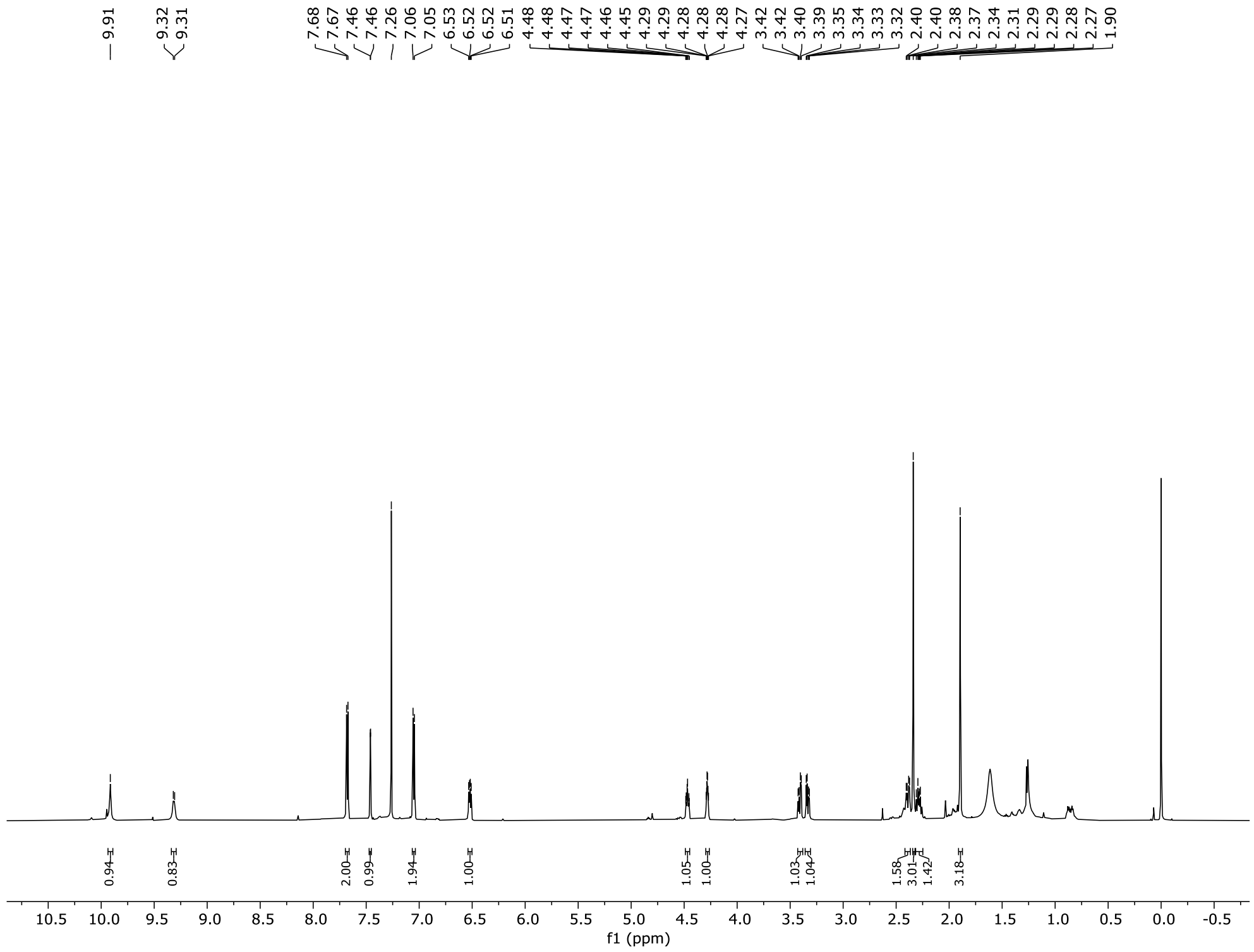

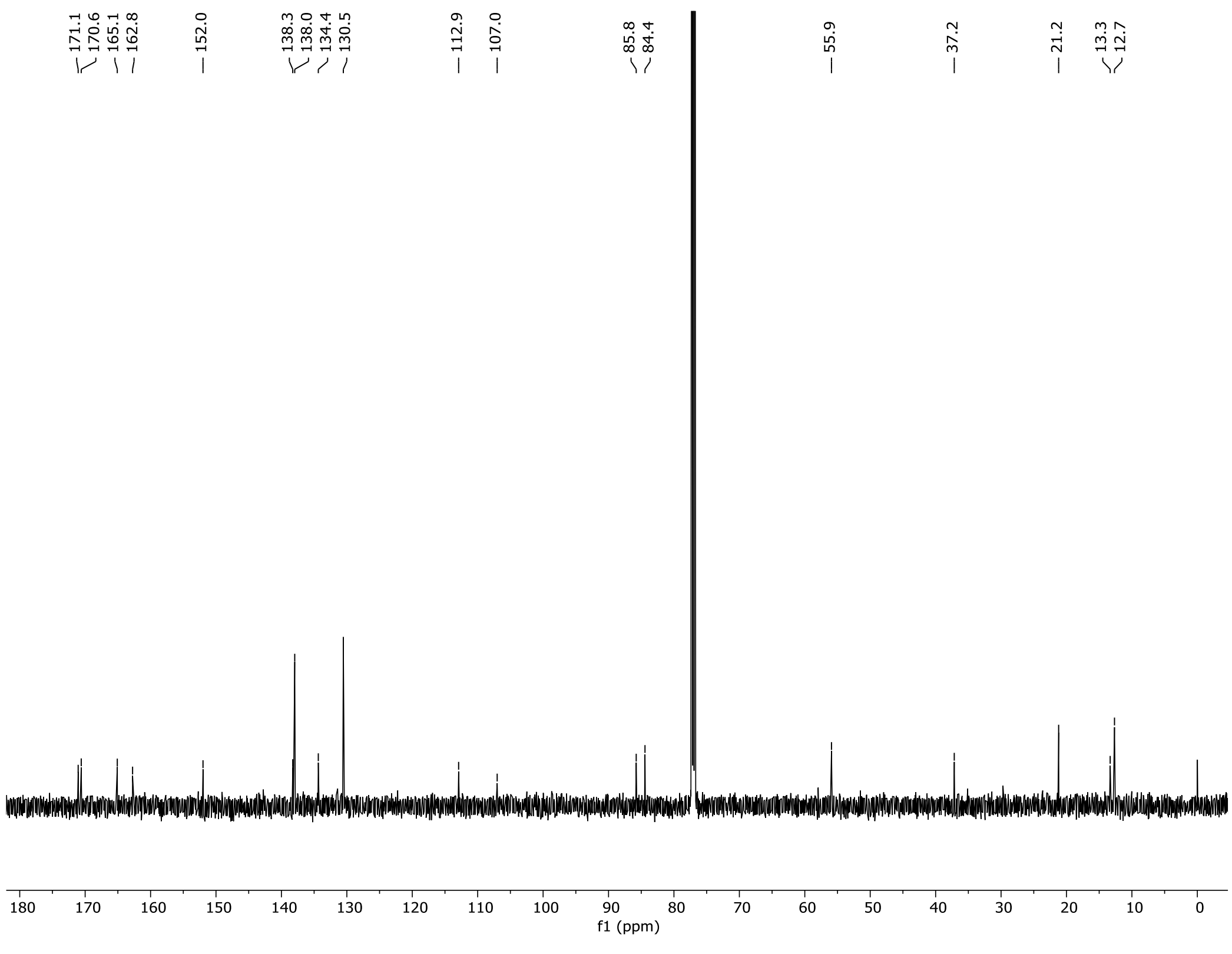

Figure S8. ^{1}H and ^{13}C NMR of the compound **5h** in $CDCl_3$ at 600 MHz and 150 MHz respectively.

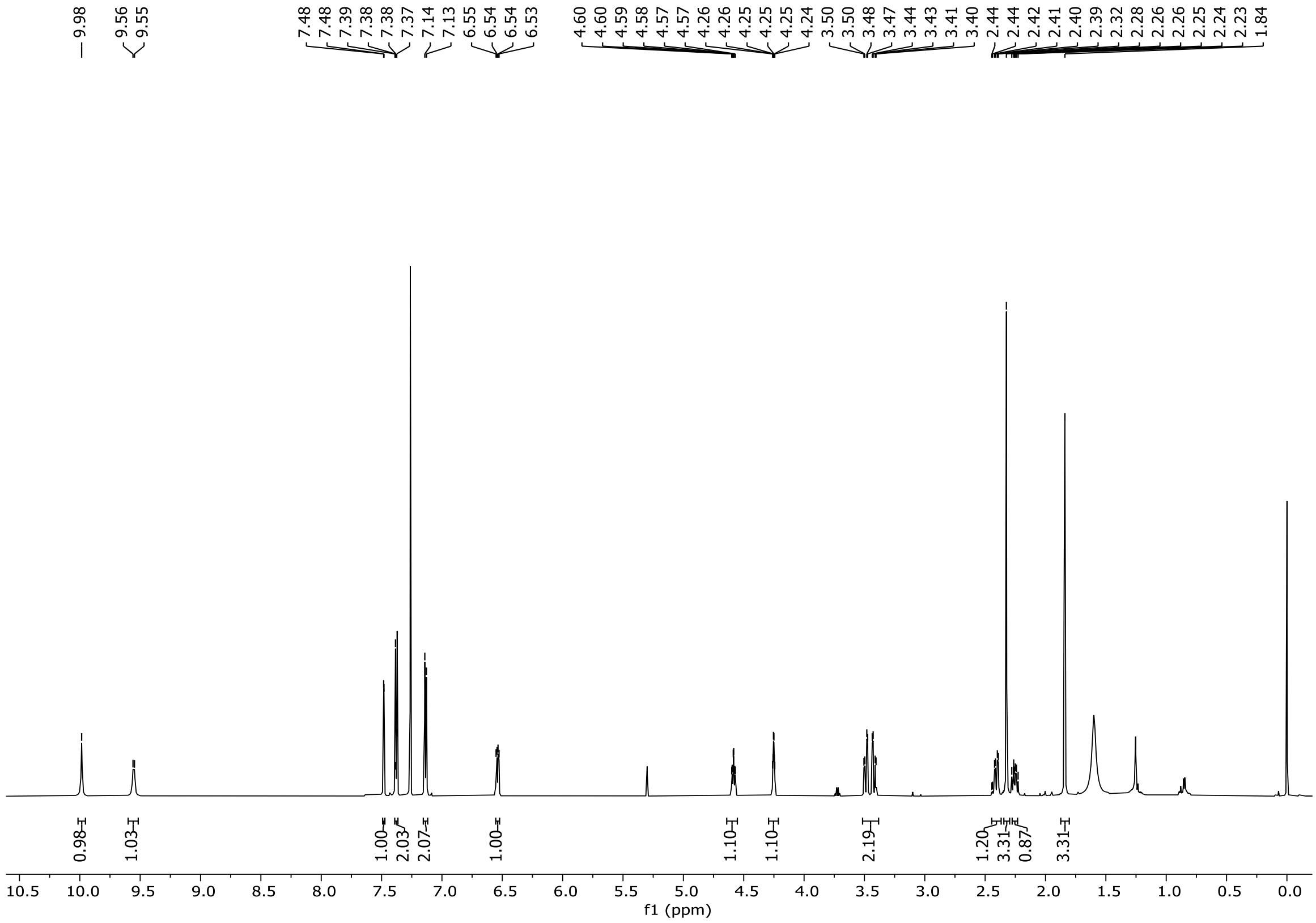

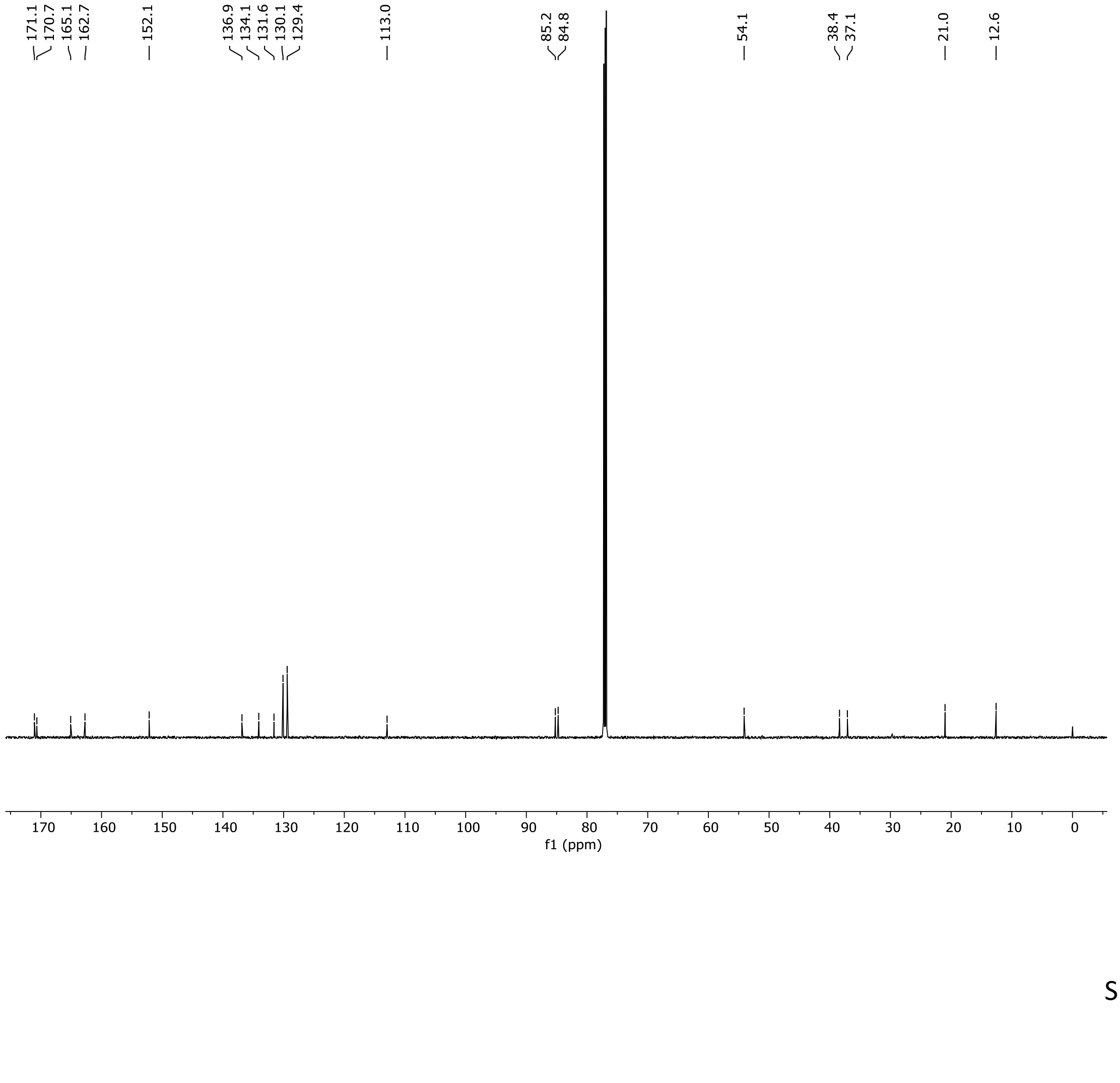

Figure S9. ^{1}H and ^{13}C NMR of the compound **5i** in DMSO-d6 at 600 MHz and 150 MHz respectively.

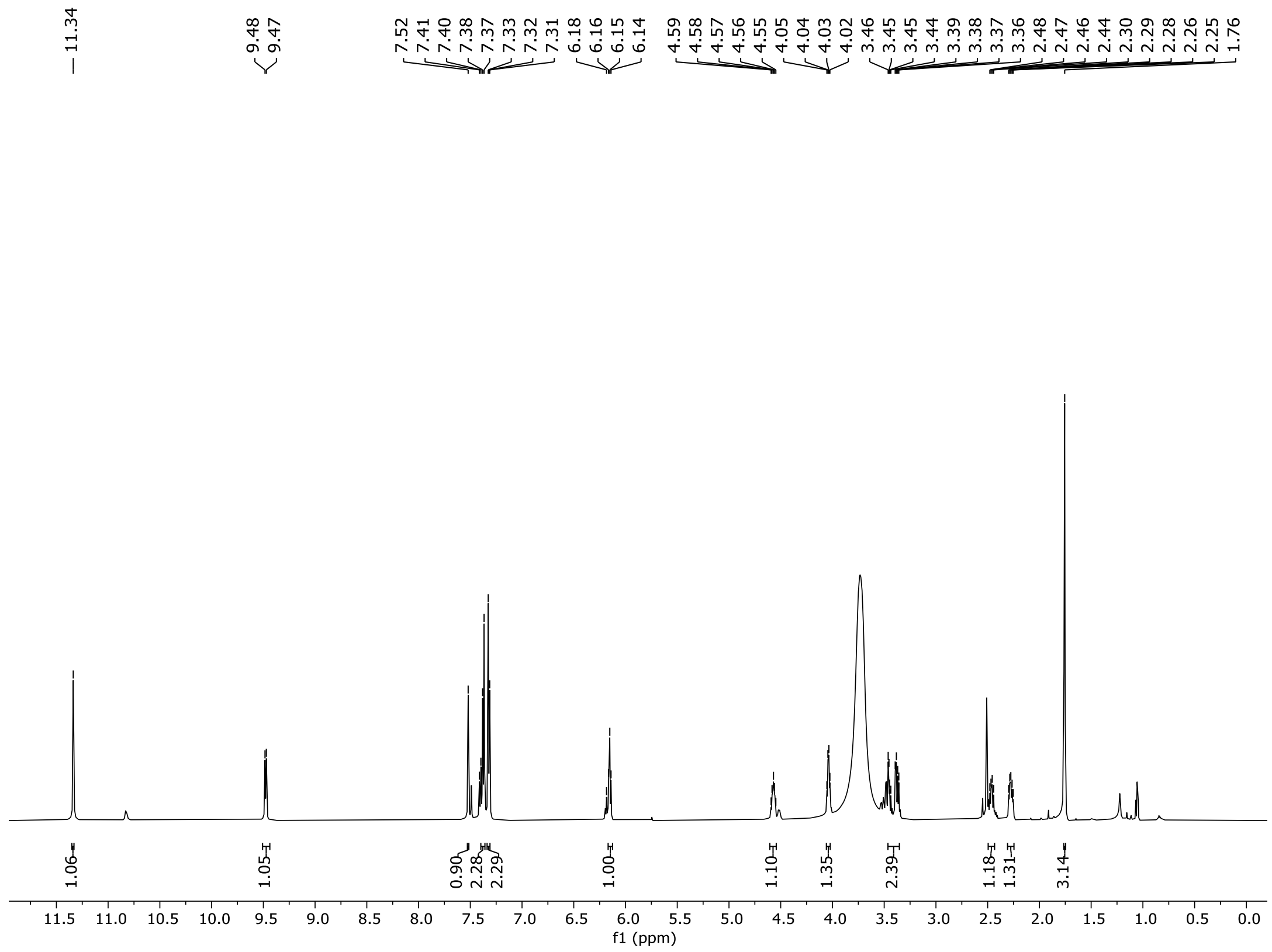

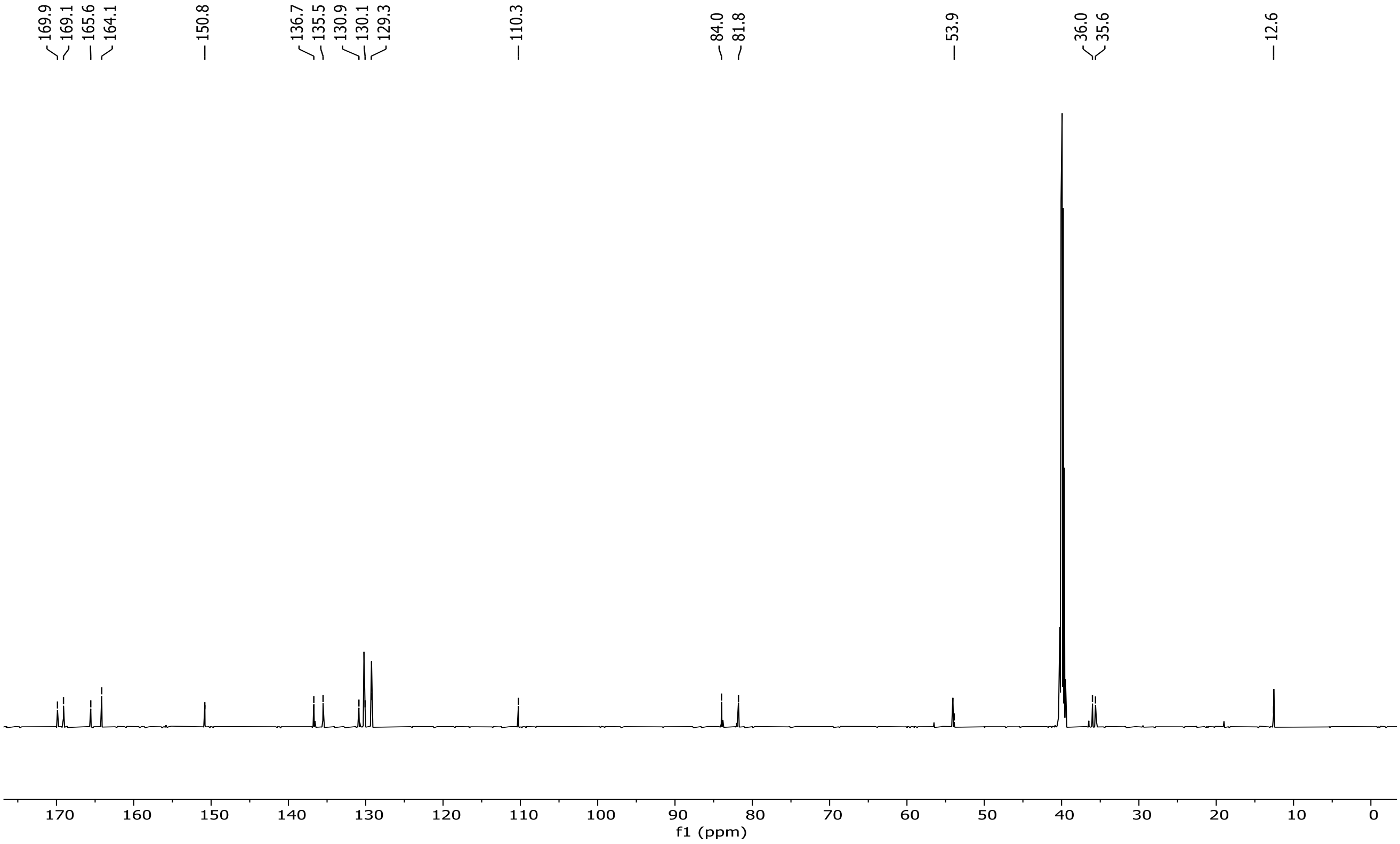

Figure S10. ^{1}H and ^{13}C NMR of the compound **5j** in $CDCl_3$ at 600 MHz and 150 MHz respectively.

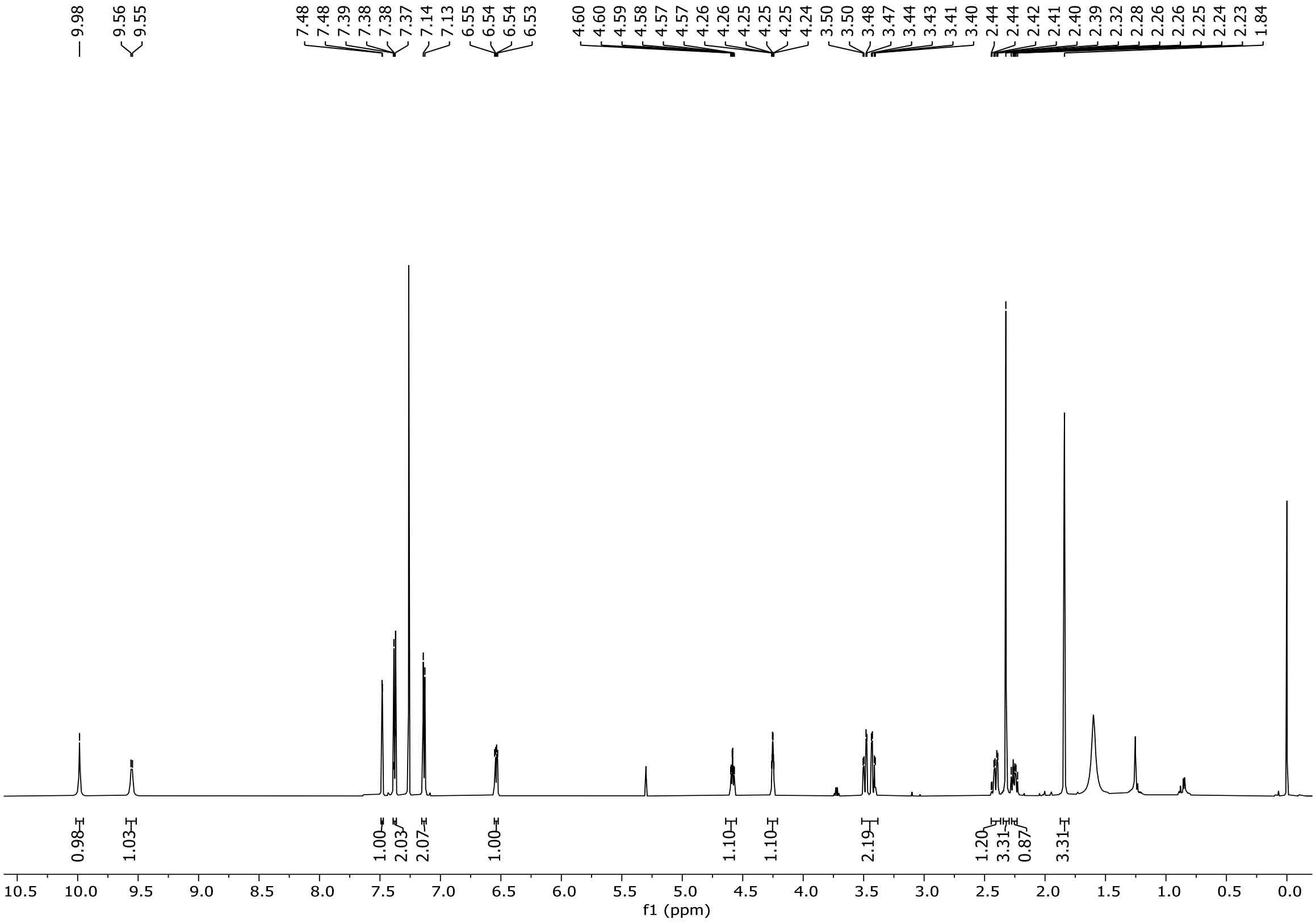

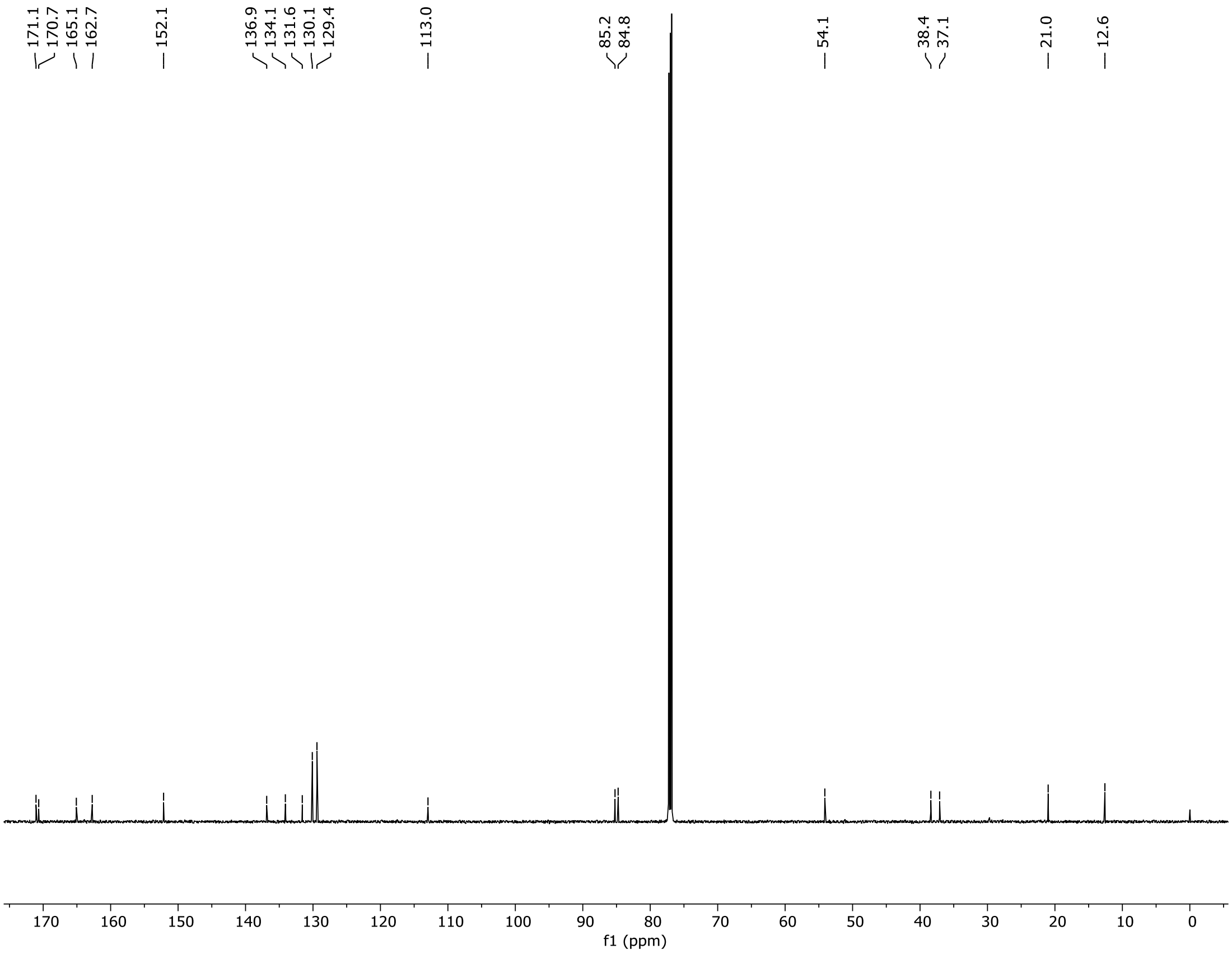

Figure S11. ^{1}H and ^{13}C NMR of the compound **5k** in DMSO-d_6 at 600 MHz and 150 MHz respectively.

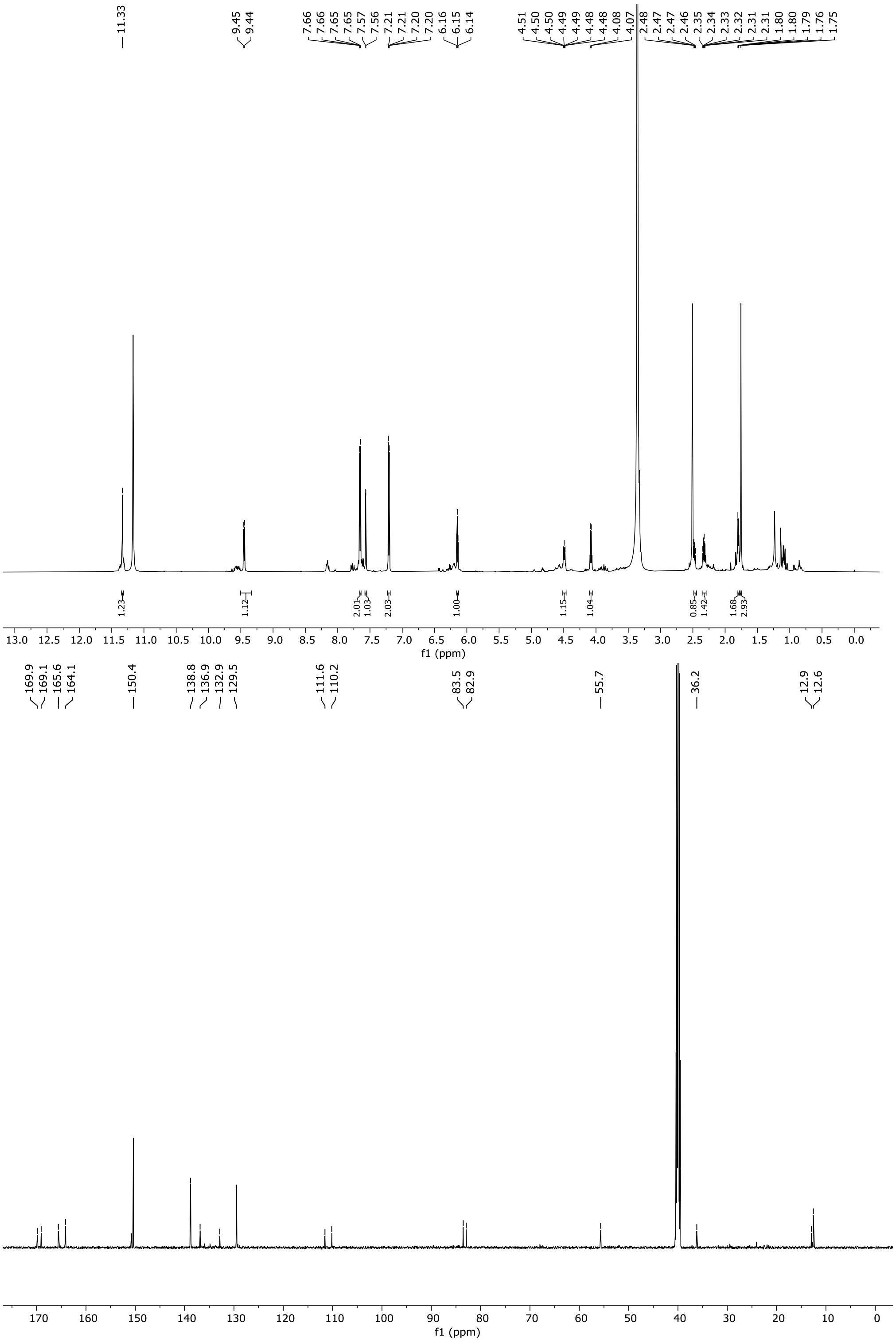

3. HRMS charts

Figure S12. HRMS of **5a** (calculated for M+H⁺: 529.0061).

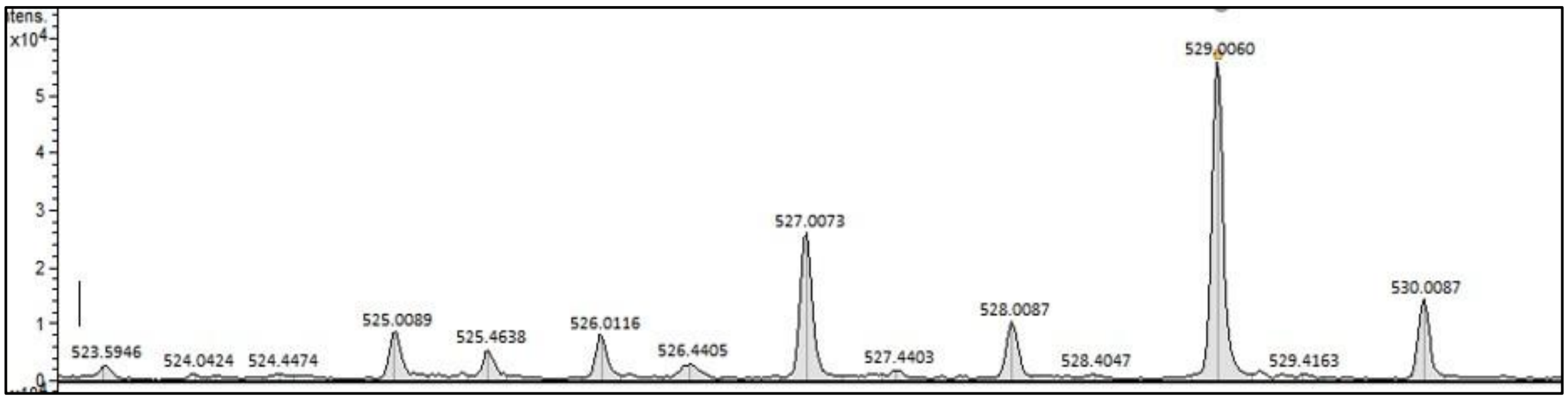

Figure S13. HRMS of **5b** (calculated for M-H⁻: 576.9801).

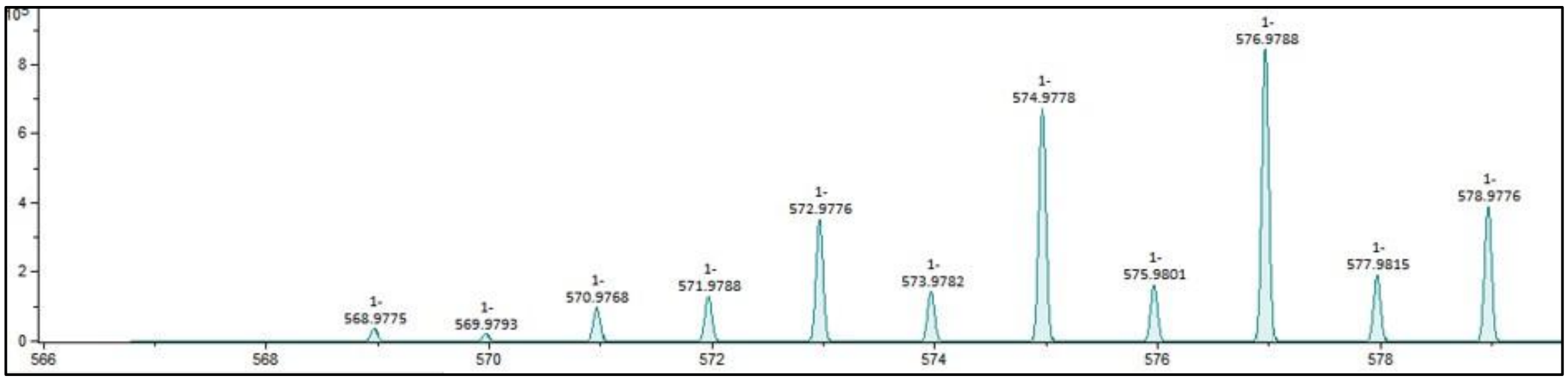

Figure S14. HRMS of **5c** (calculated for M-H⁻: 479.0460).

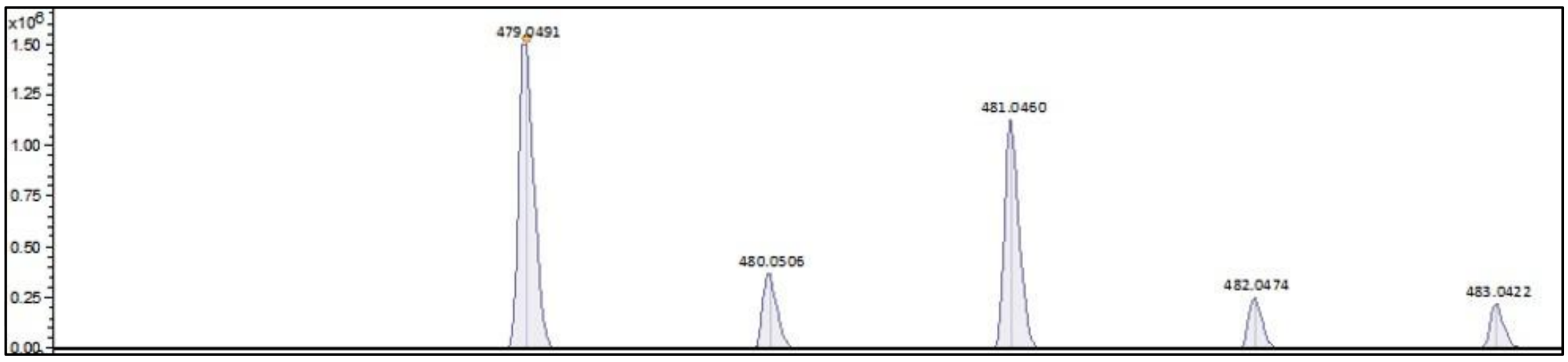

Figure S15. HRMS of **5d** (calculated for M-H⁻: 560.9516).

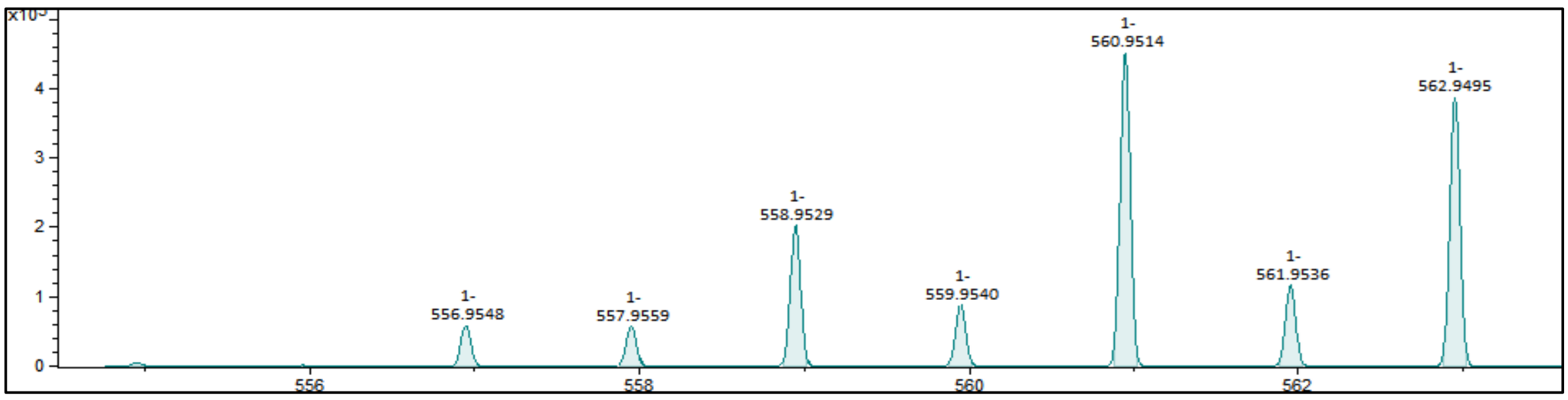

Figure S16. HRMS of **5e** (calculated for M-H⁻: 542.0139).

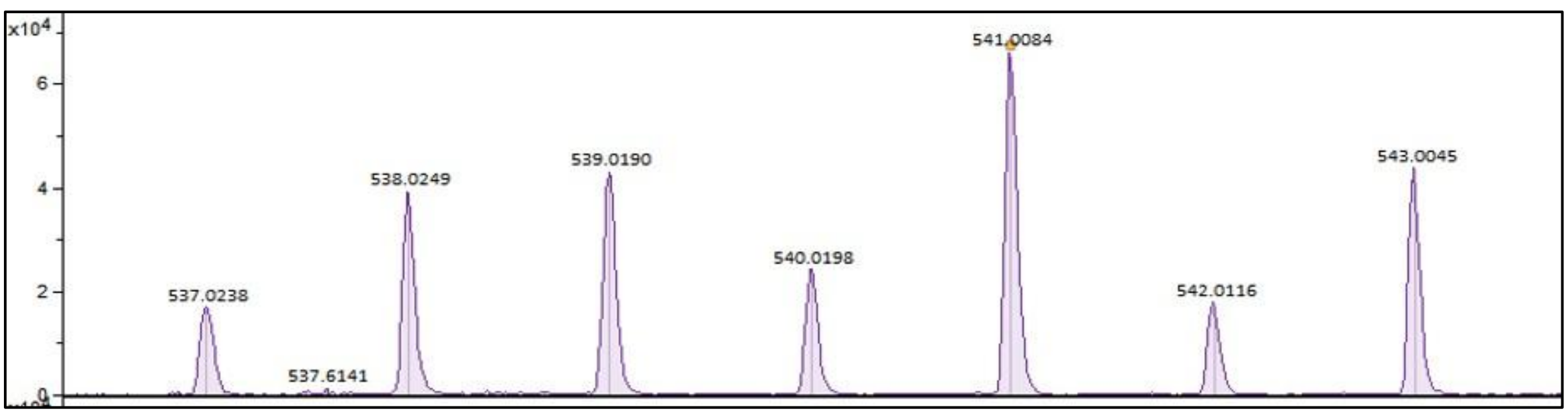

Figure S17. HRMS of **5f** (calculated for $[M+H]^+$:559.0167).

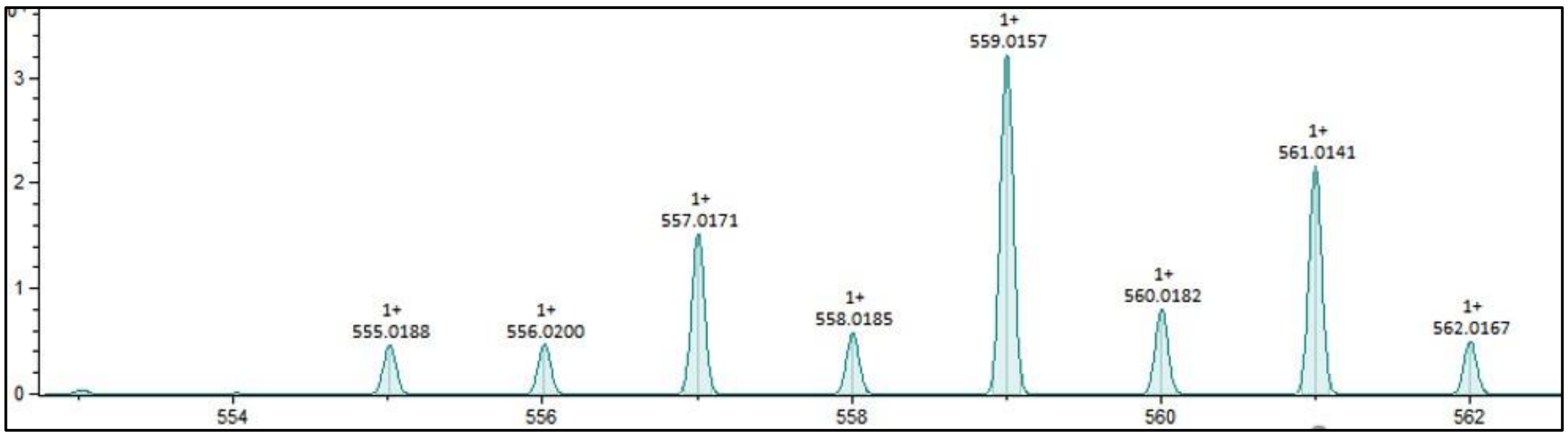

Figure S18. HRMS of **5g** (calculated for $M-H^-$:590.9958).

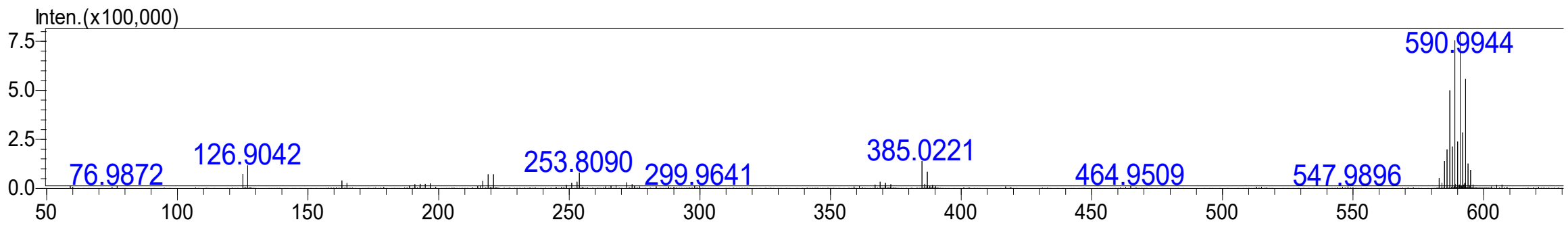

Figure S19. HRMS of **5h** (calculated for $M-H^-$: 509.0566).

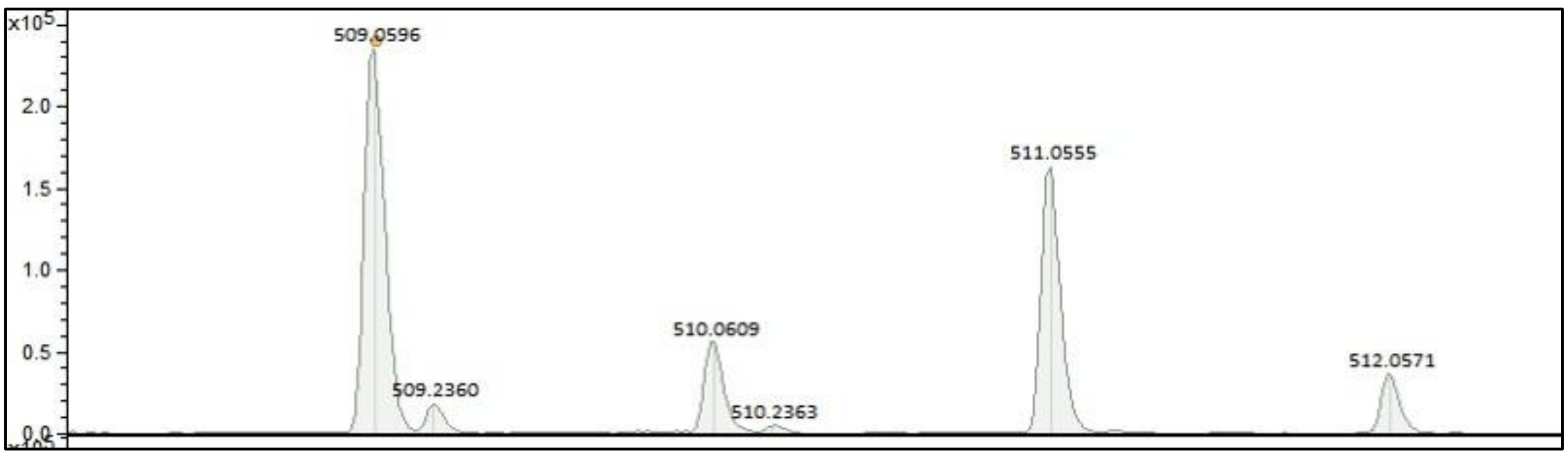

Figure S20. HRMS of **5i** (calculated for $M+H^+$: 515.0227).

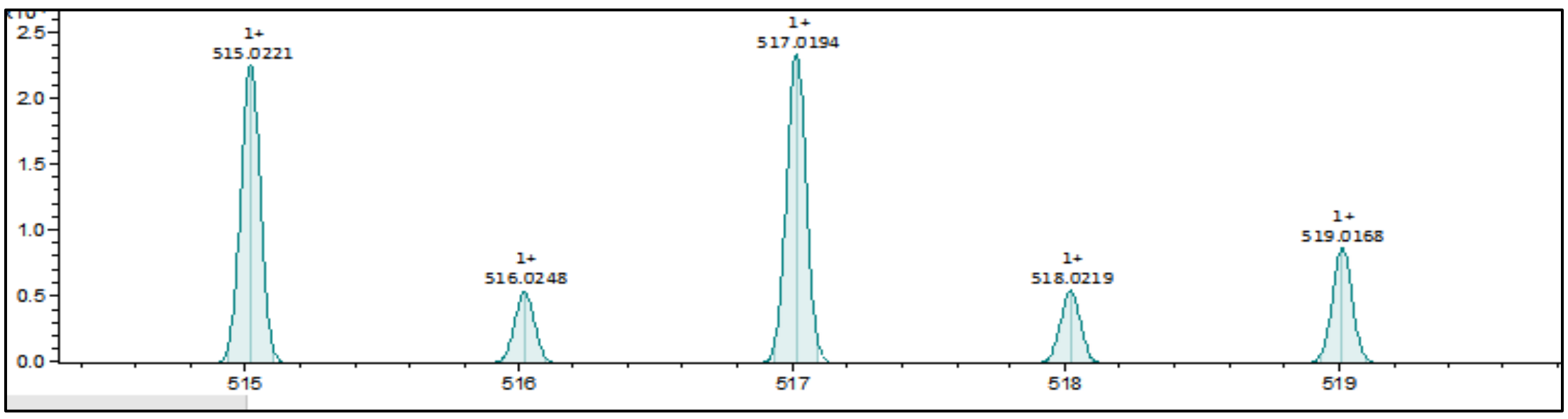

Figure S21. HRMS of **5j** (calculated for $M+H^+$:495.0773).

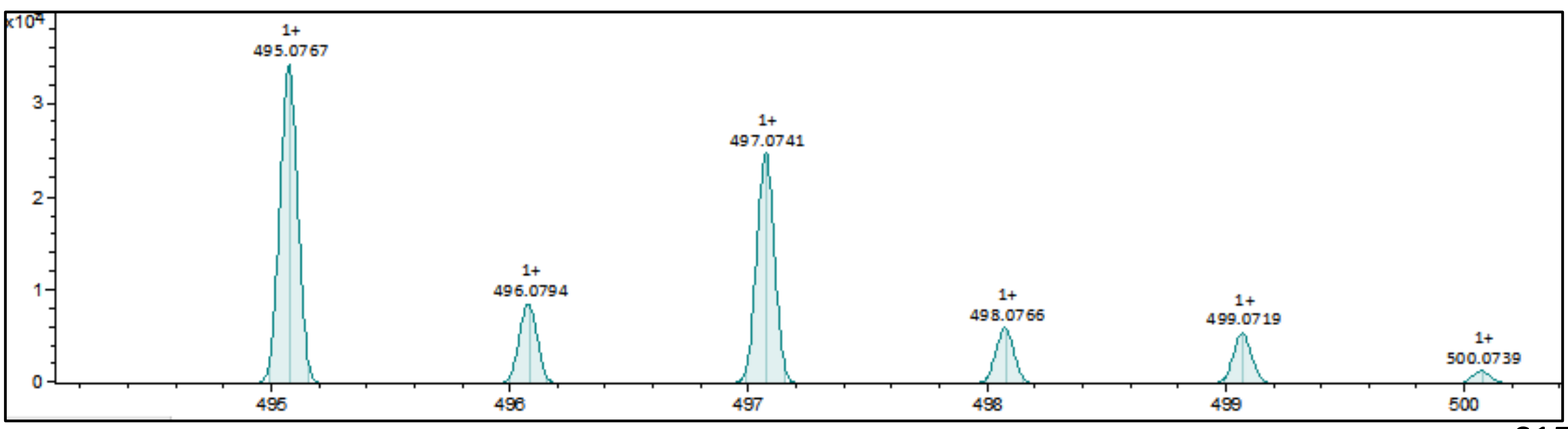

Figure S22. HRMS of **5k** (calculated for [M-H]$^-$: 610.9412)

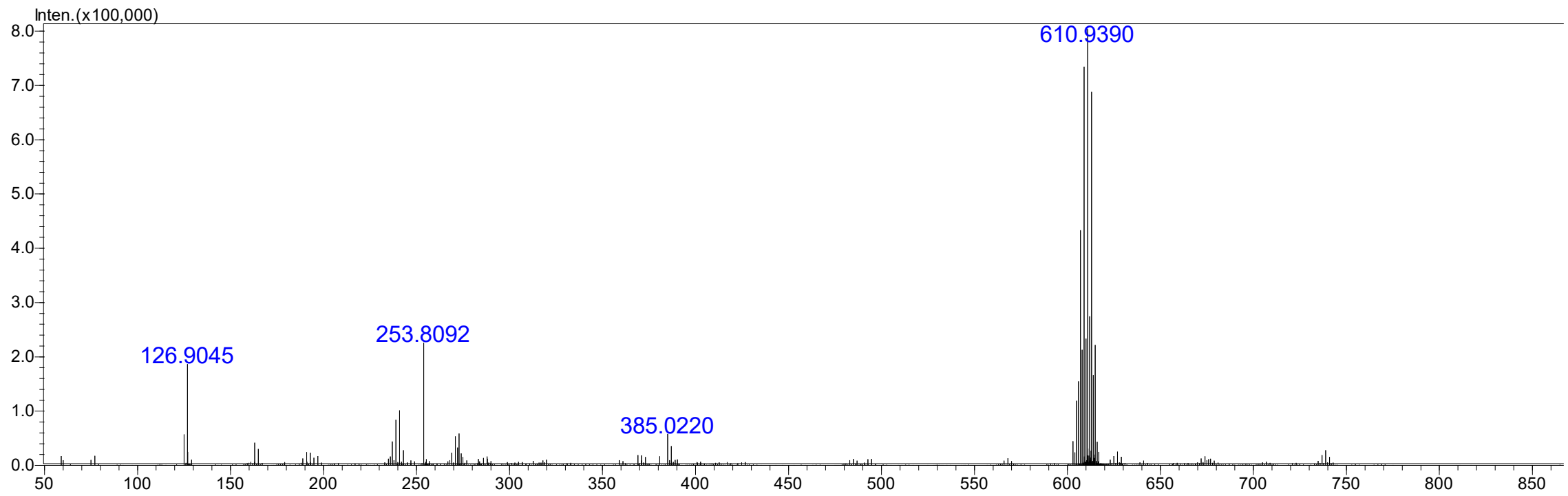

4. Interaction diagrams of docked compounds

Figure S23. Two-dimensional interaction diagram of compound **5a** bound to αβ-tubulin dimer.

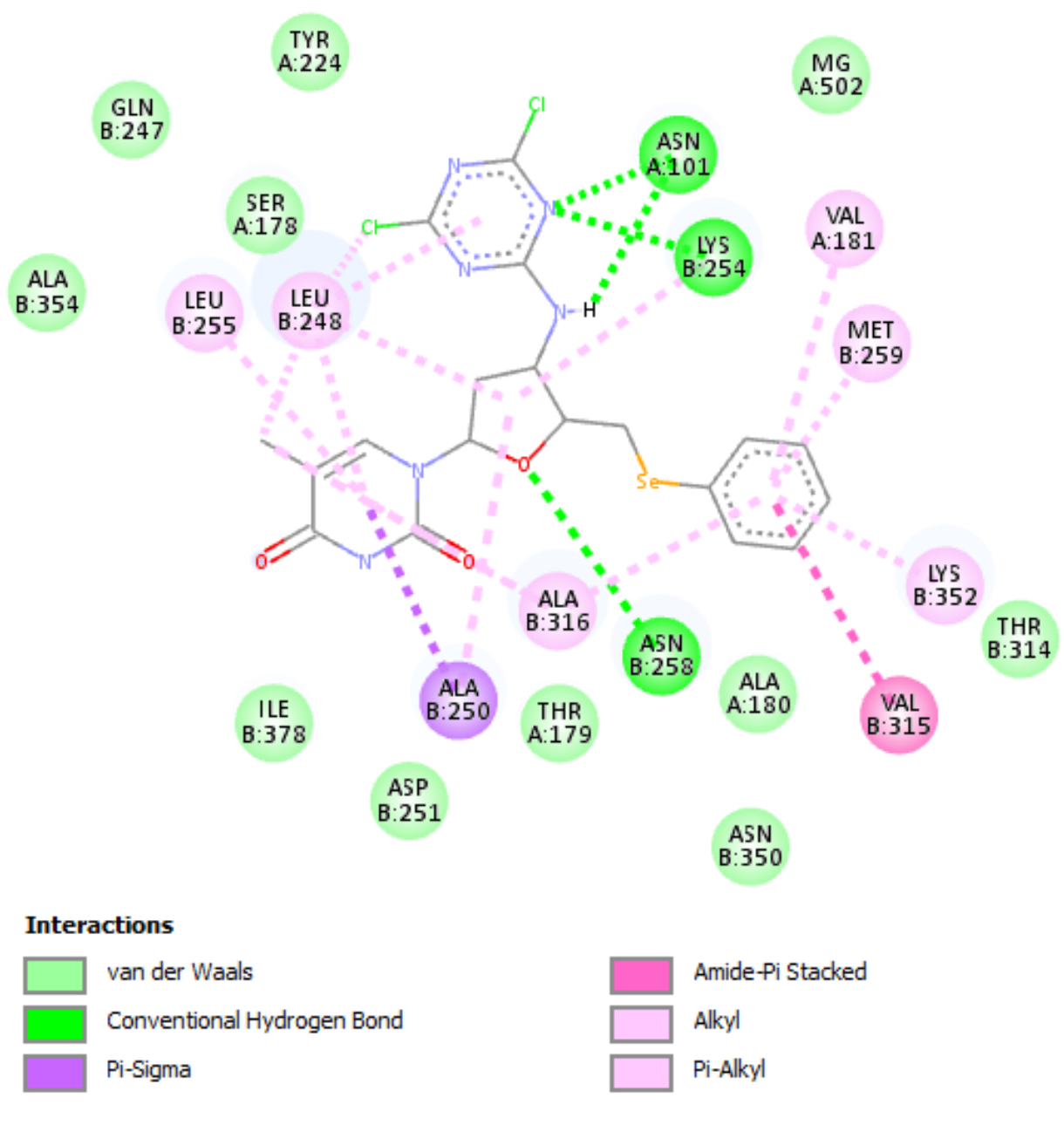

Figure S24. Two-dimensional interaction diagram of compound **5b** bound to αβ-tubulin dimer.

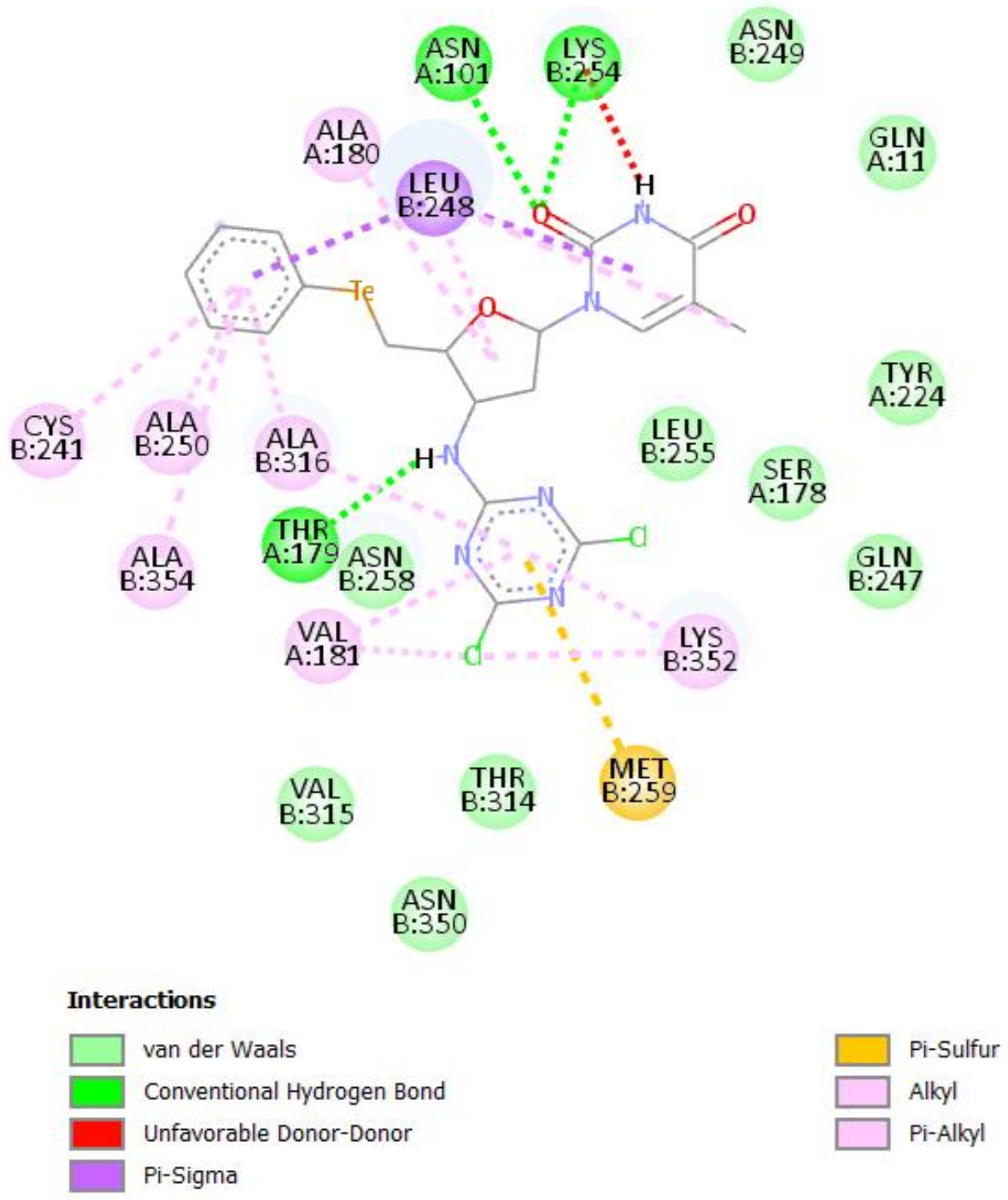

Figure S25. Two-dimensional interaction diagram of compound **5c** bound to αβ-tubulin dimer.

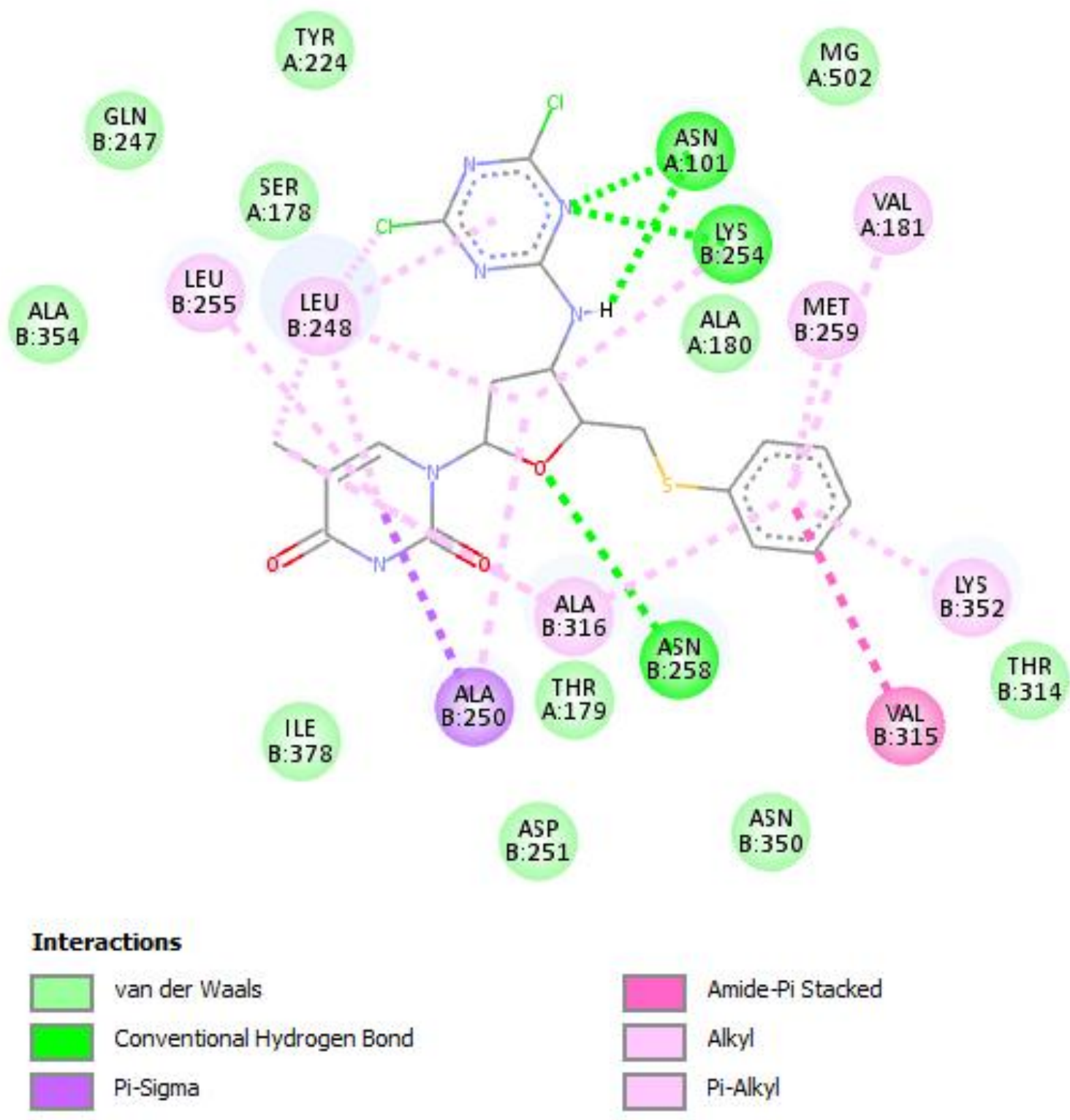

Figure S26. Two-dimensional interaction diagram of compound **5d** bound to αβ-tubulin dimer.

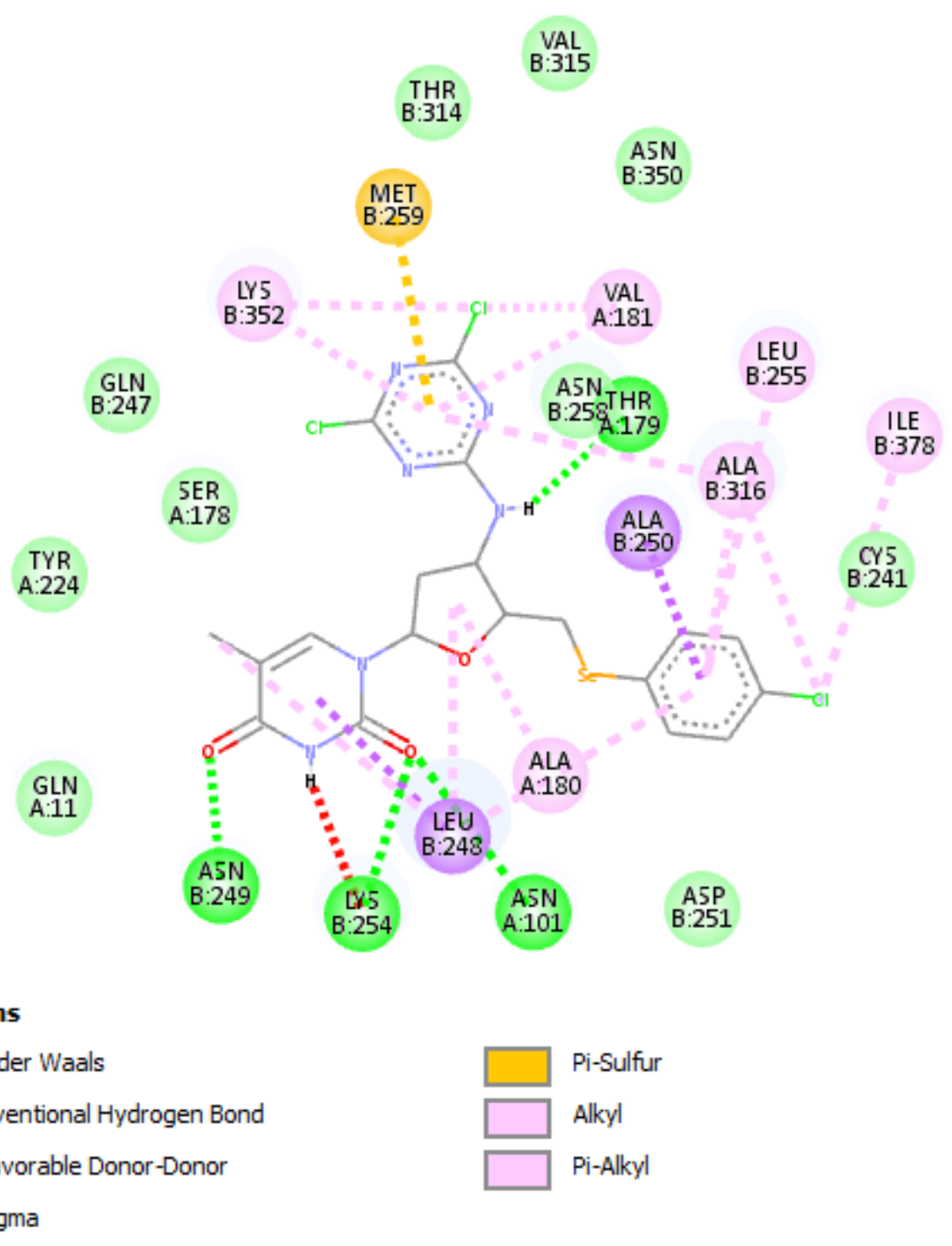

Figure S27. Two-dimensional interaction diagram of compound **5e** bound to αβ-tubulin dimer.

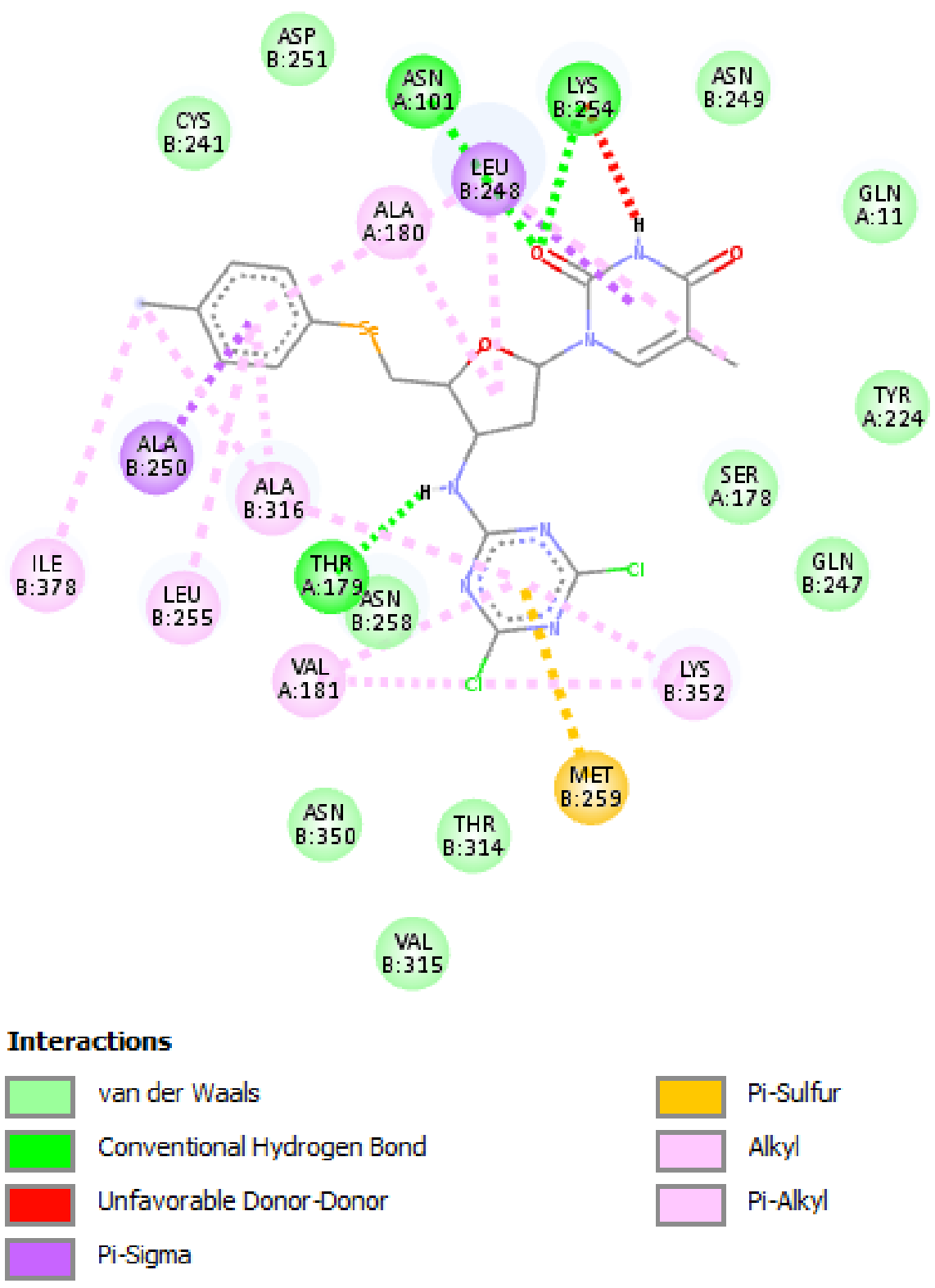

Figure S28. Two-dimensional interaction diagram of compound **5f** bound to αβ-tubulin dimer.

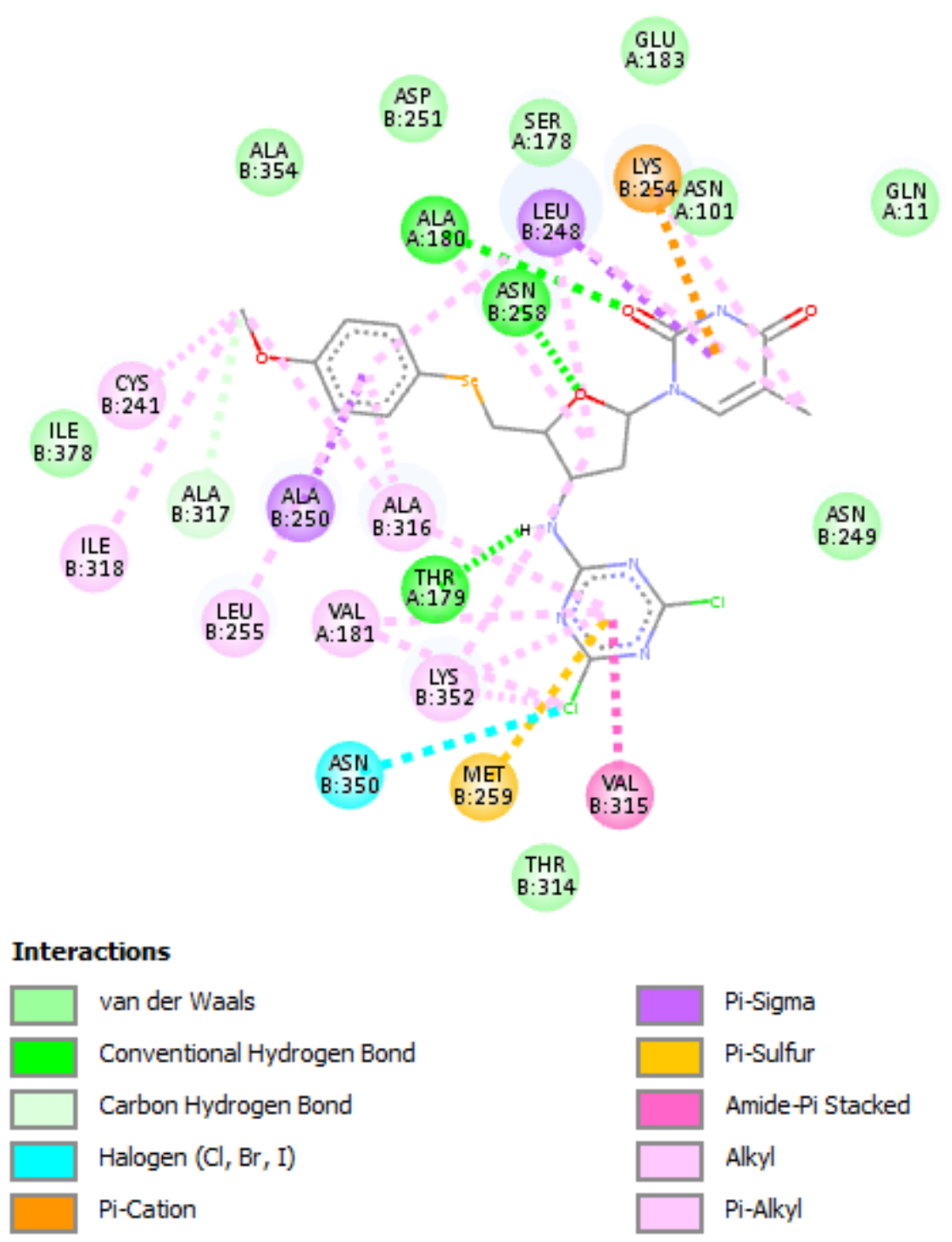

Figure S29. Two-dimensional interaction diagram of compound **5g** bound to αβ-tubulin dimer.

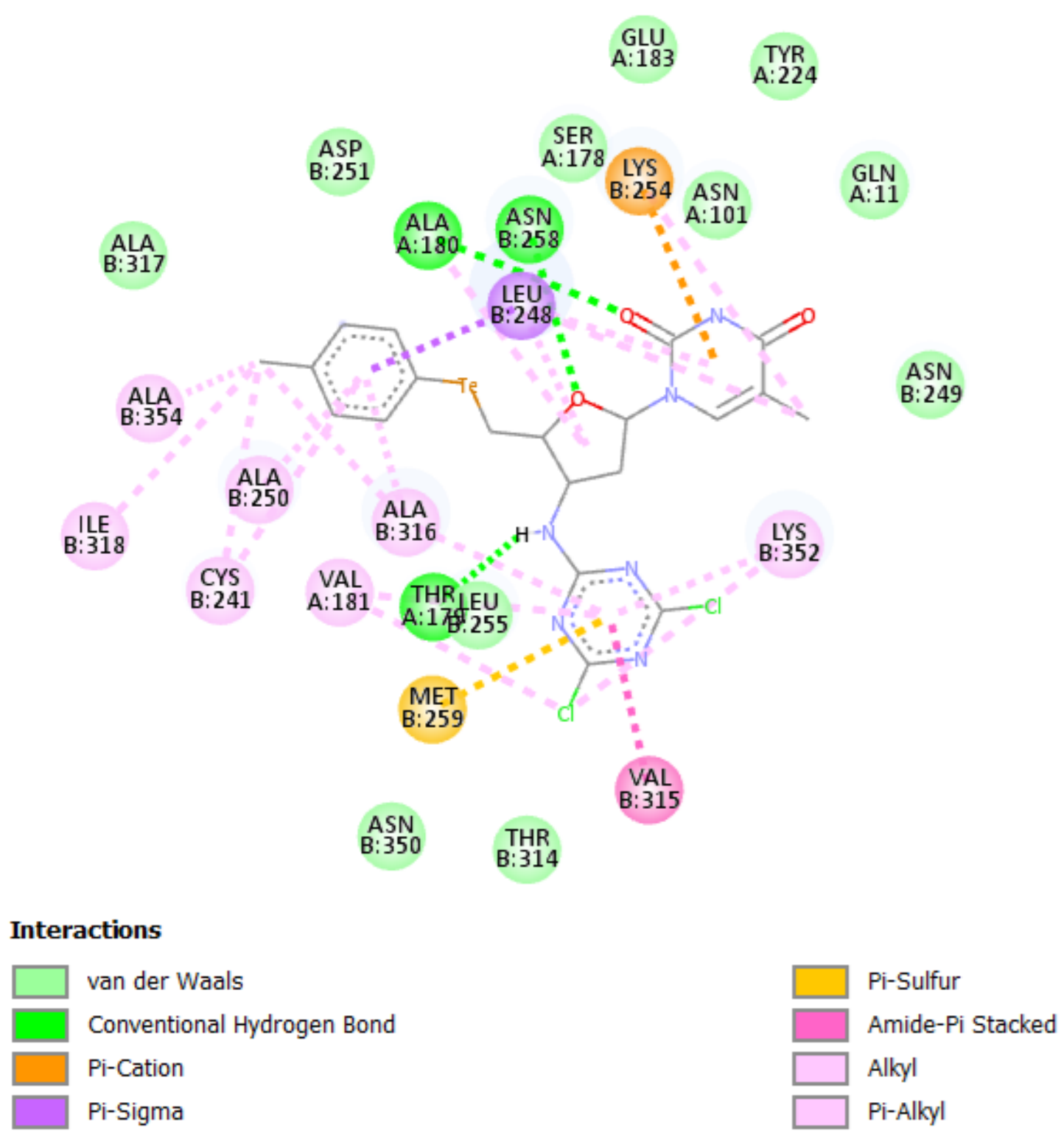

Figure S30. Two-dimensional interaction diagram of compound **5h** bound to αβ-tubulin dimer.

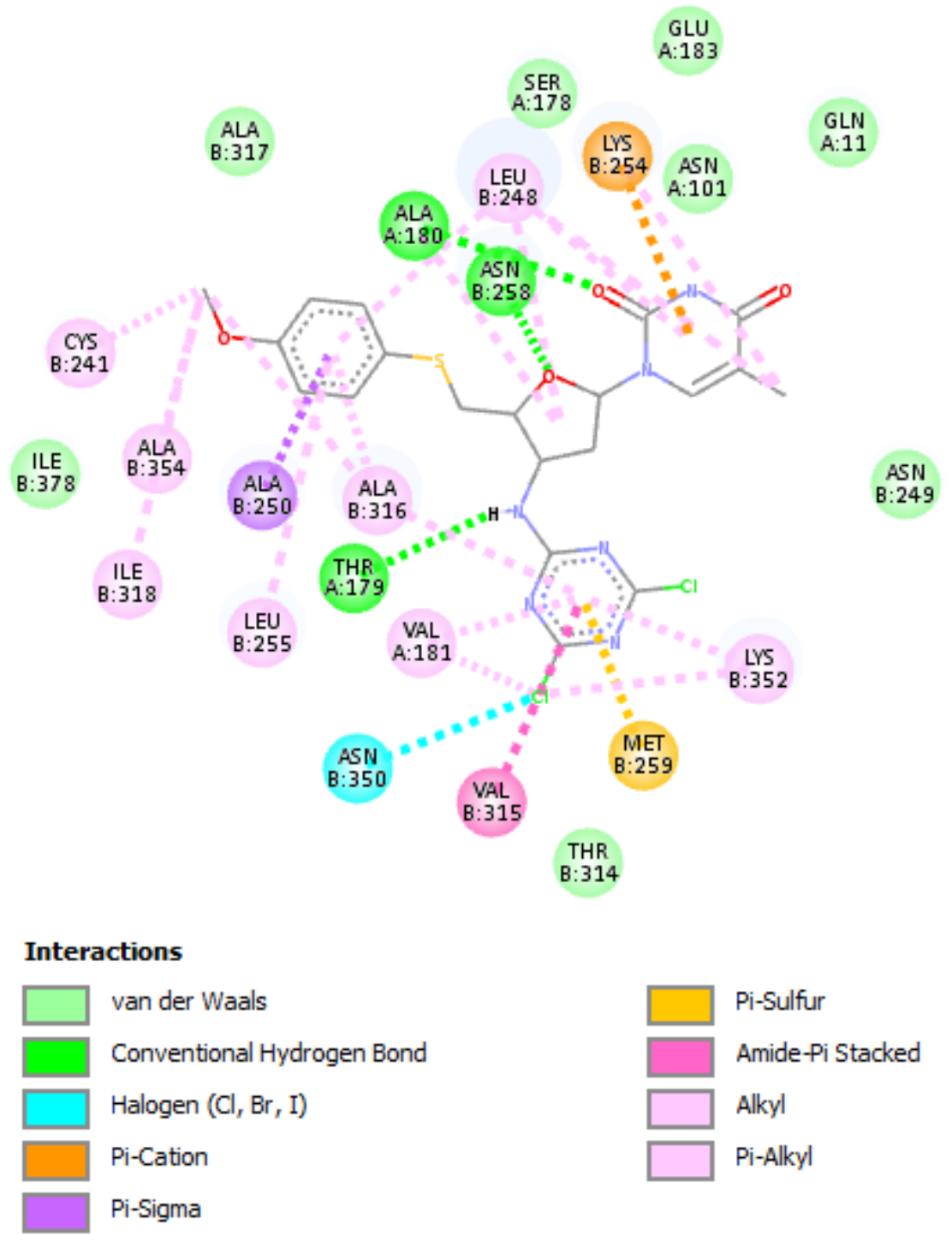

Figure S31. Two-dimensional interaction diagram of compound **5i** bound to αβ-tubulin dimer.

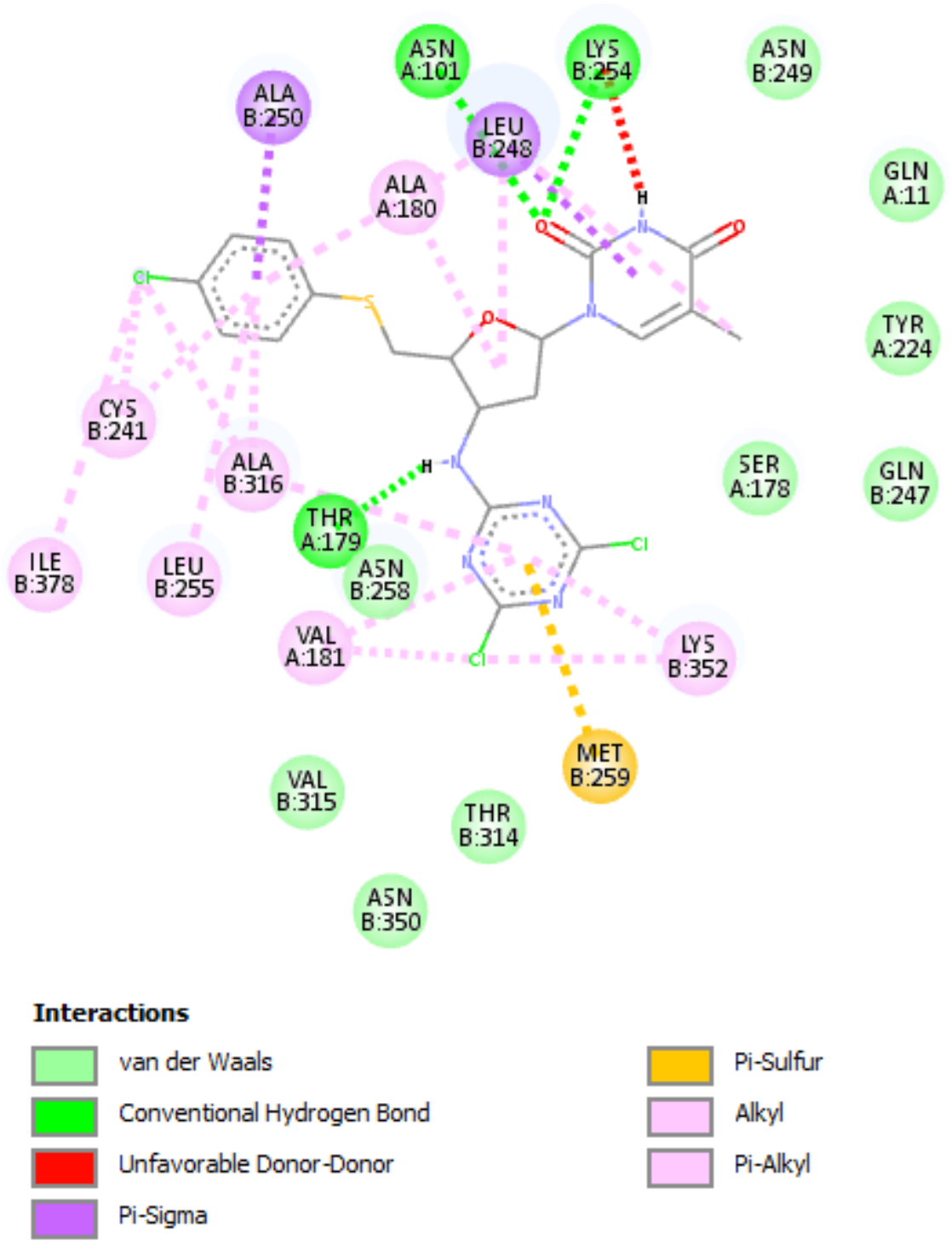

Figure S32. Two-dimensional interaction diagram of compound **5j** bound to αβ-tubulin dimer.

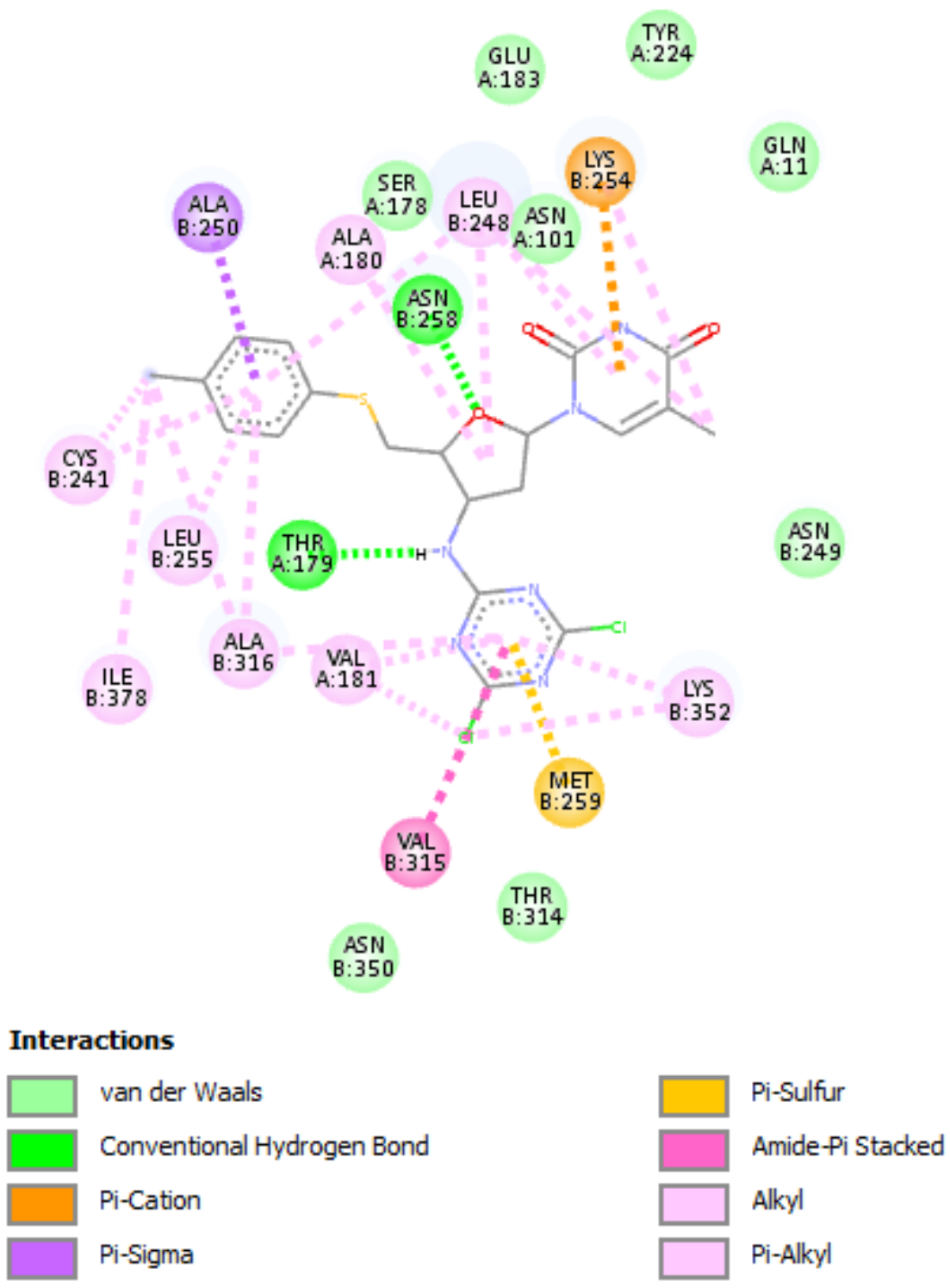

Figure S33. Two-dimensional interaction diagram of compound **5k** bound to αβ-tubulin dimer.

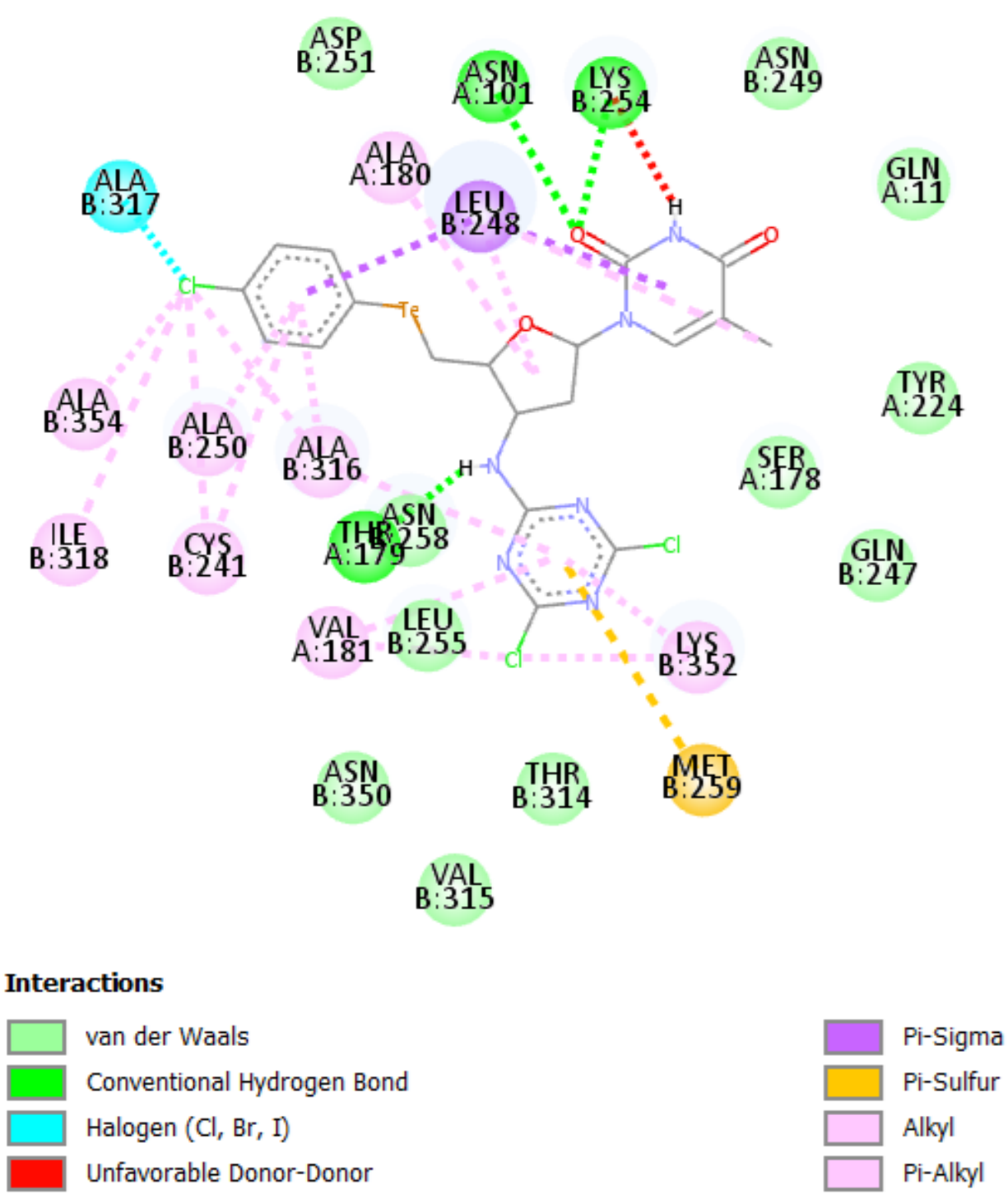

Supplement: Supplementary file 1 [file ao6c07643_si_001.pdf]
